# SNP Discovery Using BSR-Seq Approach for Spot Blotch Resistance in Wheat (*Triticum aestivum* L.), an Essential Crop for Food Security

**DOI:** 10.3389/fgene.2022.859676

**Published:** 2022-04-05

**Authors:** Ravi Ranjan Saxesena, Vinod Kumar Mishra, Ramesh Chand, Uttam Kumar, Apurba Kumar Chowdhury, Jyotika Bhati, Neeraj Budhlakoti, Arun Kumar Joshi

**Affiliations:** ^1^ Department of Genetics and Plant Breeding, Institute of Agricultural Sciences, Banaras Hindu University, Varanasi, India; ^2^ Department of Mycology and Plant Pathology, Institute of Agricultural Sciences, Banaras Hindu University, Varanasi, India; ^3^ Borlaug Institute for South Asia (BISA), Ludhiana, India; ^4^ Department of Plant Pathology, Uttar Banga Krishi Vishwavidyalaya, Coochbehar, India; ^5^ ICAR-Indian Agricultural Statistics Research Institute, New Delhi, India; ^6^ International Maize and Wheat Improvement Center (CIMMYT) and Borlaug Institute for South Asia (BISA), DPS Marg, New Delhi, India

**Keywords:** wheat, food security, spot blotch, RILs, bulk segregant analysis, SNPs

## Abstract

The pathogenic fungus, *Bipolaris sorokiniana*, that causes spot blotch (SB) disease of wheat, is a major production constraint in the Eastern Gangetic Plains of South Asia and other warm, humid regions of the world. A recombinant inbred line population was developed and phenotyped at three SB-prone locations in India. The single nucleotide polymorphism (SNP) for SB resistance was identified using a bulked segregant RNA-Seq-based approach, referred to as “BSR-Seq.” Transcriptome sequencing of the resistant parent (YS#24), the susceptible parent (YS#58), and their resistant and susceptible bulks yielded a total of 429.67 million raw reads. The bulk frequency ratio (BFR) of SNPs between the resistant and susceptible bulks was estimated, and selection of SNPs linked to resistance was done using sixfold enrichments in the corresponding bulks (BFR >6). With additional filtering criteria, the number of transcripts was further reduced to 506 with 1055 putative polymorphic SNPs distributed on 21 chromosomes of wheat. Based on SNP enrichment on chromosomal loci, five transcripts were found to be associated with SB resistance. Among the five SB resistance-associated transcripts, four were distributed on the 5B chromosome with putative 52 SNPs, whereas one transcript with eight SNPs was present on chromosome 3B. The SNPs linked to the trait were exposed to a tetra-primer ARMS-PCR assay, and an SNP-based allele-specific marker was identified for SB resistance. The *in silico* study of these five transcripts showed homology with pathogenesis-related genes; the metabolic pathway also exhibits similar results, suggesting their role in the plant defense mechanism.

## Introduction

As a primary staple crop, the importance of wheat is well-documented in food security and provides nutrition for more than 35% of the world’s population (FAO, 2018). Great progress has been achieved in wheat production since the Green Revolution; however, due to climate change and the popularization of dwarf and semidwarf varieties, many of the biotic factors, including the pathogen of spot blotch (SB) disease have gained importance in countries such as India (Joshi et al., 2007a). The prevalence of SB is more common in the wheat-producing countries, notably in the Eastern Gangetic Plains of India, Nepal, Bangladesh, China, and South America (Joshi et al., 2007b; Gupta et al., 2018; Singh et al., 2018). A hemi-biotrophic fungus, *Bipolaris sorokiniana* (Sacc.), causes SB disease in wheat, seedling blight, common root rot, seedling rot, and seed rot (Acharya et al., 2011). Crop yield losses in the Indian subcontinent alone are estimated to be in the range of 15%–25% (Dubin and Van Ginkel, 1991; Pandey et al., 2021), but the level of loss in individual fields can be much higher. In South Asia, the disease is expected to inflict a 15%–20% average yield loss (Duveiller and Sharma, 2009); however, under favorable conditions, more than 85% of losses are reported in Zambia during the summer season (Raemaekers, 1987). Thus, SB disease might have a significant impact on global food security. Seed treatments and foliar fungicidal sprays are both recommended for the treatment of SB disease. Although strong-efficacy fungicides are available to manage SB disease, their application may have adverse effects on human health and the environment (Monyo et al., 2009; Castro et al., 2018). Other than health and environmental constraints, the use of fungicides led to an increase in the cost of cultivation and a reduction in farmers’ income. Therefore, the most successful, cost-efficient, and environmentally friendly strategy to control the disease is to use cultivars with host resistance (Gupta et al., 2018; Zhang et al., 2020). However, breeding for SB resistance has been slow due to the quantitative nature of inheritance (Joshi et al., 2004) and is often influenced by the environment (Joshi et al., 2007b). In such a situation, genetics, and genomics-based technology aid in developing resistant plants more efficiently. Classic methods of mapping QTLs/genes, involve phenotyping and genotyping of segregating mapping populations with polymorphic markers identified between parents. However, the identification of polymorphic markers between contrasting parents is a time-consuming and tedious task (Schneeberger and Weigel, 2011; Jaganathan et al., 2020). So far, SSRs, or microsatellite markers, are used most widely to map wheat genomes for SB resistance (Singh and Singh, 2015). Presently, several QTLs and four genes (*Sb1-Sb4*) have been identified as having major effects on SB resistance (Gupta et al., 2018). Gene *Sb1* is present on chromosome 7DS, where it shares space with *Lr34* (Lillemo et al., 2013), *Sb2* on chromosomes 5BL (Kumar et al., 2015), *Sb3* on 3BS (Lu et al., 2016), and a recently discovered *Sb4* are present on 4BL (Zhang et al., 2020).

The identification of genetic regions and the development of robust molecular markers in wheat has long been hampered by its hexaploidy nature (AABBDD), which has the large sizes of the subgenomes and more than 85% repeated sequences (Paux et al., 2012; Wicker et al., 2018). It is difficult to design single-copy markers because the level of polymorphism is quite low in wheat compared with other cereals (Paux et al., 2012). Liu et al. (2012) propose a new genetic mapping strategy called “BSR-Seq,” which combines bulked segregant analysis with RNA-Seq. BSR-Seq is a technique that involves sequencing RNAs from extreme bulks for the trait of interest. The method is especially important for crops with large and complex genomes, such as wheat, where resequencing is still prohibitively expensive (Liu et al., 2012; Xie et al., 2020). BSR-Seq can also be used to fine-map crops that do not yet have a reference genome sequence (O’Neil and Emrich, 2013). RNA-Seq captures the full range of dynamic spectrum of the transcriptome, advantageous over array platforms that are restricted to the predefined set of variants incorporated into the array design. SNPs can be identified either by aligning to a known transcriptome or by *de novo* assembly over the transcriptome (Grabherr et al., 2011; O’Neil and Emrich, 2013). RNA-Seq is more likely to discover functional SNPs than other SNP discovery methods (Pootakham et al., 2014). Genotyping by RNA-Seq can detect much more variation compared with array-based technology because it covers 70%–90% of the total genes based on the tissue and development stage of the sample. For the development of constitutive markers, the combination of advanced sequencing technology with BSA provides a powerful tool for the rapid identification of genes or causal mutations (Xu and Bai, 2015; Zou et al., 2016).

BSR-Seq has been applied successfully to localize the candidate gene for grain protein content (GPC) gene *GPC-B1* in wheat to 0.4 cM from 30 cM (Trick et al., 2012). The glossy 3 *(gl3)* gene of maize was allocated to a ∼2 Mb area by BSR-Seq, and a single gene, MYB transcription factor, was found (Liu et al., 2012). Ramirez-Gonzalez et al. (2015) identified putative single nucleotide polymorphisms (SNPs) for the *Yr15* locus using BSR-Seq and mapped this gene to a 0.77-cM interval that imparts resistance to yellow rust in wheat. Pearce et al. (2016) used RNA-Seq to analyze and compare the transcriptomes of phyB-null and phyC-null TILLING mutants and identified 82 genes that are significantly upregulated or downregulated in both types of mutants. In a more recent study on BSR-Seq (Klein et al., 2018), cloned mutant genes in maize were involved in plant growth by delineating mapping intervals. Compared with the entire population analysis, BSA provides a shortcut to identifying and developing markers for a trait. The substantial reduction in the cost of sequencing, particularly with the introduction of BSR-Seq, may be accomplished by genotyping several bulks from a large-sized population, and the power of detection can be significantly improved, particularly for alleles of interest or rare alleles (Hiebert et al., 2014; Zou et al., 2016). As a result, BSR-Seq is widely used for the quick finding of genes and markers associated with the target gene.

To meet future food demands, climatic resilience and disease-resistant wheat combined with good agronomic value can potentially improve its productivity (Mondal et al., 2016). To date, SB resistance of wheat is quantitative, involving genes and many QTLs with low-coverage linkage maps. To reduce the loss of wheat productivity and grain quality caused by SB, new resistance genes must be identified. Here, the present investigation was initiated with an objective to identify putative SNPs for SB resistance by the “BSR-Seq” approach.

## Materials and Methods

### Plant Materials

A total of 211 single seed descent (SSD)-derived recombinant inbred lines (RILs) were generated from the cross YS#24 × YS#58. The parental lines YS#24 and YS#58 are stable RILs selected from the Yangmai6 × Sonalika cross and advanced to the F_12_ generation. Yangmai6 is a Chinese source of SB resistance, and Sonalika is a susceptible cultivar of Mexican origin that has been under cultivation in India for more than five decades/during the green revolution. The parents of the RILs used in this study are similar with respect to the agronomical and phenological traits but harbor different SB resistance QTLs (Kumar et al., 2009). To develop RILs, field trials in the crop season were conducted on the Agricultural Research Farm, BHU, Varanasi, whereas off-season nurseries were raised for generation advancement at Wellington, Tamil Nadu, India. The whole procedure of RIL development and its evaluation is mentioned in the flow chart (Supplementary Figure S1).

### Phenotypic Evaluation of RILs for SB Resistance

The 211 RILs (F_5_ and F_6_) were evaluated at three hot spots in India, namely, Agricultural Research Farm, BHU, Varanasi (25°18′N, 83°03′E); Borlaug Institute for South Asia, Samastipur, Pusa (25°57′N, 85°40′E), Bihar; and Uttar Banga Krishi Vishwavidyalaya, Coochbehar (26°19′N, 89°27′E), West Bengal, during two consecutive crop seasons 2013–14(F_5_) and 2014–15(F_6_). The F_7_ generation was evaluated only at BHU in crop season 2015–16. At each location, all RILs were planted along with their parents (YS#24 and YS#58) in two replications following a randomized complete block design (RCBD). Each line was sown in two rows of 2 m, keeping row-to-row and plant-to-plant distances of 20 and 5 cm, respectively. To ensure disease build-up and spread, two rows of the susceptible genotype Sonalika was planted after every 20th row and in alleys along with the plots. The susceptible spreader rows served as an inoculum source for epidemic development in addition to the direct inoculation on the RILs (Saxesena et al., 2017). Planting at all sites was done between the 1st and 10th of December each year to coincide with the post-anthesis with higher temperatures, conducive to disease development (Chaurasia et al., 2000). The experimental plots were fertilized at 120 kg N_2_, 60 kg P_2_O_5_, and 40 kg K_2_O per hectare. A complete dose of K_2_O and P_2_O_5_ was given at the time of sowing. The N_2_ was given in three splits of 60, 30, and 30 kg per ha at sowing, 21 days after sowing (at first irrigation), and 45 days after sowing (at second irrigation), respectively. A total of five irrigations were given to maintain sufficient moisture in the field.

### Inoculation of the Pathogen, Disease Assessment, and Estimation of Area Under the Disease Progress Curve (AUDPC)

The highly aggressive *B. sorokiniana* isolate HDBHU (NABM MAT1; NCBIJN128877) was used to create an artificial epiphytotic. The pathogen was multiplied on sorghum grains, suspended in water (10^4^ spores per ml), and sprayed in the evening at the time of flag leaf emergence as described earlier (Joshi and Chand, 2002). Disease severity (DS) was scored using a double-digit scale, first at the beginning of anthesis (GS 63) and then at the end of anthesis (GS 69) and third at the late milk stage (GS 77) (Saari and Prescott, 1975). The percentage DS was calculated as D1/9 × D2/9 × 100, where D1 is the first digit referring to the vertical progress of disease and D2 is the second digit indicating the extent of leaf area affected. The AUDPC based on DS recorded at three growth stages was calculated following Shaner and Finney (1977).

$$\text{AUDPC }=\;\sum_{i=1}^{n-1}\left[\left\{\left(Y_{i}+Y_{i+1}\right)/2\right\}\times \left(t_{\left(i+1\right)}-t_{i}\right)\right],$$

where Yi = disease level at time t_i_, t _(i+1_)−t_i_ = time (days) between two disease scores, n = number of dates when disease was recorded.

### Grouping of RILs and Construction of Bulk Samples

**FIGURE 1:**
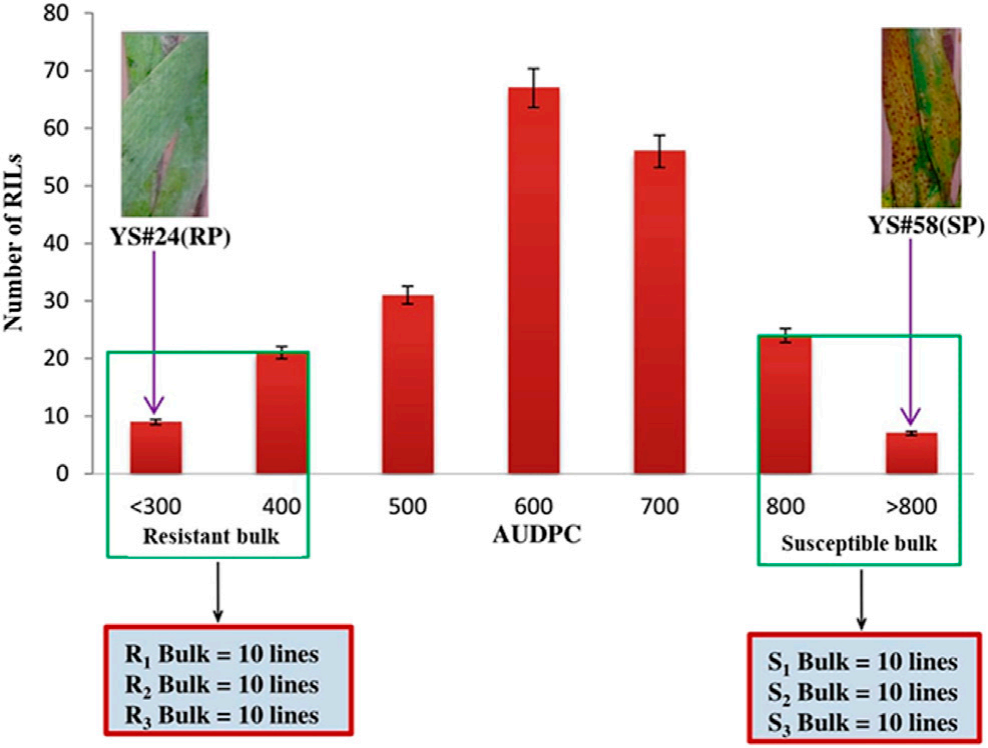
Frequency distribution of the AUDPC of the wheat RIL population and parents. Resistant parent (RP), YS#24, and susceptible parent (SP), YS#58 clearly show the phenotypic differences for SB disease severity. Resistant bulk (R1-bulk, R2-bulk, and R3-bulk) with lower AUDPC and susceptible (S1-bulk, S2-bulk, and S3-bulk) with higher AUDPC were selected from each tail.

To increase the statistical power and reduce false positives, multiple bulks were selected independently from each of the three locations in both years (Zou et al., 2016). AUDPC was analyzed from the pooled data of disease severity in all the screened environments, and three resistant bulks with lower AUDPC (R-bulk1, R-bulk2, and R-bulk3) and three susceptible bulks with higher AUDPC (S-bulk1, S-bulk2, and S-bulk3) were prepared (Figure 1). Each group bulked for resistance and susceptibility is composed of 10 RILs.

### RNA Extraction and Transcriptome Sequencing

Seeds of each RIL of the resistant and susceptible bulks (30R + 30S RILs) and their parents (YS#24 and YS#58) were grown separately in a greenhouse; seedling leaves were harvested 14 days after sowing for RNA isolation. Total RNA was extracted from the leaf tissues of each RIL using a standard TRIzol method (Catalog # 15596018) and treated with DNase I to remove residual DNA contaminants. RNA samples were purified using the RNeasy Mini Kit (Qiagen, Hilden, Germany) and the quantitative and qualitative estimation was performed using the NanoDrop 1000 (Thermo Fisher Scientific) and the Agilent Bioanalyzer 2100, respectively. An equimolar concentration of RNA from 10 resistant individuals of RILs was pooled together to make resistant bulk 1, and the same was done to prepare the other resistant and susceptible bulks. Before cDNA library preparation, we enriched the RNA samples for transcripts using the absolute mRNA Purification Kit (Agilent Technologies, Santa Clara, CA, United States). The cDNA libraries were constructed, and Illumina paired-end adapters and barcode sequences were ligated onto the cDNA fragments (Pootakham et al., 2014). The pooled libraries were sequenced at QTLomics Technologies (Bengaluru, India) using the Illumina Next Seq 500 platform (Illumina, San Diego, CA, United States) to generate 76 bp paired end (PE) sequence reads for parents and bulked samples (resistance and susceptible) in three biological replicates.

### Sequence Analysis; SNP Calling and Bulk Frequency Ratio Calculation

The data from Illumina Next Seq 500 was passed through Fast QC to check the quality of the reads (Babraham Bioinformatics - FastQC A, 2012). The ends of the reads with low quality, adaptor contamination, and low-quality regions were trimmed using the fastx-tool kit (http://hannonlab.cshl.edu/fastx_toolkit). Identification of SNPs was done; using a reference-based approach, the UniGene build63 was used as reference transcriptome (NCBI:ftp://ftp.ncbi.nhi.gov/repository/UniGene/Triticum_aestivum/Ta.seq.uniq.gz). Since UniGene represents only the genic portion, the complexity of the wheat genome was further reduced (Sidhu et al., 2015). The reads were aligned to transcript sequence build 63 using the Burrows–Wheeler Aligner (BWA) (Li and Durbin, 2009) using default parameters for PE libraries. Misaligned reads were removed from the unigene alignment and the unigenes with a high bulk frequency ratio (BFR) shortlisted. The allele frequency ratio of the resistant allele (allele of the resistant parent) in the resistant bulk compared with the resistant allele frequency in the susceptible bulk is known as the BFR. However, the genome locations of the SNPs were derived by alignment of shortlisted unigenes to the reference genome. We ran BLAST alignment and took the best hit throughout the transcript rather than the short read. The whole transcript alignment would give the best alignment to their respective genomes rather than the short read. The alignments were converted to one binary alignment/map (BAM) file per sample. On average, 80% of the RNA-Seq reads from each sample were able to align to the reference transcriptome (Pootakham et al., 2014). The SNPs for each sample were collected using the SAMtool sv0.1.18 as described by Li et al. (2009).

To identify SNPs linked to SB resistance, polymorphic markers between the parents were identified using a custom Perl script followed by BFR calculation for the bulks following Trick et al. (2012). The algorithm was implemented in Perl and R software. BFR values were calculated independently for the comparisons of three bulks (Bulk 1:S-bulk1, R-bulk1; Bulk 2: S-bulk2, R-bulk2; Bulk 3: S-bulk3, R-bulk3). BFR values and depth of each SNP for all the bulks and parents were calculated in the R program. Potential SNPs linked to the trait were selected based on SNPs with a BFR ratio of six as the minimum threshold in all three bulk replicates. The unigene that contain putative SNPs were aligned to a repeat masked reference genome (transcriptome) of wheat using BLASTN, and the best hit for each transcript was recorded. The BLAST result was segregated according to the chromosome number, and the chromosomal regions based on SNP density (twice or greater than the average SNP density) were further shortlisted. To identify SNPs that were enriched for the corresponding parental allele, BFR was calculated in the appropriate bulk, YS #24 derived SNPs for the resistant bulks, and YS#58 derived SNPs for the susceptible. Finally, to classify and prioritize the SNPs across each bulk, the frequency of the allele (SNP index) calculated at each SNP position (Takagi et al., 2013) was estimated, and then the ratio between the bulks (BFR) for each SNP was determined (Trick et al., 2012). Thus, a high BFR in the resistant parent (YS#24) derived SNP was indicative of an allele that is very frequent in the resistant bulk while depleted in the susceptible. In addition, a threshold of BFR >6 was set to select putative SNPs for the presence or absence of polymorphism between bulks for further validation as done previously in wheat (Ramirez-Gonzalez et al., 2015).

### SNP-Based Primer Designing

The tetra-primer amplification refractory mutation system-PCR (ARMS-PCR) is a simple and economical technique that produces an allele-specific reaction (Ye et al., 2001; Ruiz-Sanz et al., 2007) The principle behind this technique is that the allele-specific primer amplifies a region specific to the base present at the 3′terminus, thus making it allele-specific (Newton et al., 1989; Ye et al., 2001). The genomic region flanking 300 bp on both ends of putative SNPs was extracted and formatted using the Perl script, and the SNPs with the highest score were selected to design primers. The batch primer 3 web tools (http://probes.pw.usda.gov/batchprimer3/) were finally used to create the tetra-primer ARMS-PCR (You et al., 2008).

### DNA Isolation and PCR Conditions for Allele-Specific Primer

The genomic DNA of RILs used for BSR-Seq was extracted from the leaves of young seedlings using a cetyltrimethylammonium bromide (CTAB) protocol (Saghai-Maroof et al., 1984). The DNA pellet was vacuum dried, dissolved in DNase-free water, and stored at −20°C. A target-specific tetra-primer ARMS PCR amplification of all the resistant and susceptible bulks along with the parents was performed. The PCR was performed in a total volume of 10 μl reaction mixture, containing 30 ng DNA, 1X PCR buffer [75 mM Tris-HCl (pH9.0), 50 mM KCl, 20 mM (NH_4_)_2_SO_4_], 0.33 pmol of each outer primer (forward and reverse), 0.5 pmol of inner forward primer, 0.83 pmol of inner reverse primer, 2.5 mM MgCl_2_, 0.125 mM of each dNTPs, 1U of Taq Polymerase (3B DNA polymerase, 3B Black Bio Biotech India Ltd.). PCR reactions were performed in an Agilent SureCycler 8800 using the following program: initial denaturation at 95°C for 5 min, 38 cycles consisting of denaturation at 95°C for 1 min, annealing at 62°C for 90 s, extension at 72°C for 2 min, followed by a final extension at 72°C for 10 min. The PCR product was resolved on 3.5% agarose gel and stained with ethidium bromide to visualize.

### Functional Identification and Annotation of Transcripts

To perform the functional analysis of the transcripts, Blast2GO v 2.5 was used (Conesa et al., 2005). It is a Gene Ontology–based annotation tool and was found effective in the functional characterization of sequence data (Conesa and Götz, 2008). For functional characterization of the transcripts, we performed NCBI-BLASTX analysis using transcripts homologous to annotated proteins in the nr database with the criterion of E-value (threshold of 1e-03) and alignment size (threshold length 33). Furthermore, the transcript sequences were categorized for gene ontology (i.e., GO terms) into three groups: molecular function, biological process, and cellular component. The pathways for the selected transcripts were also delineated using the Kyoto encyclopaedia of genes and genomes **(**KEGG) database.

## Results

### Phenotyping of RIL Population and Construction of Bulk Samples

In all environments, the AUDPC of the resistant parent YS#24 was consistently and considerably higher than that of the susceptible parent YS#58 (Table 1). The mean AUDPC values of the resistant (YS#24) and susceptible parents (YS#58) were 336.29 and 820.55, respectively, over the environment (Table 1). Based on the pooled data, the RIL population for SB AUDPC revealed considerable phenotypic variation, ranging from 231.70 to 836.80. The RILs also exhibited a high coefficient of variation (CV) for AUDPC across environments, ranging from 8.80% (BHU14) to 16.92% (UBKV14), whereas the broad-sense heritability (*h* ^2^) values of AUDPC were lowest at BISA14 (0.65) and highest at BHU14 (0.87). Therefore, resistant and susceptible bulks were constituted by taking the samples independently using extreme phenotypes (resistant and susceptible) from both tails as illustrated in Figure 1.

**TABLE 1:** Disease response to SB of parents and RILs measured in different environments.

Env.	AUDPC					
	YS#24	YS#58	RILs			
	Mean ± SD	Mean ± SD	Range	Mean ± SD	*h* ^*2*^	CV
BHU14	288.90 ± 53.25	796.50 ± 81.52	142.20–906.80	583.30 ± 141.12	0.87	8.80
BHU15	401.50 ± 96.46	1062.30 ± 61.10	235.80–1016.00	701.00 ± 174.00	0.81	11.41
BHU16	360.90 ± 18.75	896.70 ± 32.89	145.20–925.40	610.00 ± 172.00	0.83	11.02
BISA14	229.80 ± 8.43	545.00 ± 39.84	219.00–588.20	424.00 ± 77.18	0.65	11.97
BISA15	330.50 ± 9.16	680.60 ± 21.38	248.40–846.90	546.39 ± 127.40	0.83	10.05
UBKV14	321.40 ± 18.58	886.00 ± 18.43	161.90–928.80	469.80 ± 173.59	0.81	16.92
UBKV15	431.80 ± 39.72	966.30 ± 39.73	192.30–1085.80	546.50 ± 178.21	0.77	16.55
Over Env	336.29 ± 68.07	820.55 ± 183.28	231.70–836.84	554.00 ± 183.60	0.92	13.61

Env, environment; AUDPC, area under disease progress curve; h^2^, broad sense heritability; SD, standard deviation; CV, coefficient of variation.

### Transcriptome Alignment and Variant Identification

**FIGURE 2:**
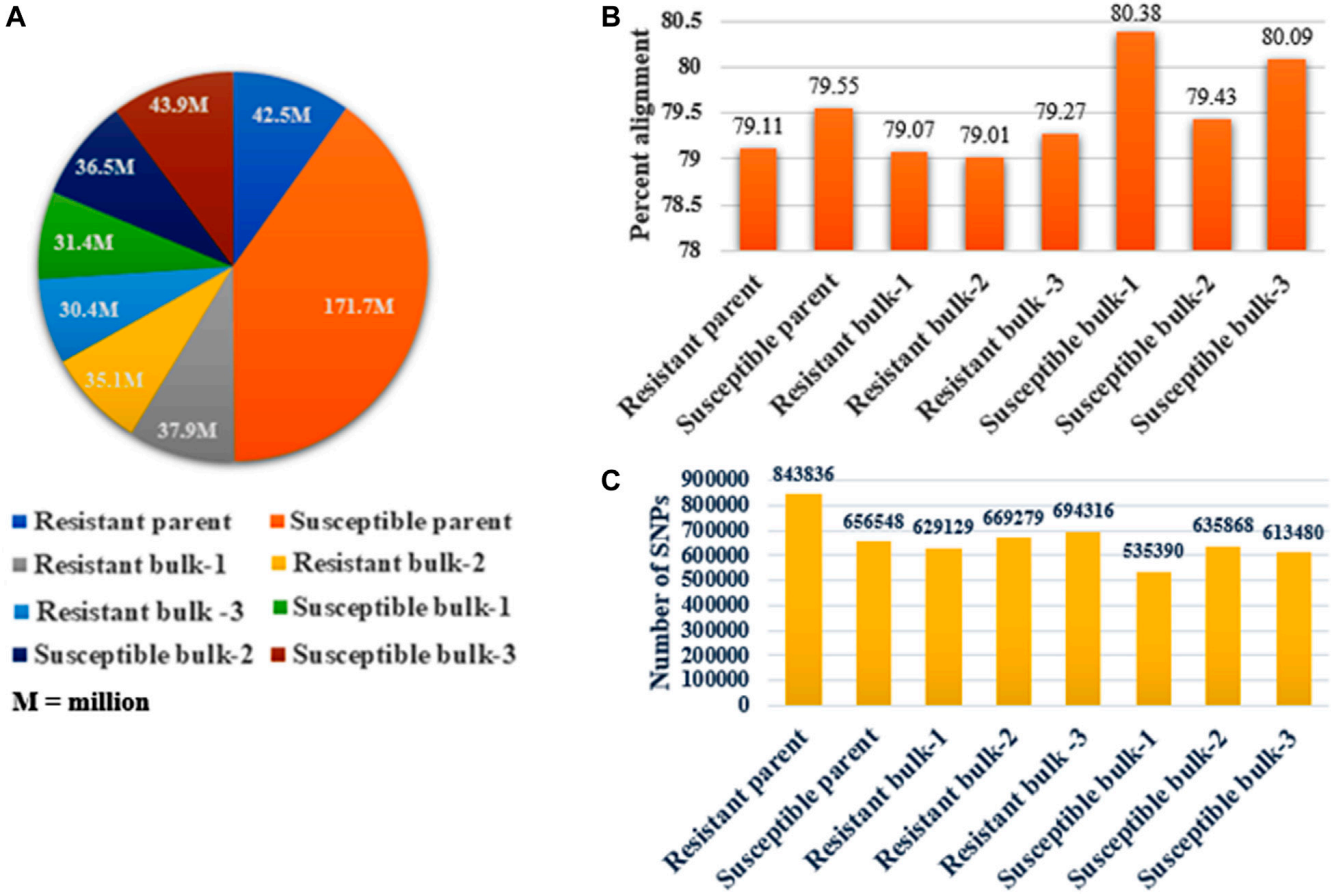
RNA-Seq data study in parents and bulk samples. **(A)** The number of raw sequence reads (76 bp/read pair) generated in resistant and susceptible parents and bulk samples. **(B)** The percentage alignment of the read to the wheat reference sequence. Susceptible bulk-1 shows the highest alignment. BWA was used to align the reads. **(C)** The total number of SNPs obtained in each sequenced sample and the maximum number of SNPs present in the resistant parent.

The bulk samples utilized for BSR-Seq produced 429.40 million raw reads, containing 32,634.40 million base pairs. The number of raw reads in the sequenced samples ranged from 30.40 million (7.08%) in the resistant bulk-3 to 171.70 million (39.99%) in the susceptible parent (Table 2). The highest mapping of reads to the reference genome was obtained for the susceptible parent (171.70 million reads), followed by susceptible bulk-3 (43.90 million reads), resistant parent (42.50 million reads), resistant bulk-1 (37.90 million reads), and susceptible bulk-2 (36.50 million reads), and the lowest values were obtained for resistant bulk-3 (30.40 million reads) (Figure 2A). All the raw sequence reads have been deposited at NCBI, and the provisional Sequence Reads Archive (SRA) identifiers were obtained under the accession codes SRR5948908.

**TABLE 2:** Number of reads generated, alignment percentage, and number of SNPs in parents, resistant bulk, and susceptible bulk of the “YS#24 × YS#58” in wheat.

Samples used for BSR-seq	Number of reads (76 bp/read pair)	% of total number of reads	Number of base pairs	Percent alignment	Number of SNPs
Resistant parent	42.50 × 10^6^	9.90	3230.0 × 10^6^	79.11%	843,836
Susceptible parent	171.70 × 10^6^	39.99	13,049.2 × 10^6^	79.55%	656,548
Resistant bulk-1	37.90 × 10^6^	8.83	2880.4 × 10^6^	79.07%	629,129
Resistant bulk-2	35.10× 10^6^	8.17	2667.6 × 10^6^	79.01%	669,279
Resistant bulk -3	30.40 × 10^6^	7.08	2310.4 × 10^6^	79.27%	694,316
Susceptible bulk-1	31.40 × 10^6^	7.31	2386.4 × 10^6^	80.38%	535,390
Susceptible bulk-2	36.50 × 10^6^	8.50	2774.0 × 10^6^	79.43%	635,868
Susceptible bulk-3	43.90 × 10^6^	10.22	3336.4 × 10^6^	80.09%	613,480
Total	429.40× 10^6^	100	32,634.4 × 10^6^	—	5277846

Each sample yielded an average of 80% high-quality (free of adaptor contamination and low-quality areas) RNA-Seq reads that were matched to the wheat reference transcriptome. Susceptible bulk-1 had the highest percentage alignment (80.38% with 535390 SNPs), followed by susceptible bulk-3 (80.09% with 613480 SNPs), and resistant bulk-1 had the lowest percentage alignment (79.01% with 669279 SNPs) (Table 2). The resistant parent had the highest number of SNPs (843836), whereas the minimum number (535390) was in the susceptible bulk-1 with the highest percentage of alignment **(** Figures 2B,C **)**.

### BFR and Polymorphic SNPs Associated With SB Resistance

A total of 1,379,122 SNPs were identified on 91,855 transcripts of wheat samples used in the present investigation (Table 3). The putative SNPs linked to SB were selected based on SNPs with BFR >6 in all three bulk samples, i.e., 3666 SNPs present on 1837 transcripts in Bulk-1 (S-bulk1: R-bulk1), 3635 SNPs on 2056 transcripts in Bulk-2 (S-bulk2: R-bulk2), and 2130 SNPs on 1443 transcripts in Bulk-3 (S-bulk3: R-bulk3). A total of 7860 SNPs with >6 BFR were found across all bulks (Bulk-1: Bulk-2: Bulk-3) on 3950 transcripts. Out of 7860 SNPs, only 1055 SNPs were present on 506 transcripts detected as homozygous and polymorphic between parents (Table 3). The transcripts carrying homozygous and polymorphic SNPs with other details are listed in Supplementary Table S1.

**TABLE 3:** Number of transcripts containing SNPs having BFR >6 in bulked samples.

Description of samples	SNPs count	Number of transcripts
Raw SNP count	1379122	91855
Bulk 1 BFR >6	3666	1837
Bulk 2 BFR >6	3635	2056
Bulk 3 BFR >6	2130	1443
Bulk total BFR >6	7860	3950
Bulk total with parental polymorphism	1055	506

### Distribution of Polymorphic SNPs for SB in Wheat Genome

**FIGURE 3:**
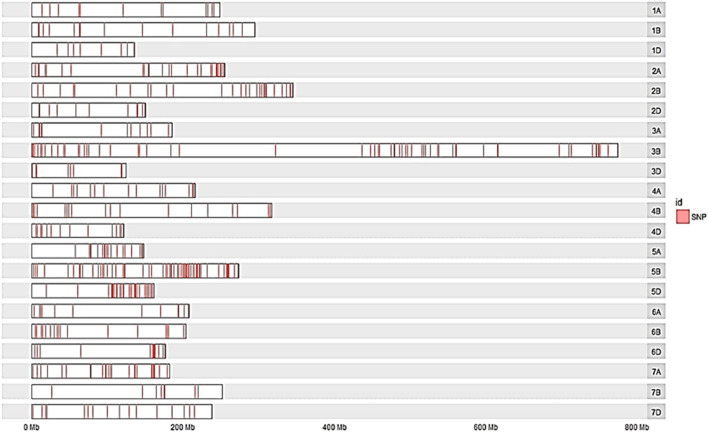
Bar plot showing the densities of polymorphic SNPs marker with bulk frequency ratio greater than 6 (BFR >6) on the 21 wheat chromosomes of the cross “YS#24 × YS#58” RIL population. The locations of the SNPs were determined by the best alignment to the wheat genome with the transcript containing the SNPs.

**FIGURE 4:**
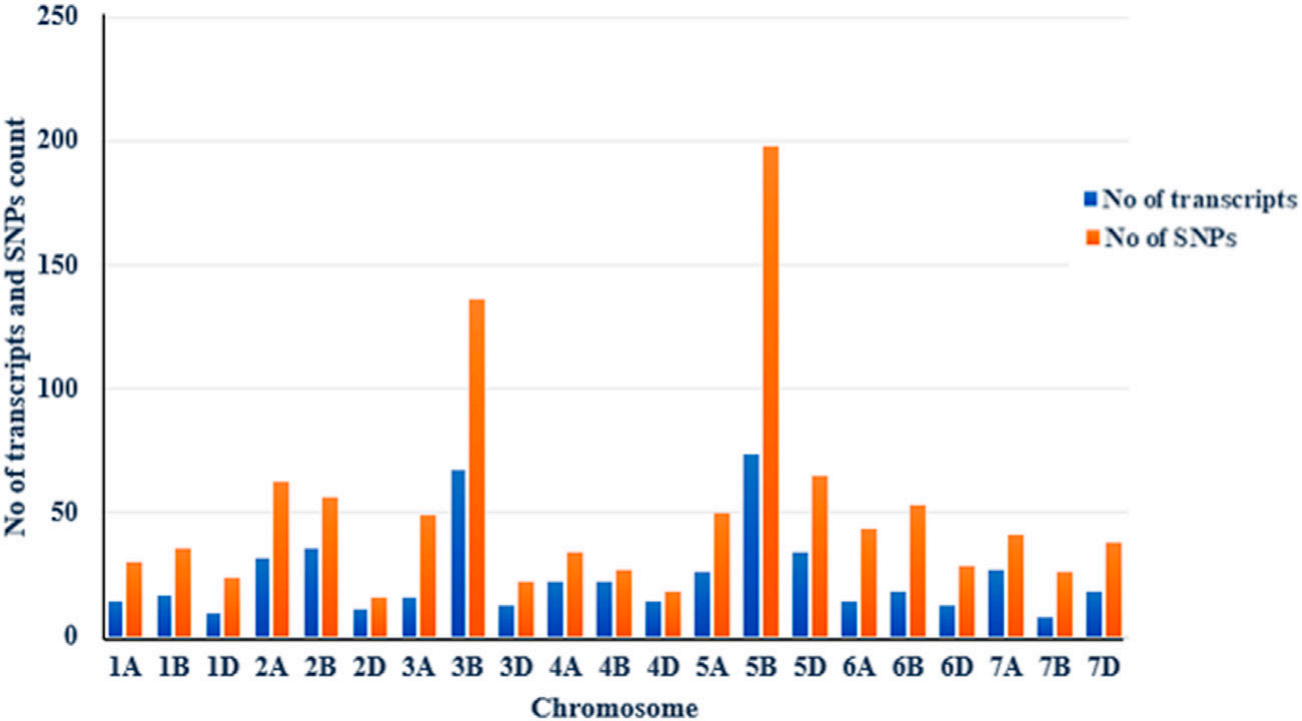
Chromosomal distribution of SB-associated transcripts (blue bar) and polymorphic SNPs (orange bar) on wheat chromosomes with a bulk frequency ratio greater than 6. The figure shows that the 5B chromosome has the maximum number of SNPs, followed by the 3B.

The distribution of trait-linked SNP markers in the A, B, and D genomes for each homoeologous group and percentage of the total number of SNPs is given in Table 4. The 1055 polymorphic SNP markers, bar plotted on 21 wheat chromosomes, formed seven unevenly distributed homoeologous groups (Figure 3). The highest number of SNP markers were identified in the B genome (532 SNP; 50.42%), followed by A (311 SNP; 29.46%) and D (212 SNP; 20.09%). The number of SNPs per linkage group ranged from 16 (2D) to 198 (5B) (Figure 4). The homologous group 5 had the highest markers (313 SNP, 29.67%), followed by group 3 (207 SNP, 19.60%), and group 4 had the lowest (79 SNP, 7.49%) (Table 4).

**TABLE 4:** Distribution of polymorphic SNPs markers of SB with BFR >6 across the A, B, and D genomes of wheat.

Chromosome	Number of SNPs	Number of transcripts	% of total number of SNPs	Number of SNPs in homoeologous group	Percentage
1A	30	14	2.84	90	8.53
1B	36	17	3.41		
1D	24	10	2.27		
2A	63	32	5.97	135	12.79
2B	56	36	5.30		
2D	16	11	1.52		
3A	49	16	4.64	207	19.62
3B	136	67	12.89		
3D	22	13	2.09		
4A	34	22	3.22	79	7.49
4B	27	22	2.56		
4D	18	14	1.70		
5A	50	26	4.74	313	29.67
5B	198	74	18.77		
5D	65	34	6.16		
6A	44	14	4.17	126	11.94
6B	53	18	5.02		
6D	29	13	2.75		
7A	41	27	3.88	105	9.95
7B	26	8	2.46		
7D	38	18	3.60		
Average	50.23	24.09	4.76	150.71	14.29
Total	1055	506	100	1055	100

### Analysis of Polymorphic SNPs on Chromosomes 3B and 5B

**FIGURE 5:**
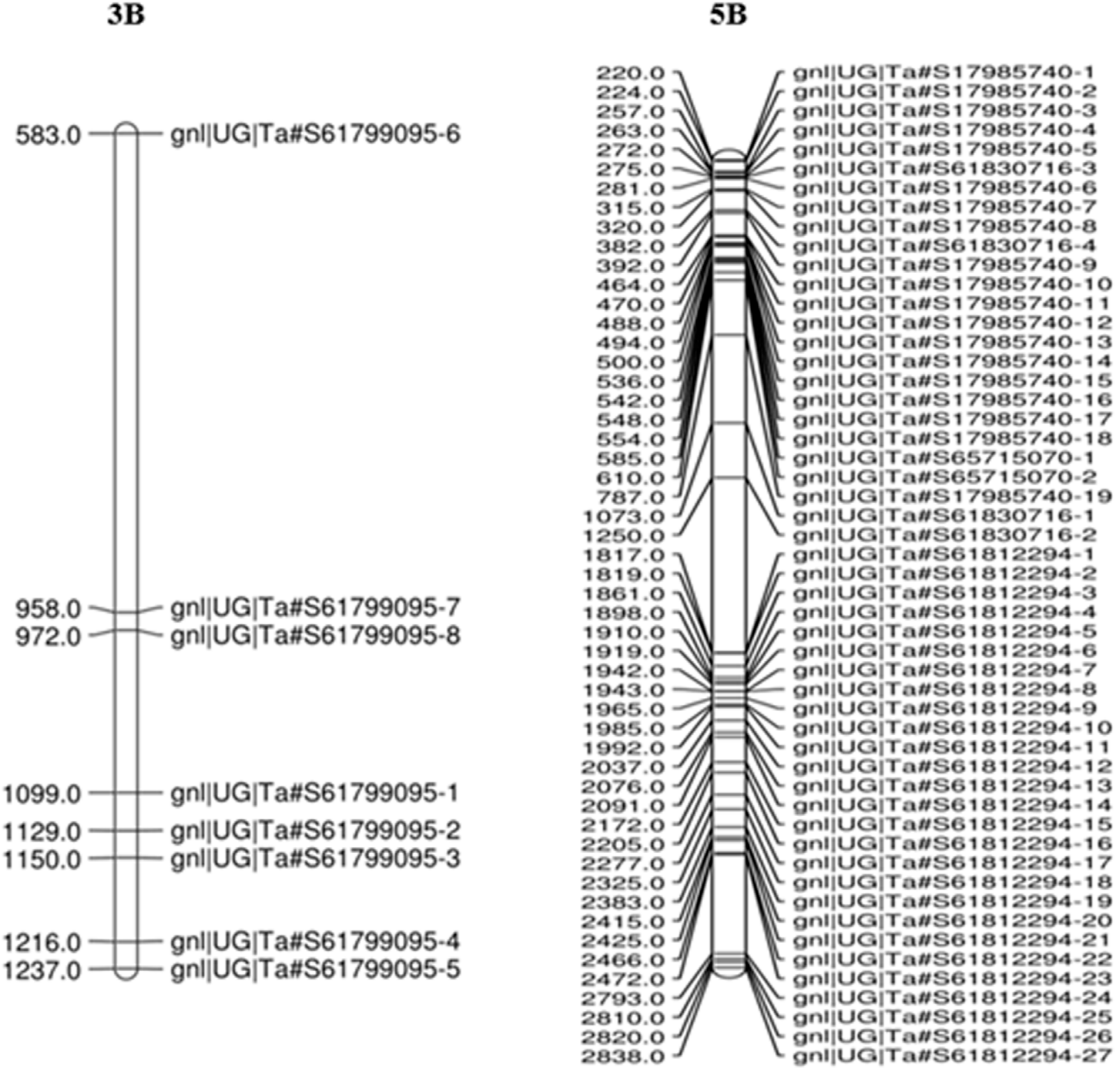
The distribution of 60 polymorphic SNPs linked to SB resistance on the wheat chromosome derived from the RIL population. The eight SNPs present on 3B and 52 SNPs on 5B chromosomes indicate their relative position.

The maximum number of polymorphic SNP with BFR >6 was found on chromosome 5B (198 SNP), followed by 3B (136 SNP) (Figure 4). Thus, a total of 334 SNPs of 3B and 5B chromosomes were present on 142 transcripts, out of which 60 SNPs were found only on five transcripts. Out of these five, one transcript of the 3B chromosome had eight SNPs, and four transcripts of the 5B chromosome had 52 SNPs (Table 5). Among the four transcripts of 5B, a maximum of 27 SNPs were present on transcript gnl/UG/Ta#S61812294, followed by transcripts gnl/UG/Ta#S17985740 (19 SNPs) and gnl/UG/Ta#S61830716 (4 SNPs), while transcript gnl/UG/Ta#S65715070 had the lowest number (2 SNPs) (Table 5). The chromosomal distribution of 60 polymorphic SNP present on five different transcripts of the 3B and 5B chromosomes associated with SB resistance is shown in Figure 5, which indicates their relative position.

**TABLE 5:** Transcripts of 3B and 5B chromosomes having SNPs markers of SB resistance with BFR >6.

Chromosome	No. of transcripts	Total number of SNPs	Transcript ID	Number of SNPs
3B	1	8	gnl/UG/Ta#S61799095	8
5B	4	52	gnl/UG/Ta#S17985740	19
			gnl/UG/Ta#S61812294	27
			gnl/UG/Ta#S61830716	4
			gnl/UG/Ta#S65715070	2

### Development of an Assay From Allele-Specific Primers in Bulk Samples

**FIGURE 6:**
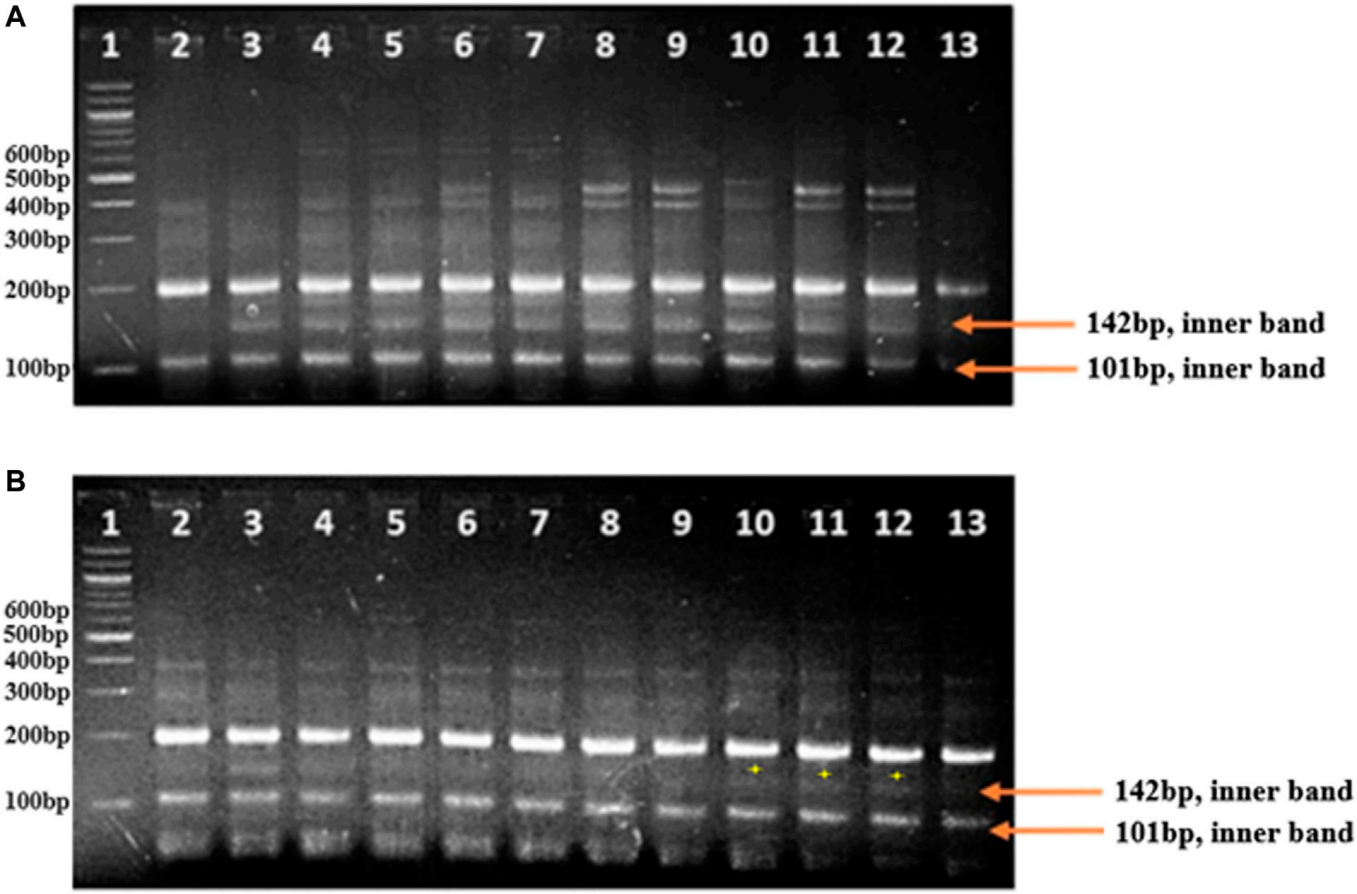
**(A)** Lane 1: 100 bp ladder, Lane 2: susceptible parent, Lane 3: resistant parent, Lane 4–13: resistant lines. The resistant lines amplify the inner allele-specific primer region with a product size of 142 bp, which is absent in the susceptible parent. **(B)** Lane 1: 100bp ladder, Lane 2: susceptible parent, Lane 3: resistant parent, Lane 4–13: susceptible lines. The inner allele-specific primer region amplifies in the resistant parent at 142 bp and is absent in the susceptible lines except for lanes 10–12.

In this study, allele-specific tetra-primer ARMS PCR primers were designed to develop an assay for SB resistance. The SNPs on transcript gnl/UG/Ta#S61799095 of 3B chromosomes and transcript gnl/UG/Ta#S61830716 and gnl/UG/Ta#S17985740 of chromosome 5B were used to design the tetra-primers for ARMS PCR (Supplementary Table S2). A primer (*Ta_S61830716_1262_3*) from chromosome 5B amplified 142 bp fragments in both the resistant parent and resistant bulk-1, whereas a 142 bp fragment was not amplified in the susceptible parent, but in a few samples of susceptible bulk-1, a faint band appeared in lane 10–12 (Figures 6A,B). The primer (Ta_S61830716_1262_3) showed clear differentiation between resistant and susceptible genotypes.

### Pathway Enrichment Analysis Using GO and KEGG

The transcript of 3B chromosomes (gnl/UG/Ta#S61799095) shared homology with acetyl-CoA acetyltransferase, whereas two transcripts of 5B (gnl/UG/Ta#S17985740 and gnl/UG/Ta#S61830716) shared homology with proteinase/protease and the remaining two with phospholipase C1 and exohydrolase proteins (Supplementary Table S3). A total of 346 GO terms were identified for the selected transcripts and further categorized into molecular function, biological process, and cellular component (Supplementary Figure S2). The maximum number of GO terms fall into the molecular function category (231 terms), which were further grouped into hydrolase activity, transferase activity, peptidase activity, and catalytic activity. There were 70 terms associated with the biological process, which fall into the category of protein catabolic process, carbohydrate metabolic process, metabolic process, phospholipid catabolic process, and fatty acid beta-oxidation (Supplementary Figure S2). A few terms were associated with cellular components, viz., peroxisome, membrane, an integral component of the membrane, lysosome, and extracellular space. Furthermore, as shown in Supplementary Figure S3, the most highly identified pathways in the study were fatty acid metabolism, valine, leucine, isoleucine metabolism, and benzoate degradation.

## Discussion

In the 21st century, wheat is placed as one of the world’s most productive and essential crops (Curtis and Halford, 2014), contributing significantly to global food security by providing nutrition for 35% of the world population (FAO, 2018; Tomar et al., 2021). The continuous threat of SB fungus in major wheat-growing areas of the world results in a significant yield loss that affects future food security. Wheat cultivars with host resistance are employed to minimize crop yield loss, which is the most effective and economical way to manage SB (Gupta et al., 2018). However, the conventional breeding approaches to develop cultivars with disease resistance have certain limitations due to the genetic complexity of wheat (Zhang et al., 2020). Several molecular breeding tools and techniques have been developed to study the genetics and genomics of plants having simple and complex genomes. For a significant period, researchers primarily relied on SSR markers, but their sparse distribution across the genome rendered them less ideal for a large-scale genotyping assay (Pootakham et al., 2014). Recently, SNP markers have become increasingly popular in molecular genetics and breeding studies due to their abundance. However, SNP discovery in organisms with highly repetitive DNA and polyploid nature, such as wheat, remains difficult (Ganal et al., 2009; Trick et al., 2012). The SNP discovery *via* transcriptome sequencing is an attractive strategy to reduce genome complexity in wheat (Westermann et al., 2012; Edae and Rouse, 2019). To reduce the complexity of the data, we focused on sequencing the wheat transcriptome using RNA-Seq instead of genomic DNA.

Due to the introduction of next-generation sequencing, combining BSA and RNA-Seq (BSR-Seq) reduces the cost remarkably when repetitive sequences are enriched in the genome and enabled a rapid and detailed understanding of a near-complete set of transcripts and SNPs linked to the trait (Garg et al., 2011; Zou et al., 2016). In this context, the RIL population was used for making bulks with extreme phenotypes for SB disease. The RIL population’s AUDPC showed a continuous distribution across environments, indicating that SB resistance components behave like quantitative traits (Figure 1) as quantitative traits generally show a continuous phenotypic distribution. The estimated broad-sense heritability for AUDPC was high (0.92) over the environments, indicating good reproducibility of the phenotypic data (Table 1). The high heritability over environments revealed the genetic control of AUDPC. The numbers of bulks for pooling were selected in multiples (in replicate) independently from each of the two tails by following Navabi et al. (2009) for small- to moderate-sized populations; the optimum tail size should be 20%–30% of the entire population. The replicated number of bulks for pooling provides high accuracy in SNP predictions by reducing false positives, increasing the likelihood of obtaining reliable markers by many orders of magnitude. The parents were used to define the SNPs rather than to make quantitative estimates; therefore, replications to detect SNPs were omitted (Ramirez-Gonzalez et al., 2015). In diploid crops of the medium genome, approximately 57–65 million reads were generated and successfully achieved higher (>90%) genome coverage in cucumber (Lu et al., 2014) and pigeon pea (Singh et al., 2016). In this study, 429.40 million reads were generated for the bulk samples; these excess reads provided better coverage to the genome. In a bulked sample of groundnut (allotetraploid) for rust and late leaf spot resistance, 423.70 million reads were generated by Pandey et al. (2017). Because bread wheat is hexaploid, having three subgenomes (A, B, and D), more sequence data should be generated than the other diploid species, to achieve maximum genome coverage and read depth. Hence, the generated sequencing data with maximum genome coverage and read depth allowed for detailed sequence analysis. The alignment of reads to the reference transcriptome revealed that the maximum number of SNPs were present in the resistant parent, whereas the minimum was in the susceptible bulk-1 with the highest percentage of alignment. The higher percentage alignment indicated that the sample was closer to the reference being aligned. In the present study, a total of 1,379,122 genome-wide, high-quality SNPs were identified in parents and resistant and susceptible bulks of wheat after sequence alignment of filtered reads, and only 1055 SNPs were detected as polymorphic between the parents using BFR >6, i.e., associated with SB resistance. Among the 1055 SNPs, 198 (18.77%) and 136 (12.89%) were mapped on chromosomes 5B and 3B, suggesting the SB resistance gene might be located on chromosome 5B or 3B. Among the three genomes, there were polymorphic SNPs present in the B genome (50.42%) compared with A (29.46%) and D (20.09%) (Table 4). A similar result was reported by Kumar et al. (2009) in wheat when parents were screened with SSR markers. However, the density of polymorphic SNPs throughout the B genome was not uniform; only the 5B and 3B chromosomes were found saturated considerably with SNP markers having a magnitude of BFR >6. Kumar et al. (2009, 2010) also report two QTLs for SB resistance on the 5B chromosome in two different populations that explained around 38.62% and 10.70% of the phenotypic variation, respectively.

In the recent past, three major SB resistance genes, *Sb2* (Kumar et al., 2015), *Sb3* (Lu et al., 2016), and *Sb4* (Zhang et al., 2020), were identified in the B genome of wheat. Thus, the previous (SSR, SNP) and present SNP studies indicate the effectiveness of the B genome for SB resistance, especially 5, 4, and 3B. The average number of SNPs mapped to each linkage group was 50.23, whereas the highest number of markers was mapped to 5B. In this study, the D genome was found less saturated compared with A and B because a lesser number of polymorphic SNP markers (>6 BFR) was identified on it, which is commensurate with microsatellite markers reported in wheat (Ganal and Röder, 2007). However, the first resistance gene (*Sb1*) for SB was reported on the 7D chromosome by Lillemo et al. (2013); as a result, the importance of the D genome on a saturation basis cannot be overstated. It appears to indicate that, despite the lesser number of SNPs identified on the D genome, we should pick only those SNPs having the highest magnitude of BFR. Because the higher the BFR, the more likely the SNP is genetically linked to the *R*-gene, the putative SNPs with enriched BFR can then be converted into high-throughput SNP assays and genotyped across the individuals that were used to assemble the bulk (Ramirez-Gonzalez et al., 2015). Finally, the key issue is to move from *in silico* SNPs into a high-throughput SNP assay with allele-specific markers that can score on agarose gel electrophoresis. The allelic-specific primer *XTaSb_S61830716_1262_3*, designed from SNPs present on transcript id *Ta_S61830716*, was found polymorphic in parents and validated in each line of resistant bulk-1, but only in 70% lines of the susceptible bulk-1 and was named as a marker of SB resistance. The marker *XTaSb_S61830716_1262_3* is characterized by a few individuals of susceptible bulks as resistant but is otherwise susceptible phenotypically, limiting the efficiency of the marker.

Because plant immunity is regulated by the expression of pathogenesis-related genes (PRs), transcription is de-repressed by pathogen-induced signals. The studied transcripts here are shown to have homology with pathogenesis-related genes. The transcript gnl/UG/Ta#S61799095 of 3B identified in this study showed homology with acetyl-CoA acetyltransferase, which is conferred for its pathogenesis-related activity in rice (Zhong et al., 2015). It is a vital starting molecule for the biosynthesis of various metabolites. The transcripts of 5B (gnl/UG/Ta#S17985740 and gnl/UG/Ta#S61830716) also show homology to cysteine proteinase and proteases. Because different families of proteases manage the extracellular defense, which contributes to effector-triggered immunity (ETI), others help in the induction of microbe-associated molecular pattern–triggered immunity. A few proteases/proteinases are associated with systemic acquired resistance and the establishment of induced systemic resistance (Kim and Hwang, 2015; Balakireva and Zamyatnin, 2018). The other transcripts of 5B (gnl/UG/Ta#S61812294 and gnl/UG/Ta#S65715070) were found homologous to phospholipase and exohydrolase. It is reported that phospholipase affects the translocation of nonexpressor pathogenesis-related (NPR) proteins to the nucleus in *Arabidopsis thaliana*. The structural changes and localization of this protein in plant cells are responsible for the plant defense signaling (Kinkema et al., 2000; Janda et al., 2015). The major pathways identified in the study are fatty acid degradation and valine, leucine, and isoleucine degradation, along with other pathways. Fatty acid degradation is the process by which fatty acids break down into their metabolites, which finally generates acetyl-CoA, the entry molecule for the citric acid cycle. In the case of *Brassica napus*, when infected with the pathogen, it significantly enriched in fatty acid oxidation activities in the upregulated gene sets on both susceptible and resistant lines (Chittem et al., 2020). Thus, the study of five wheat transcripts shows that they are closely related to genes involved in pathogenesis and metabolism, suggesting their prominent role in the plant defense mechanism.

## Conclusion

In this study, BSA combined with RNA-Seq (BSR-Seq) appears to be useful for SNP discovery in bread wheat for SB resistance, later used to develop a new marker assay. The marker *XTaSb_S61830716_1262_3* could be productive in screening wheat germplasm for SB resistance. In future projects, the SNPs discovered for SB resistance across the wheat genome will be visualized by converting them into Kompetitive Allele Specific PCR (KASP) markers for establishing a high-throughput genotyping platform, useful for MAS of the target genes. The newly developed SNPs marker could also be converted to a qPCR-based assay for large-scale application in crop improvement and study of the molecular biology of SB resistance.

## Data Availability

The data sets presented in this study can be found in online repositories. The names of the repository/repositories and accession number(s) can be found in the article/Supplementary Material.

## References

[B1] Acharya K. Dutta A. K. Pradhan P. (2011). Bipolaris Sorokiniana (Sacc.) Shoem. The Most Destructive Wheat Fungal Pathogen in the Warmer Areas. Aust. J. Crop Sci. 5, 1064–1071.

[B2] Babraham Bioinformatics - FastQC A (2012). Quality Control Tool for High Throughput Sequence Data. Available at: https://www.bioinformatics.babraham.ac.uk/projects/fastqc/ (Accessed January 18, 2022).

[B3] Balakireva A. Zamyatnin A. (2018). Indispensable Role of Proteases in Plant Innate Immunity. Ijms 19, 629. 10.3390/IJMS19020629 PMC5855851 29473858

[B4] Castro A. C. Fleitas M. C. Schierenbeck M. Gerard G. S. Simón M. R. (2018). Evaluation of Different Fungicides and Nitrogen Rates on Grain Yield and Bread-Making Quality in Wheat Affected by Septoria Tritici Blotch and Yellow Spot. J. Cereal Sci. 83, 49–57. 10.1016/j.jcs.2018.07.014

[B5] Chaurasia S. Chand R. Joshi A. K. (2000). (PDF) Relative dominance of Alternaria triticina Pras. et Prab. and Bipolaris sorokiniana (Sacc.) Shoemaker in different growth stages of wheat (*T. aestivum* L.). Plant Dis. Prot. 107, 176–181 . https://www.researchgate.net/publication/286885969_Relative_dominance_of_Alternaria_triticina_Pras_et_Prab_and_Bipolaris_sorokiniana_Sacc_Shoemaker_in_different_growth_stages_of_wheat_T_aestivum_L (Accessed January 18, 2022). Available at

[B6] Chittem K. Yajima W. R. Goswami R. S. del Río Mendoza L. E. (2020). Transcriptome Analysis of the Plant Pathogen Sclerotinia sclerotiorum Interaction with Resistant and Susceptible Canola (Brassica Napus) Lines. PLoS ONE 15, e0229844. 10.1371/JOURNAL.PONE.0229844 32160211 PMC7065775

[B7] Conesa A. Götz S. (2008). Blast2GO: A Comprehensive Suite for Functional Analysis in Plant Genomics. Int. J. Plant Genomics 2008, 1–12. 10.1155/2008/619832 PMC2375974 18483572

[B8] Conesa A. Götz S. García-Gómez J. M. Terol J. Talón M. Robles M. (2005). Blast2GO: a Universal Tool for Annotation, Visualization and Analysis in Functional Genomics Research. Bioinformatics 21, 3674–3676. 10.1093/BIOINFORMATICS/BTI610 16081474

[B9] Curtis T. Halford N. G. (2014). Food Security: the challenge of Increasing Wheat Yield and the Importance of Not Compromising Food Safety. Ann. Appl. Biol. 164, 354–372. 10.1111/AAB.12108 25540461 PMC4240735

[B10] Dubin H. J. Van Ginkel M. (1991). “The Status of Wheat Diseases and Disease Research in Warmer Areas,” in Wheat for the Nontraditional Warmer Areas: A Proceedings of the International Conference (CIMMYT, 125–145. Available at: http://site.cabdirect.org/cabdirect/abstract/19932328160 .

[B11] Duveiller E. M. Sharma R. C. (2009). Genetic Improvement and Crop Management Strategies to Minimize Yield Losses in Warm Non-traditional Wheat Growing Areas Due to Spot Blotch PathogenCochliobolus Sativus. J. Phytopathol. 157, 521–534. 10.1111/J.1439-0434.2008.01534.X

[B12] Edae E. A. Rouse M. N. (2019). Bulked Segregant Analysis RNA-Seq (BSR-Seq) Validated a Stem Resistance Locus in Aegilops Umbellulata, a Wild Relative of Wheat. PLoS One 14, e0215492–19. 10.1371/journal.pone.0215492 31539379 PMC6754143

[B13] Fao (2018). World Food and Agriculture - Statistical Pocketbook 2018. World Food Agric. - Stat. Pocketb. 2018, 1. 10.4060/CA1796EN

[B14] Ganal M. W. Altmann T. Röder M. S. (2009). SNP Identification in Crop Plants. Curr. Opin. Plant Biol. 12, 211–217. 10.1016/j.pbi.2008.12.009 19186095

[B15] Ganal M. W. Röder M. S. (2007). Microsatellite and SNP Markers in Wheat Breeding. Genomics-Assisted Crop Improv. 2, 1–24. 10.1007/978-1-4020-6297-1

[B16] Garg R. Patel R. K. Tyagi A. K. Jain M. (2011). De Novo assembly of Chickpea Transcriptome Using Short Reads for Gene Discovery and Marker Identification. DNA Res. 18, 53–63. 10.1093/dnares/dsq028 21217129 PMC3041503

[B17] Grabherr M. G. Haas B. J. Yassour M. Levin J. Z. Thompson D. A. Amit I. (2011). Full-length Transcriptome Assembly from RNA-Seq Data without a Reference Genome. Nat. Biotechnol. 29, 644–652. 10.1038/nbt.1883 21572440 PMC3571712

[B18] Gupta P. K. Chand R. Vasistha N. K. Pandey S. P. Kumar U. Mishra V. K. (2018). Spot Blotch Disease of Wheat: the Current Status of Research on Genetics and Breeding. Plant Pathol. 67, 508–531. 10.1111/PPA.12781

[B19] Hiebert C. W. McCallum B. D. Thomas J. B. (2014). Lr70, a New Gene for Leaf Rust Resistance Mapped in Common Wheat Accession KU3198. Theor. Appl. Genet. 127, 2005–2009. 10.1007/s00122-014-2356-1 25112203

[B20] Jaganathan D. Bohra A. Thudi M. Varshney R. K. (2020). Fine Mapping and Gene Cloning in the post-NGS Era: Advances and Prospects. Theor. Appl. Genet. 133, 1791–1810. 10.1007/s00122-020-03560-w 32040676 PMC7214393

[B21] Janda M. Å aÅ¡ek V. r. ChmelaÅ™ovÃ¡ H. Andrejch J. NovÃ¡kovÃ¡ M. HajÅ¡lovÃ¡ J. (2015). Phospholipase D Affects Translocation of NPR1 to the Nucleus in *Arabidopsis thaliana* . Front. Plant Sci. 6, 59. 10.3389/fpls.2015.00059 25741350 PMC4332306

[B22] Joshi A. K. Chand R. (2002). Variation and Inheritance of Leaf Angle, and its Association with Spot Blotch (Bipolaris Sorokiniana) Severity in Wheat (*Triticum aestivum*). Euphytica 124, 283–291. 10.1023/a:1015773404694

[B23] Joshi A. K. Kumar S. Chand R. Ortiz-Ferrara G. (2004). Inheritance of Resistance to Spot Blotch Caused by Bipolaris Sorokiniana in spring Wheat. Plant Breed. 123, 213–219. 10.1111/J.1439-0523.2004.00954.X

[B24] Joshi A. K. Mishra B. Chatrath R. Ortiz Ferrara G. Singh R. P. (2007a). Wheat Improvement in India: Present Status, Emerging Challenges and Future Prospects. Euphytica 157, 431–446. 10.1007/S10681-007-9385-7

[B25] Joshi A. K. Ortiz-Ferrara G. Crossa J. Singh G. Alvarado G. Bhatta M. R. (2007b). Associations of Environments in South Asia Based on Spot Blotch Disease of Wheat Caused by Cochliobolus Sativus. Crop Sci. 47, 1071–1081. 10.2135/CROPSCI2006.07.0477

[B26] Kim N. H. Hwang B. K. (2015). Pepper Pathogenesis-Related Protein 4c Is a Plasma Membrane-Localized Cysteine Protease Inhibitor that Is Required for Plant Cell Death and Defense Signaling. Plant J. 81, 81–94. 10.1111/TPJ.12709 25335438

[B27] Kinkema M. Fan W. Dong X. (2000). Nuclear Localization of NPR1 Is Required for Activation of PR Gene Expression. Plant Cell 12, 2339–2350. 10.1105/TPC.12.12.2339 11148282 PMC102222

[B28] Klein H. Xiao Y. Conklin P. A. Govindarajulu R. Kelly J. A. Scanlon M. J. (2018). Bulked-Segregant Analysis Coupled to Whole Genome Sequencing (BSA-Seq) for Rapid Gene Cloning in Maize. G3 (Bethesda). 8, 3583–3592. 10.1534/G3.118.200499 30194092 PMC6222591

[B29] Kumar S. Röder M. S. Tripathi S. B. Kumar S. Chand R. Joshi A. K. (2015). Mendelization and fine Mapping of a Bread Wheat Spot Blotch Disease Resistance QTL. Mol. Breed. 35, 1. 10.1007/s11032-015-0411-5

[B30] Kumar U. Joshi A. K. Kumar S. Chand R. Röder M. S. (2009). Mapping of Resistance to Spot Blotch Disease Caused by Bipolaris Sorokiniana in spring Wheat. Theor. Appl. Genet. 118, 783–792. 10.1007/s00122-008-0938-5 19066842

[B31] Kumar U. Joshi A. K. Kumar S. Chand R. Röder M. S. (2010). Quantitative Trait Loci for Resistance to Spot Blotch Caused by Bipolaris Sorokiniana in Wheat (*T. aestivum* L.) Lines 'Ning 8201' and 'Chirya 3'. Mol. Breed. 26, 477–491. 10.1007/S11032-009-9388-2

[B32] Li H. Durbin R. (2009). Fast and Accurate Short Read Alignment with Burrows-Wheeler Transform. Bioinformatics 25, 1754–1760. 10.1093/bioinformatics/btp324 19451168 PMC2705234

[B33] Li H. Handsaker B. Wysoker A. Fennell T. Ruan J. Homer N. (2009). The Sequence Alignment/Map Format and SAMtools. Bioinformatics 25, 2078–2079. 10.1093/bioinformatics/btp352 19505943 PMC2723002

[B34] Lillemo M. Joshi A. K. Prasad R. Chand R. Singh R. P. (2013). QTL for Spot Blotch Resistance in Bread Wheat Line Saar Co-locate to the Biotrophic Disease Resistance Loci Lr34 and Lr46. Theor. Appl. Genet. 126, 711–719. 10.1007/s00122-012-2012-6 23139144

[B35] Liu S. Yeh C.-T. Tang H. M. Nettleton D. Schnable P. S. (2012). Gene Mapping via Bulked Segregant RNA-Seq (BSR-Seq). PLoS One 7, e36406. 10.1371/JOURNAL.PONE.0036406 22586469 PMC3346754

[B36] Lu H. Lin T. Klein J. Wang S. Qi J. Zhou Q. (2014). QTL-seq Identifies an Early Flowering QTL Located Near Flowering Locus T in Cucumber. Theor. Appl. Genet. 127, 1491–1499. 10.1007/s00122-014-2313-z 24845123

[B37] Lu P. Liang Y. Li D. Wang Z. Li W. Wang G. (2016). Fine Genetic Mapping of Spot Blotch Resistance Gene Sb3 in Wheat (*Triticum aestivum*). Theor. Appl. Genet. 129, 577–589. 10.1007/s00122-015-2649-z 26747045

[B38] Mondal S. Rutkoski J. E. Velu G. Singh P. K. Crespo-Herrera L. A. Guzmán C. (2016). Harnessing Diversity in Wheat to Enhance Grain Yield, Climate Resilience, Disease and Insect Pest Resistance and Nutrition through Conventional and Modern Breeding Approaches. Front. Plant Sci. 7, 991. 10.3389/fpls.2016.00991 27458472 PMC4933717

[B39] Monyo E. Osiru M. Kadyampakeni D. Mponda O. Chinyamunyamu B. (2009). “Improving Food Security and Nutrition in Malawi and Tanzania through Research on Edible Legumes,” in Proceedings of Stakeholder Workshops on Groundnut Production in Malawi and Tanzania, Mtwara, Tanzania, 1-2 March 2007.

[B40] Navabi A. Mather D. E. Bernier J. Spaner D. M. Atlin G. N. (2009). QTL Detection with Bidirectional and Unidirectional Selective Genotyping: Marker-Based and Trait-Based Analyses. Theor. Appl. Genet. 118, 347–358. 10.1007/S00122-008-0904-2 18854970

[B41] Newton C. R. Graham A. Heptinstall L. E. Powell S. J. Summers C. Kalsheker N. (1989). Analysis of Any point Mutation in DNA. The Amplification Refractory Mutation System (ARMS). Nucl. Acids Res. 17, 2503–2516. 10.1093/NAR/17.7.2503 2785681 PMC317639

[B42] O’Neil S. T. Emrich S. J. (2013). Assessing De Novo Transcriptome Assembly Metrics for Consistency and Utility. BMC Genomics 14, 465. 10.1186/1471-2164-14-465 23837739 PMC3733778

[B43] Pandey A. K. Mishra V. K. Chand R. Navathe S. Budhlakoti N. Srinivasa J. (2021). Crosses with Spelt Improve Tolerance of South Asian spring Wheat to Spot Blotch, Terminal Heat Stress, and Their Combination. Sci. Rep. 11, 1–12. 10.1038/s41598-021-85238-x 33727567 PMC7966735

[B44] Pandey M. K. Wang H. Khera P. Vishwakarma M. K. Kale S. M. Culbreath A. K. (2017). Genetic Dissection of Novel QTLs for Resistance to Leaf Spots and Tomato Spotted Wilt Virus in Peanut (*Arachis hypogaea* L.). Front. Plant Sci. 8, 1. 10.3389/fpls.2017.00025 28197153 PMC5281592

[B45] Paux E. Sourdille P. Mackay I. Feuillet C. (2012). Sequence-based Marker Development in Wheat: Advances and Applications to Breeding. Biotechnol. Adv. 30, 1071–1088. 10.1016/j.biotechadv.2011.09.015 21989506

[B46] Pearce S. Kippes N. Chen A. Debernardi J. M. Dubcovsky J. (2016). RNA-seq Studies Using Wheat PHYTOCHROME B and PHYTOCHROME C Mutants Reveal Shared and Specific Functions in the Regulation of Flowering and Shade-Avoidance Pathways. BMC Plant Biol. 16, 1–19. 10.1186/s12870-016-0831-3 27329140 PMC4915087

[B47] Pootakham W. Shearman J. R. Ruang-Areerate P. Sonthirod C. Sangsrakru D. Jomchai N. (2014). Large-scale SNP Discovery through RNA Sequencing and SNP Genotyping by Targeted Enrichment Sequencing in Cassava (Manihot Esculenta Crantz). PLoS One 9, e116028–19. 10.1371/journal.pone.0116028 25551642 PMC4281258

[B48] Raemaekers R. H. (1988). “Helminthosporum Sativum: Disease Complex on Wheat and Sources of Resistance in Zambia,”. Editor Klatt A. R.. Tech. Ed. 10.3/JQUERY-UI.JS. Wheat Prod. constraints Trop. Environ. a Proc. Int. Conf. January 19-23, 1987. Chiang Mai, Thail.

[B49] Ramirez‐Gonzalez R. H. Segovia V. Bird N. Fenwick P. Holdgate S. Berry S. (2015). RNA ‐ S Eq Bulked Segregant Analysis Enables the Identification of High‐resolution Genetic Markers for Breeding in Hexaploid Wheat. Plant Biotechnol. J. 13, 613–624. 10.1111/PBI.12281 25382230

[B50] Ruiz-Sanz J. I. Aurrekoetxea I. del Agua A. R. Ruiz-Larrea M. B. (2007). Detection of Catechol-O-Methyltransferase Val158Met Polymorphism by a Simple One-step Tetra-Primer Amplification Refractory Mutation System-PCR. Mol. Cell Probes 21, 202–207. 10.1016/j.mcp.2006.12.001 17337160

[B51] Saari E. E. Prescott J. M. (1975). A Scale for Appraising the Foliar Intensity of Wheat Diseases. Plant Dis. Rep. 59, 377–380. Available at: https://eurekamag.com/research/000/008/000008657.php (Accessed January 18, 2022).

[B52] Saghai-Maroof M. A. Soliman K. M. Jorgensen R. A. Allard R. W. (1984). Ribosomal DNA Spacer-Length Polymorphisms in Barley: Mendelian Inheritance, Chromosomal Location, and Population Dynamics. Proc. Natl. Acad. Sci. 81, 8014–8018. 10.1073/PNAS.81.24.8014 6096873 PMC392284

[B53] Saxesena R. R. Mishra V. K. Chand R. Chowdhury A. K. Bhattacharya P. M. Joshi A. K. (2017). Pooling Together Spot Blotch Resistance, High Yield with Earliness in Wheat for Eastern Gangetic Plains of South Asia. Field Crops Res. 214, 291–300. 10.1016/j.fcr.2017.08.027

[B54] Schneeberger K. Weigel D. (2011). Fast-forward Genetics Enabled by New Sequencing Technologies. Trends Plant Sci. 16, 282–288. 10.1016/j.tplants.2011.02.006 21439889

[B55] Shaner G. Finney R. E. (1977). The Effect of Nitrogen Fertilization on the Expression of Slow-Mildewing Resistance in Knox Wheat. Phytopathology 77, 1051. 10.1094/phyto-67-1051 40934422

[B56] Sidhu G. Mohan A. Zheng P. Dhaliwal A. K. Main D. Gill K. S. (2015). Sequencing-based High Throughput Mutation Detection in Bread Wheat. BMC Genomics 16, 962. 10.1186/S12864-015-2112-1/TABLES/1 26578187 PMC4650848

[B57] Singh B. D. Singh A. K. (2015). Marker-Assisted Plant Breeding: Principles and Practices. 10.1007/978-81-322-2316-0

[B58] Singh P. He X. Sansaloni C. Juliana P. Dreisigacker S. Duveiller E. (2018). Resistance to Spot Blotch in Two Mapping Populations of Common Wheat Is Controlled by Multiple QTL of Minor Effects. Ijms 19, 4054. 10.3390/IJMS19124054 PMC6321084 30558200

[B59] Singh V. K. Khan A. W. Saxena R. K. Kumar V. Kale S. M. Sinha P. (2016). Next‐generation Sequencing for Identification of Candidate Genes for Fusarium Wilt and Sterility Mosaic Disease in Pigeonpea ( C Ajanus Cajan ). Plant Biotechnol. J. 14, 1183–1194. 10.1111/PBI.12470 26397045 PMC5054876

[B60] Takagi H. Abe A. Yoshida K. Kosugi S. Natsume S. Mitsuoka C. (2013). QTL-seq: Rapid Mapping of Quantitative Trait Loci in rice by Whole Genome Resequencing of DNA from Two Bulked Populations. Plant J. 74, 174–183. 10.1111/TPJ.12105 23289725

[B61] Tomar V. Singh D. Dhillon G. S. Singh R. P. Poland J. Joshi A. K. (2021). New QTLs for Spot Blotch Disease Resistance in Wheat (*Triticum aestivum* L.) Using Genome-wide Association Mapping. Front. Genet. 11, 1. 10.3389/fgene.2020.613217 PMC7841440 33519916

[B62] Trick M. Adamski N. M. Mugford S. G. Jiang C. C. Febrer M. Uauy C. (2012). Combining SNP Discovery from Next-Generation Sequencing Data with Bulked Segregant Analysis (BSA) to fine-map Genes in Polyploid Wheat. BMC Plant Biol. 12, 14–17. 10.1186/1471-2229-12-14/FIGURES/7 22280551 PMC3296661

[B63] Westermann A. J. Gorski S. A. Vogel J. (2012). Dual RNA-Seq of Pathogen and Host. Nat. Rev. Microbiol. 10, 618–630. 10.1038/NRMICRO2852 22890146

[B64] Wicker T. Gundlach H. Spannagl M. Uauy C. Borrill P. Ramírez-González R. H. (2018). Impact of Transposable Elements on Genome Structure and Evolution in Bread Wheat. Genome Biol. 19, 103–118. 10.1186/S13059-018-1479-0/FIGURES/8 30115100 PMC6097303

[B65] Xie J. Guo G. Wang Y. Hu T. Wang L. Li J. (2020). A rare single nucleotide variant in Pm5e confers powdery mildew resistance in common wheat. New Phytol. 228, 1011–1026. 10.1111/NPH.16762 32569398

[B66] Xu X. Bai G. (2015). Whole-genome Resequencing: Changing the Paradigms of SNP Detection, Molecular Mapping and Gene Discovery. Mol. Breed. 35, 1. 10.1007/s11032-015-0240-6

[B67] Ye S. Dhillon S. Ke X. Collins A. R. Day I. N. M. (2001). An Efficient Procedure for Genotyping Single Nucleotide Polymorphisms. Nucleic Acids Res. 29, 88e–88. 10.1093/NAR/29.17.E88 PMC55900 11522844

[B68] You F. M. Huo N. Gu Y. Q. Luo M. C. Ma Y. Hane D. (2008). BatchPrimer3: A High Throughput Web Application for PCR and Sequencing Primer Design. BMC Bioinformatics 9, 253. 10.1186/1471-2105-9-253/TABLES/2 18510760 PMC2438325

[B69] Zhang P. Guo G. Wu Q. Chen Y. Xie J. Lu P. (2020). Identification and fine Mapping of Spot Blotch (Bipolaris Sorokiniana) Resistance Gene Sb4 in Wheat. Theor. Appl. Genet. 133, 2451–2459. 10.1007/S00122-020-03610-3/TABLES/3 32451599

[B70] Zhong Z. Norvienyeku J. Yu J. Chen M. Cai R. Hong Y. (2015). Two Different Subcellular-Localized Acetoacetyl-CoA Acetyltransferases Differentiate Diverse Functions in Magnaporthe Oryzae. Fungal Genet. Biol. 83, 58–67. 10.1016/j.fgb.2015.08.008 26318870

[B71] Zou C. Wang P. Xu Y. (2016). Bulked Sample Analysis in Genetics, Genomics and Crop Improvement. Plant Biotechnol. J. 14, 1941–1955. 10.1111/pbi.12559 26990124 PMC5043468

