# Supplementary material for: SNP Discovery Using BSR-Seq Approach for Spot Blotch Resistance in Wheat (Triticum aestivum L.), an Essential Crop for Food Security
Source: Front Genet. 2022 Apr 5;13:859676. doi: 10.3389/fgene.2022.859676 (PMC9016274; doi:10.3389/fgene.2022.859676)
Supplement: Supplementary file 2 [file Table1.DOCX]

**Table S1**List of transcripts carrying polymorphic SNPs with their chromosomal locations.

| **#Chr.** | **Source** | **Feature** | **Start** | **End** | **Strand** |
| --- | --- | --- | --- | --- | --- |
| 1A | Blast | gnl\|UG\|Ta#S17889526\|550 | 151887 | 151887 | - |
| 1A | Blast | gnl\|UG\|Ta#S17889526\|628 | 151809 | 151809 | - |
| 1A | Blast | gnl\|UG\|Ta#S22381345\|162 | 230365379 | 230365379 | + |
| 1A | Blast | gnl\|UG\|Ta#S22381345\|199 | 230365416 | 230365416 | + |
| 1A | Blast | gnl\|UG\|Ta#S22381345\|234 | 230365451 | 230365451 | + |
| 1A | Blast | gnl\|UG\|Ta#S22381345\|251 | 230365468 | 230365468 | + |
| 1A | Blast | gnl\|UG\|Ta#S22381345\|259 | 230365476 | 230365476 | + |
| 1A | Blast | gnl\|UG\|Ta#S22381345\|444 | 230365661 | 230365661 | + |
| 1A | Blast | gnl\|UG\|Ta#S22381345\|449 | 230365666 | 230365666 | + |
| 1A | Blast | gnl\|UG\|Ta#S22381345\|465 | 230365682 | 230365682 | + |
| 1A | Blast | gnl\|UG\|Ta#S22381345\|619 | 230365836 | 230365836 | + |
| 1A | Blast | gnl\|UG\|Ta#S52541585\|853 | 232673295 | 232673295 | - |
| 1A | Blast | gnl\|UG\|Ta#S52541798\|2137 | 62583278 | 62583278 | + |
| 1A | Blast | gnl\|UG\|Ta#S52541825\|2717 | 63868734 | 63868734 | - |
| 1A | Blast | gnl\|UG\|Ta#S52544859\|1597 | 35170720 | 35170720 | - |
| 1A | Blast | gnl\|UG\|Ta#S58853222\|493 | 120384341 | 120384341 | + |
| 1A | Blast | gnl\|UG\|Ta#S58900599\|926 | 24299632 | 24299632 | - |
| 1A | Blast | gnl\|UG\|Ta#S61638042\|2051 | 237804774 | 237804774 | + |
| 1A | Blast | gnl\|UG\|Ta#S61638042\|2113 | 237804836 | 237804836 | + |
| 1A | Blast | gnl\|UG\|Ta#S61638042\|2273 | 237804996 | 237804996 | + |
| 1A | Blast | gnl\|UG\|Ta#S61651001\|1051 | 240383020 | 240383020 | + |
| 1A | Blast | gnl\|UG\|Ta#S61651001\|862 | 240382831 | 240382831 | + |
| 1A | Blast | gnl\|UG\|Ta#S61776090\|286 | 173324203 | 173324203 | - |
| 1A | Blast | gnl\|UG\|Ta#S61807784\|1018 | 171131126 | 171131126 | - |
| 1A | Blast | gnl\|UG\|Ta#S61810309\|356 | 13389677 | 13389677 | + |
| 1A | Blast | gnl\|UG\|Ta#S61810309\|410 | 13389731 | 13389731 | + |
| 1A | Blast | gnl\|UG\|Ta#S61810309\|509 | 13389830 | 13389830 | + |
| 1A | Blast | gnl\|UG\|Ta#S61810931\|1808 | 232684245 | 232684245 | + |
| 1A | Blast | gnl\|UG\|Ta#S61810931\|257 | 232682694 | 232682694 | + |
| 1A | Blast | gnl\|UG\|Ta#S61810931\|377 | 232682814 | 232682814 | + |
| 1B | Blast | gnl\|UG\|Ta#S16202135\|264 | 278385110 | 278385110 | - |
| 1B | Blast | gnl\|UG\|Ta#S16202135\|270 | 278385104 | 278385104 | - |
| 1B | Blast | gnl\|UG\|Ta#S17898910\|233 | 9786432 | 9786432 | - |
| 1B | Blast | gnl\|UG\|Ta#S32500015\|194 | 95633680 | 95633680 | - |
| 1B | Blast | gnl\|UG\|Ta#S32500015\|234 | 95633640 | 95633640 | - |
| 1B | Blast | gnl\|UG\|Ta#S32500015\|283 | 95633591 | 95633591 | - |
| 1B | Blast | gnl\|UG\|Ta#S32500015\|303 | 95633571 | 95633571 | - |
| 1B | Blast | gnl\|UG\|Ta#S32500015\|321 | 95633553 | 95633553 | - |
| 1B | Blast | gnl\|UG\|Ta#S32500015\|331 | 95633543 | 95633543 | - |
| 1B | Blast | gnl\|UG\|Ta#S32500015\|469 | 95633405 | 95633405 | - |
| 1B | Blast | gnl\|UG\|Ta#S32500015\|482 | 95633392 | 95633392 | - |
| 1B | Blast | gnl\|UG\|Ta#S32500015\|552 | 95633322 | 95633322 | - |
| 1B | Blast | gnl\|UG\|Ta#S32574087\|510 | 261723513 | 261723513 | - |
| 1B | Blast | gnl\|UG\|Ta#S52541742\|1341 | 9011652 | 9011652 | - |
| 1B | Blast | gnl\|UG\|Ta#S52546725\|1661 | 266224457 | 266224457 | + |
| 1B | Blast | gnl\|UG\|Ta#S58868475\|276 | 247273282 | 247273282 | + |
| 1B | Blast | gnl\|UG\|Ta#S58906218\|1306 | 145894073 | 145894073 | - |
| 1B | Blast | gnl\|UG\|Ta#S61572680\|346 | 15263646 | 15263646 | - |
| 1B | Blast | gnl\|UG\|Ta#S61610331\|305 | 64264227 | 64264227 | - |
| 1B | Blast | gnl\|UG\|Ta#S61710390\|136 | 186038494 | 186038494 | - |
| 1B | Blast | gnl\|UG\|Ta#S61719428\|98 | 186038532 | 186038532 | - |
| 1B | Blast | gnl\|UG\|Ta#S61809476\|320 | 265853870 | 265853870 | + |
| 1B | Blast | gnl\|UG\|Ta#S61809476\|640 | 265854190 | 265854190 | + |
| 1B | Blast | gnl\|UG\|Ta#S61811127\|1581 | 56389469 | 56389469 | + |
| 1B | Blast | gnl\|UG\|Ta#S61811127\|1593 | 56389481 | 56389481 | + |
| 1B | Blast | gnl\|UG\|Ta#S61811127\|1599 | 56389487 | 56389487 | + |
| 1B | Blast | gnl\|UG\|Ta#S61811127\|1605 | 56389493 | 56389493 | + |
| 1B | Blast | gnl\|UG\|Ta#S61811127\|1611 | 56389499 | 56389499 | + |
| 1B | Blast | gnl\|UG\|Ta#S61811127\|1670 | 56389558 | 56389558 | + |
| 1B | Blast | gnl\|UG\|Ta#S61811127\|1683 | 56389571 | 56389571 | + |
| 1B | Blast | gnl\|UG\|Ta#S61811127\|1718 | 56389606 | 56389606 | + |
| 1B | Blast | gnl\|UG\|Ta#S61811127\|1750 | 56389638 | 56389638 | + |
| 1B | Blast | gnl\|UG\|Ta#S61811333\|2240 | 62932287 | 62932287 | + |
| 1B | Blast | gnl\|UG\|Ta#S61811878\|440 | 230841821 | 230841821 | - |
| 1B | Blast | gnl\|UG\|Ta#S61811878\|463 | 230841798 | 230841798 | - |
| 1B | Blast | gnl\|UG\|Ta#S61830892\|404 | 23557961 | 23557961 | + |
| 1D | Blast | gnl\|UG\|Ta#S17889241\|102 | 33580052 | 33580052 | + |
| 1D | Blast | gnl\|UG\|Ta#S26025306\|125 | 47641133 | 47641133 | + |
| 1D | Blast | gnl\|UG\|Ta#S29895624\|1646 | 91605504 | 91605504 | + |
| 1D | Blast | gnl\|UG\|Ta#S32513210\|276 | 63935785 | 63935785 | - |
| 1D | Blast | gnl\|UG\|Ta#S32513210\|363 | 63935698 | 63935698 | - |
| 1D | Blast | gnl\|UG\|Ta#S32513210\|444 | 63935617 | 63935617 | - |
| 1D | Blast | gnl\|UG\|Ta#S32513210\|447 | 63935614 | 63935614 | - |
| 1D | Blast | gnl\|UG\|Ta#S32513210\|463 | 63935598 | 63935598 | - |
| 1D | Blast | gnl\|UG\|Ta#S32513210\|487 | 63935574 | 63935574 | - |
| 1D | Blast | gnl\|UG\|Ta#S32513210\|508 | 63935553 | 63935553 | - |
| 1D | Blast | gnl\|UG\|Ta#S32513210\|514 | 63935547 | 63935547 | - |
| 1D | Blast | gnl\|UG\|Ta#S32513210\|530 | 63935531 | 63935531 | - |
| 1D | Blast | gnl\|UG\|Ta#S32513210\|538 | 63935523 | 63935523 | - |
| 1D | Blast | gnl\|UG\|Ta#S32513210\|564 | 63935497 | 63935497 | - |
| 1D | Blast | gnl\|UG\|Ta#S32513210\|599 | 63935462 | 63935462 | - |
| 1D | Blast | gnl\|UG\|Ta#S32513210\|608 | 63935453 | 63935453 | - |
| 1D | Blast | gnl\|UG\|Ta#S32513210\|618 | 63935443 | 63935443 | - |
| 1D | Blast | gnl\|UG\|Ta#S32513210\|633 | 63935428 | 63935428 | - |
| 1D | Blast | gnl\|UG\|Ta#S61602633\|570 | 55133124 | 55133124 | - |
| 1D | Blast | gnl\|UG\|Ta#S61777868\|627 | 135480340 | 135480340 | + |
| 1D | Blast | gnl\|UG\|Ta#S61778422\|287 | 118049591 | 118049591 | + |
| 1D | Blast | gnl\|UG\|Ta#S61779159\|1070 | 118050374 | 118050374 | + |
| 1D | Blast | gnl\|UG\|Ta#S61780291\|253 | 126506185 | 126506185 | - |
| 1D | Blast | gnl\|UG\|Ta#S61801915\|460 | 33387264 | 33387264 | - |
| 2A | Blast | gnl\|UG\|Ta#S17889960\|11 | 19556164 | 19556164 | + |
| 2A | Blast | gnl\|UG\|Ta#S17986621\|374 | 249003984 | 249003984 | + |
| 2A | Blast | gnl\|UG\|Ta#S17986621\|586 | 249004196 | 249004196 | + |
| 2A | Blast | gnl\|UG\|Ta#S17986621\|628 | 249004238 | 249004238 | + |
| 2A | Blast | gnl\|UG\|Ta#S17986621\|648 | 249004258 | 249004258 | + |
| 2A | Blast | gnl\|UG\|Ta#S17986621\|672 | 249004282 | 249004282 | + |
| 2A | Blast | gnl\|UG\|Ta#S17986732\|273 | 249003883 | 249003883 | + |
| 2A | Blast | gnl\|UG\|Ta#S17986732\|522 | 249004132 | 249004132 | + |
| 2A | Blast | gnl\|UG\|Ta#S17986732\|533 | 249004143 | 249004143 | + |
| 2A | Blast | gnl\|UG\|Ta#S17987957\|809 | 218817908 | 218817908 | + |
| 2A | Blast | gnl\|UG\|Ta#S18006061\|149 | 250530762 | 250530762 | + |
| 2A | Blast | gnl\|UG\|Ta#S18006061\|213 | 250530826 | 250530826 | + |
| 2A | Blast | gnl\|UG\|Ta#S18006061\|222 | 250530835 | 250530835 | + |
| 2A | Blast | gnl\|UG\|Ta#S18006061\|244 | 250530857 | 250530857 | + |
| 2A | Blast | gnl\|UG\|Ta#S22388357\|156 | 17826314 | 17826314 | + |
| 2A | Blast | gnl\|UG\|Ta#S22388357\|225 | 17826383 | 17826383 | + |
| 2A | Blast | gnl\|UG\|Ta#S22388357\|246 | 17826404 | 17826404 | + |
| 2A | Blast | gnl\|UG\|Ta#S22388357\|534 | 17826692 | 17826692 | + |
| 2A | Blast | gnl\|UG\|Ta#S22388357\|540 | 17826698 | 17826698 | + |
| 2A | Blast | gnl\|UG\|Ta#S22388357\|552 | 17826710 | 17826710 | + |
| 2A | Blast | gnl\|UG\|Ta#S37935300\|97 | 245225845 | 245225845 | + |
| 2A | Blast | gnl\|UG\|Ta#S50380304\|212 | 9466479 | 9466479 | + |
| 2A | Blast | gnl\|UG\|Ta#S52546483\|582 | 252362099 | 252362099 | + |
| 2A | Blast | gnl\|UG\|Ta#S52547097\|1909 | 172637223 | 172637223 | + |
| 2A | Blast | gnl\|UG\|Ta#S61541812\|875 | 39840272 | 39840272 | + |
| 2A | Blast | gnl\|UG\|Ta#S61598311\|1021 | 4903552 | 4903552 | - |
| 2A | Blast | gnl\|UG\|Ta#S61598311\|1053 | 4903520 | 4903520 | - |
| 2A | Blast | gnl\|UG\|Ta#S61598311\|930 | 4903643 | 4903643 | - |
| 2A | Blast | gnl\|UG\|Ta#S61598311\|939 | 4903634 | 4903634 | - |
| 2A | Blast | gnl\|UG\|Ta#S61598311\|961 | 4903612 | 4903612 | - |
| 2A | Blast | gnl\|UG\|Ta#S61598311\|975 | 4903598 | 4903598 | - |
| 2A | Blast | gnl\|UG\|Ta#S61598311\|994 | 4903579 | 4903579 | - |
| 2A | Blast | gnl\|UG\|Ta#S61602483\|1519 | 9750660 | 9750660 | + |
| 2A | Blast | gnl\|UG\|Ta#S61602483\|1627 | 9750768 | 9750768 | + |
| 2A | Blast | gnl\|UG\|Ta#S61605237\|195 | 243916251 | 243916251 | + |
| 2A | Blast | gnl\|UG\|Ta#S61620556\|1690 | 243881898 | 243881898 | + |
| 2A | Blast | gnl\|UG\|Ta#S61620556\|1773 | 243881981 | 243881981 | + |
| 2A | Blast | gnl\|UG\|Ta#S61620556\|1936 | 243882144 | 243882144 | + |
| 2A | Blast | gnl\|UG\|Ta#S61632182\|1314 | 244170300 | 244170300 | - |
| 2A | Blast | gnl\|UG\|Ta#S61633084\|350 | 244171264 | 244171264 | - |
| 2A | Blast | gnl\|UG\|Ta#S61633084\|366 | 244171248 | 244171248 | - |
| 2A | Blast | gnl\|UG\|Ta#S61633084\|370 | 244171244 | 244171244 | - |
| 2A | Blast | gnl\|UG\|Ta#S61647161\|614 | 253977240 | 253977240 | + |
| 2A | Blast | gnl\|UG\|Ta#S61781643\|1334 | 148890474 | 148890474 | + |
| 2A | Blast | gnl\|UG\|Ta#S61789925\|607 | 146827192 | 146827192 | - |
| 2A | Blast | gnl\|UG\|Ta#S61801360\|749 | 184504774 | 184504774 | - |
| 2A | Blast | gnl\|UG\|Ta#S61801760\|1463 | 223531022 | 223531022 | + |
| 2A | Blast | gnl\|UG\|Ta#S61808724\|782 | 236555379 | 236555379 | + |
| 2A | Blast | gnl\|UG\|Ta#S61808724\|841 | 236555438 | 236555438 | + |
| 2A | Blast | gnl\|UG\|Ta#S61808724\|842 | 236555439 | 236555439 | + |
| 2A | Blast | gnl\|UG\|Ta#S61808724\|844 | 236555441 | 236555441 | + |
| 2A | Blast | gnl\|UG\|Ta#S61808724\|865 | 236555462 | 236555462 | + |
| 2A | Blast | gnl\|UG\|Ta#S61811572\|132 | 51855274 | 51855274 | + |
| 2A | Blast | gnl\|UG\|Ta#S61811572\|648 | 51855790 | 51855790 | + |
| 2A | Blast | gnl\|UG\|Ta#S61829681\|1260 | 205555850 | 205555850 | + |
| 2A | Blast | gnl\|UG\|Ta#S61829681\|1854 | 205556444 | 205556444 | + |
| 2A | Blast | gnl\|UG\|Ta#S61830097\|1961 | 4893042 | 4893042 | - |
| 2A | Blast | gnl\|UG\|Ta#S61831522\|1880 | 155008328 | 155008328 | - |
| 2A | Blast | gnl\|UG\|Ta#S61832529\|4565 | 8919432 | 8919432 | - |
| 2A | Blast | gnl\|UG\|Ta#S61842040\|425 | 181155498 | 181155498 | - |
| 2A | Blast | gnl\|UG\|Ta#S65592147\|364 | 246676703 | 246676703 | - |
| 2A | Blast | gnl\|UG\|Ta#S65615441\|900 | 238104899 | 238104899 | + |
| 2A | Blast | gnl\|UG\|Ta#S65678324\|701 | 154339689 | 154339689 | - |
| 2B | Blast | gnl\|UG\|Ta#S16058178\|581 | 153132078 | 153132078 | - |
| 2B | Blast | gnl\|UG\|Ta#S16058178\|596 | 153132063 | 153132063 | - |
| 2B | Blast | gnl\|UG\|Ta#S16058178\|791 | 153131868 | 153131868 | - |
| 2B | Blast | gnl\|UG\|Ta#S16058178\|914 | 153131745 | 153131745 | - |
| 2B | Blast | gnl\|UG\|Ta#S26025146\|449 | 56817270 | 56817270 | + |
| 2B | Blast | gnl\|UG\|Ta#S26028863\|690 | 56817939 | 56817939 | + |
| 2B | Blast | gnl\|UG\|Ta#S26028863\|693 | 56817942 | 56817942 | + |
| 2B | Blast | gnl\|UG\|Ta#S26028863\|719 | 56817968 | 56817968 | + |
| 2B | Blast | gnl\|UG\|Ta#S30065898\|992 | 320846105 | 320846105 | - |
| 2B | Blast | gnl\|UG\|Ta#S52541472\|147 | 265149443 | 265149443 | + |
| 2B | Blast | gnl\|UG\|Ta#S52541472\|2364 | 265151660 | 265151660 | + |
| 2B | Blast | gnl\|UG\|Ta#S52544500\|717 | 250663756 | 250663756 | + |
| 2B | Blast | gnl\|UG\|Ta#S52544713\|1374 | 178028836 | 178028836 | + |
| 2B | Blast | gnl\|UG\|Ta#S58899297\|422 | 297644757 | 297644757 | + |
| 2B | Blast | gnl\|UG\|Ta#S58910853\|549 | 265519570 | 265519570 | - |
| 2B | Blast | gnl\|UG\|Ta#S59657920\|972 | 8285341 | 8285341 | - |
| 2B | Blast | gnl\|UG\|Ta#S61592782\|1026 | 282544356 | 282544356 | + |
| 2B | Blast | gnl\|UG\|Ta#S61592813\|1085 | 307892201 | 307892201 | - |
| 2B | Blast | gnl\|UG\|Ta#S61633804\|534 | 37221987 | 37221987 | + |
| 2B | Blast | gnl\|UG\|Ta#S61633804\|662 | 37222115 | 37222115 | + |
| 2B | Blast | gnl\|UG\|Ta#S61650325\|274 | 330746695 | 330746695 | - |
| 2B | Blast | gnl\|UG\|Ta#S61735905\|366 | 300904565 | 300904565 | - |
| 2B | Blast | gnl\|UG\|Ta#S61735905\|530 | 300904401 | 300904401 | - |
| 2B | Blast | gnl\|UG\|Ta#S61735905\|564 | 300904367 | 300904367 | - |
| 2B | Blast | gnl\|UG\|Ta#S61745691\|106 | 309011999 | 309011999 | - |
| 2B | Blast | gnl\|UG\|Ta#S61745691\|285 | 309011820 | 309011820 | - |
| 2B | Blast | gnl\|UG\|Ta#S61778301\|694 | 157480097 | 157480097 | + |
| 2B | Blast | gnl\|UG\|Ta#S61779984\|90 | 111793406 | 111793406 | + |
| 2B | Blast | gnl\|UG\|Ta#S61781836\|1155 | 306481560 | 306481560 | + |
| 2B | Blast | gnl\|UG\|Ta#S61781836\|524 | 306480929 | 306480929 | + |
| 2B | Blast | gnl\|UG\|Ta#S61797708\|1069 | 344240757 | 344240757 | - |
| 2B | Blast | gnl\|UG\|Ta#S61800993\|952 | 309993902 | 309993902 | + |
| 2B | Blast | gnl\|UG\|Ta#S61801381\|270 | 288019963 | 288019963 | - |
| 2B | Blast | gnl\|UG\|Ta#S61809379\|1032 | 15116597 | 15116597 | + |
| 2B | Blast | gnl\|UG\|Ta#S61810399\|1911 | 341466621 | 341466621 | - |
| 2B | Blast | gnl\|UG\|Ta#S61811560\|1014 | 130321587 | 130321587 | - |
| 2B | Blast | gnl\|UG\|Ta#S61811883\|135 | 303716730 | 303716730 | + |
| 2B | Blast | gnl\|UG\|Ta#S61811883\|1536 | 303718131 | 303718131 | + |
| 2B | Blast | gnl\|UG\|Ta#S61811883\|1851 | 303718446 | 303718446 | + |
| 2B | Blast | gnl\|UG\|Ta#S61811883\|1852 | 303718447 | 303718447 | + |
| 2B | Blast | gnl\|UG\|Ta#S61811883\|1887 | 303718482 | 303718482 | + |
| 2B | Blast | gnl\|UG\|Ta#S61812052\|2743 | 336744384 | 336744384 | + |
| 2B | Blast | gnl\|UG\|Ta#S61812348\|3414 | 55514415 | 55514415 | - |
| 2B | Blast | gnl\|UG\|Ta#S61827988\|728 | 323990 | 323990 | - |
| 2B | Blast | gnl\|UG\|Ta#S61829925\|751 | 342065415 | 342065415 | - |
| 2B | Blast | gnl\|UG\|Ta#S61829925\|813 | 342065353 | 342065353 | - |
| 2B | Blast | gnl\|UG\|Ta#S61830137\|1231 | 276676028 | 276676028 | - |
| 2B | Blast | gnl\|UG\|Ta#S61830137\|1257 | 276676002 | 276676002 | - |
| 2B | Blast | gnl\|UG\|Ta#S61830137\|1466 | 276675793 | 276675793 | - |
| 2B | Blast | gnl\|UG\|Ta#S61830137\|684 | 276676575 | 276676575 | - |
| 2B | Blast | gnl\|UG\|Ta#S61831019\|868 | 265420473 | 265420473 | - |
| 2B | Blast | gnl\|UG\|Ta#S61831689\|199 | 186940791 | 186940791 | - |
| 2B | Blast | gnl\|UG\|Ta#S61832175\|1999 | 157700191 | 157700191 | + |
| 2B | Blast | gnl\|UG\|Ta#S61832266\|2996 | 306623596 | 306623596 | - |
| 2B | Blast | gnl\|UG\|Ta#S61832266\|3291 | 306623301 | 306623301 | - |
| 2B | Blast | gnl\|UG\|Ta#S65606752\|2266 | 296891210 | 296891210 | - |
| 2D | Blast | gnl\|UG\|Ta#S26025501\|638 | 10249503 | 10249503 | - |
| 2D | Blast | gnl\|UG\|Ta#S26028338\|742 | 33901445 | 33901445 | + |
| 2D | Blast | gnl\|UG\|Ta#S37919478\|617 | 146303469 | 146303469 | + |
| 2D | Blast | gnl\|UG\|Ta#S58863224\|121 | 75981498 | 75981498 | + |
| 2D | Blast | gnl\|UG\|Ta#S61573819\|1009 | 139702722 | 139702722 | + |
| 2D | Blast | gnl\|UG\|Ta#S61617850\|794 | 58784439 | 58784439 | + |
| 2D | Blast | gnl\|UG\|Ta#S61617850\|836 | 58784481 | 58784481 | + |
| 2D | Blast | gnl\|UG\|Ta#S61617850\|924 | 58784569 | 58784569 | + |
| 2D | Blast | gnl\|UG\|Ta#S61617850\|936 | 58784581 | 58784581 | + |
| 2D | Blast | gnl\|UG\|Ta#S61780108\|631 | 23313132 | 23313132 | + |
| 2D | Blast | gnl\|UG\|Ta#S61781619\|289 | 149203788 | 149203788 | + |
| 2D | Blast | gnl\|UG\|Ta#S61781619\|853 | 149204352 | 149204352 | + |
| 2D | Blast | gnl\|UG\|Ta#S61781619\|862 | 149204361 | 149204361 | + |
| 2D | Blast | gnl\|UG\|Ta#S61812496\|1208 | 9662957 | 9662957 | + |
| 2D | Blast | gnl\|UG\|Ta#S65627502\|887 | 126760811 | 126760811 | + |
| 2D | Blast | gnl\|UG\|Ta#S65677297\|606 | 139066883 | 139066883 | - |
| 3A | Blast | gnl\|UG\|Ta#S18005899\|242 | 91724300 | 91724300 | - |
| 3A | Blast | gnl\|UG\|Ta#S52541590\|836 | 9483287 | 9483287 | + |
| 3A | Blast | gnl\|UG\|Ta#S52542250\|1131 | 9456608 | 9456608 | + |
| 3A | Blast | gnl\|UG\|Ta#S52542250\|829 | 9456306 | 9456306 | + |
| 3A | Blast | gnl\|UG\|Ta#S52542250\|944 | 9456421 | 9456421 | + |
| 3A | Blast | gnl\|UG\|Ta#S52542370\|821 | 126119826 | 126119826 | - |
| 3A | Blast | gnl\|UG\|Ta#S58863752\|278 | 2716027 | 2716027 | + |
| 3A | Blast | gnl\|UG\|Ta#S58863752\|320 | 2716069 | 2716069 | + |
| 3A | Blast | gnl\|UG\|Ta#S58863752\|338 | 2716087 | 2716087 | + |
| 3A | Blast | gnl\|UG\|Ta#S58864984\|396 | 13634283 | 13634283 | + |
| 3A | Blast | gnl\|UG\|Ta#S58898292\|115 | 13521952 | 13521952 | + |
| 3A | Blast | gnl\|UG\|Ta#S58898292\|122 | 13521959 | 13521959 | + |
| 3A | Blast | gnl\|UG\|Ta#S58898292\|137 | 13521974 | 13521974 | + |
| 3A | Blast | gnl\|UG\|Ta#S58898292\|170 | 13522007 | 13522007 | + |
| 3A | Blast | gnl\|UG\|Ta#S58898292\|200 | 13522037 | 13522037 | + |
| 3A | Blast | gnl\|UG\|Ta#S58898292\|268 | 13522105 | 13522105 | + |
| 3A | Blast | gnl\|UG\|Ta#S58898292\|605 | 13522442 | 13522442 | + |
| 3A | Blast | gnl\|UG\|Ta#S58898292\|722 | 13522559 | 13522559 | + |
| 3A | Blast | gnl\|UG\|Ta#S58898292\|87 | 13521924 | 13521924 | + |
| 3A | Blast | gnl\|UG\|Ta#S58898292\|90 | 13521927 | 13521927 | + |
| 3A | Blast | gnl\|UG\|Ta#S58898292\|98 | 13521935 | 13521935 | + |
| 3A | Blast | gnl\|UG\|Ta#S61781391\|542 | 10290408 | 10290408 | + |
| 3A | Blast | gnl\|UG\|Ta#S61799160\|1077 | 143109788 | 143109788 | - |
| 3A | Blast | gnl\|UG\|Ta#S61799620\|1103 | 2716701 | 2716701 | + |
| 3A | Blast | gnl\|UG\|Ta#S61799620\|113 | 2715711 | 2715711 | + |
| 3A | Blast | gnl\|UG\|Ta#S61799620\|130 | 2715728 | 2715728 | + |
| 3A | Blast | gnl\|UG\|Ta#S61799620\|200 | 2715798 | 2715798 | + |
| 3A | Blast | gnl\|UG\|Ta#S61799620\|275 | 2715873 | 2715873 | + |
| 3A | Blast | gnl\|UG\|Ta#S61799620\|277 | 2715875 | 2715875 | + |
| 3A | Blast | gnl\|UG\|Ta#S61799620\|286 | 2715884 | 2715884 | + |
| 3A | Blast | gnl\|UG\|Ta#S61799620\|292 | 2715890 | 2715890 | + |
| 3A | Blast | gnl\|UG\|Ta#S61799620\|369 | 2715967 | 2715967 | + |
| 3A | Blast | gnl\|UG\|Ta#S61799620\|377 | 2715975 | 2715975 | + |
| 3A | Blast | gnl\|UG\|Ta#S61799620\|387 | 2715985 | 2715985 | + |
| 3A | Blast | gnl\|UG\|Ta#S61799620\|412 | 2716010 | 2716010 | + |
| 3A | Blast | gnl\|UG\|Ta#S61809492\|1578 | 157174016 | 157174016 | - |
| 3A | Blast | gnl\|UG\|Ta#S61809492\|307 | 157175287 | 157175287 | - |
| 3A | Blast | gnl\|UG\|Ta#S61809492\|418 | 157175176 | 157175176 | - |
| 3A | Blast | gnl\|UG\|Ta#S61811347\|1180 | 12948664 | 12948664 | + |
| 3A | Blast | gnl\|UG\|Ta#S61811347\|1353 | 12948837 | 12948837 | + |
| 3A | Blast | gnl\|UG\|Ta#S61811347\|441 | 12947925 | 12947925 | + |
| 3A | Blast | gnl\|UG\|Ta#S61811406\|442 | 130994706 | 130994706 | + |
| 3A | Blast | gnl\|UG\|Ta#S61811780\|1079 | 13613078 | 13613078 | + |
| 3A | Blast | gnl\|UG\|Ta#S61811780\|412 | 13612411 | 13612411 | + |
| 3A | Blast | gnl\|UG\|Ta#S61811780\|985 | 13612984 | 13612984 | + |
| 3A | Blast | gnl\|UG\|Ta#S61829914\|1884 | 180733723 | 180733723 | + |
| 3A | Blast | gnl\|UG\|Ta#S61832001\|1325 | 152294108 | 152294108 | + |
| 3A | Blast | gnl\|UG\|Ta#S61832001\|1432 | 152294215 | 152294215 | + |
| 3A | Blast | gnl\|UG\|Ta#S61832001\|2297 | 152295080 | 152295080 | + |
| 3B | Blast | gnl\|UG\|Ta#S12922941\|1996 | 322170268 | 322170268 | + |
| 3B | Blast | gnl\|UG\|Ta#S16833772\|2379 | 485602620 | 485602620 | + |
| 3B | Blast | gnl\|UG\|Ta#S16833772\|2413 | 485602654 | 485602654 | + |
| 3B | Blast | gnl\|UG\|Ta#S16833772\|2462 | 485602703 | 485602703 | + |
| 3B | Blast | gnl\|UG\|Ta#S16833772\|2495 | 485602736 | 485602736 | + |
| 3B | Blast | gnl\|UG\|Ta#S16833772\|9 | 485600250 | 485600250 | + |
| 3B | Blast | gnl\|UG\|Ta#S17893209\|516 | 44036701 | 44036701 | - |
| 3B | Blast | gnl\|UG\|Ta#S17985017\|806 | 75517623 | 75517623 | + |
| 3B | Blast | gnl\|UG\|Ta#S17989003\|488 | 34315481 | 34315481 | + |
| 3B | Blast | gnl\|UG\|Ta#S18012040\|82 | 42456141 | 42456141 | - |
| 3B | Blast | gnl\|UG\|Ta#S26026942\|231 | 17308908 | 17308908 | + |
| 3B | Blast | gnl\|UG\|Ta#S26026942\|450 | 17309127 | 17309127 | + |
| 3B | Blast | gnl\|UG\|Ta#S26026942\|459 | 17309136 | 17309136 | + |
| 3B | Blast | gnl\|UG\|Ta#S26026942\|468 | 17309145 | 17309145 | + |
| 3B | Blast | gnl\|UG\|Ta#S26026942\|525 | 17309202 | 17309202 | + |
| 3B | Blast | gnl\|UG\|Ta#S26026942\|534 | 17309211 | 17309211 | + |
| 3B | Blast | gnl\|UG\|Ta#S52129021\|424 | 457960535 | 457960535 | - |
| 3B | Blast | gnl\|UG\|Ta#S52129021\|741 | 457960218 | 457960218 | - |
| 3B | Blast | gnl\|UG\|Ta#S52543249\|1091 | 26163053 | 26163053 | + |
| 3B | Blast | gnl\|UG\|Ta#S52543283\|585 | 2098322 | 2098322 | + |
| 3B | Blast | gnl\|UG\|Ta#S52543283\|735 | 2098472 | 2098472 | + |
| 3B | Blast | gnl\|UG\|Ta#S52544467\|247 | 89272839 | 89272839 | + |
| 3B | Blast | gnl\|UG\|Ta#S52544534\|1473 | 152084532 | 152084532 | - |
| 3B | Blast | gnl\|UG\|Ta#S52545055\|143 | 89304314 | 89304314 | - |
| 3B | Blast | gnl\|UG\|Ta#S52545062\|1353 | 614925872 | 614925872 | + |
| 3B | Blast | gnl\|UG\|Ta#S52545802\|200 | 3988690 | 3988690 | - |
| 3B | Blast | gnl\|UG\|Ta#S52546851\|554 | 751048926 | 751048926 | + |
| 3B | Blast | gnl\|UG\|Ta#S52546851\|703 | 751049075 | 751049075 | + |
| 3B | Blast | gnl\|UG\|Ta#S58842168\|320 | 436502866 | 436502866 | + |
| 3B | Blast | gnl\|UG\|Ta#S58864433\|315 | 475061916 | 475061916 | - |
| 3B | Blast | gnl\|UG\|Ta#S58898840\|726 | 459818988 | 459818988 | + |
| 3B | Blast | gnl\|UG\|Ta#S58906144\|2365 | 103892970 | 103892970 | + |
| 3B | Blast | gnl\|UG\|Ta#S58910692\|1894 | 194755556 | 194755556 | - |
| 3B | Blast | gnl\|UG\|Ta#S58910692\|1909 | 194755541 | 194755541 | - |
| 3B | Blast | gnl\|UG\|Ta#S61516359\|1148 | 447124490 | 447124490 | - |
| 3B | Blast | gnl\|UG\|Ta#S61530725\|421 | 447561778 | 447561778 | + |
| 3B | Blast | gnl\|UG\|Ta#S61557799\|132 | 712765748 | 712765748 | - |
| 3B | Blast | gnl\|UG\|Ta#S61560716\|641 | 478796711 | 478796711 | + |
| 3B | Blast | gnl\|UG\|Ta#S61573095\|169 | 11960628 | 11960628 | - |
| 3B | Blast | gnl\|UG\|Ta#S61573095\|281 | 11960516 | 11960516 | - |
| 3B | Blast | gnl\|UG\|Ta#S61573095\|325 | 11960472 | 11960472 | - |
| 3B | Blast | gnl\|UG\|Ta#S61573095\|391 | 11960406 | 11960406 | - |
| 3B | Blast | gnl\|UG\|Ta#S61573095\|468 | 11960329 | 11960329 | - |
| 3B | Blast | gnl\|UG\|Ta#S61573095\|539 | 11960258 | 11960258 | - |
| 3B | Blast | gnl\|UG\|Ta#S61573095\|596 | 11960201 | 11960201 | - |
| 3B | Blast | gnl\|UG\|Ta#S61573095\|609 | 11960188 | 11960188 | - |
| 3B | Blast | gnl\|UG\|Ta#S61573095\|632 | 11960165 | 11960165 | - |
| 3B | Blast | gnl\|UG\|Ta#S61573095\|744 | 11960053 | 11960053 | - |
| 3B | Blast | gnl\|UG\|Ta#S61573095\|789 | 11960008 | 11960008 | - |
| 3B | Blast | gnl\|UG\|Ta#S61576881\|750 | 183735976 | 183735976 | - |
| 3B | Blast | gnl\|UG\|Ta#S61579888\|928 | 761689465 | 761689465 | + |
| 3B | Blast | gnl\|UG\|Ta#S61583672\|856 | 561154154 | 561154154 | - |
| 3B | Blast | gnl\|UG\|Ta#S61590322\|498 | 453129744 | 453129744 | + |
| 3B | Blast | gnl\|UG\|Ta#S61595895\|62 | 746240275 | 746240275 | - |
| 3B | Blast | gnl\|UG\|Ta#S61599331\|1632 | 761749055 | 761749055 | - |
| 3B | Blast | gnl\|UG\|Ta#S61599331\|2478 | 761748209 | 761748209 | - |
| 3B | Blast | gnl\|UG\|Ta#S61599331\|2983 | 761747704 | 761747704 | - |
| 3B | Blast | gnl\|UG\|Ta#S61614799\|1627 | 538184025 | 538184025 | - |
| 3B | Blast | gnl\|UG\|Ta#S61614799\|1793 | 538183859 | 538183859 | - |
| 3B | Blast | gnl\|UG\|Ta#S61614799\|2419 | 538183233 | 538183233 | - |
| 3B | Blast | gnl\|UG\|Ta#S61614799\|724 | 538184928 | 538184928 | - |
| 3B | Blast | gnl\|UG\|Ta#S61614799\|747 | 538184905 | 538184905 | - |
| 3B | Blast | gnl\|UG\|Ta#S61614799\|751 | 538184901 | 538184901 | - |
| 3B | Blast | gnl\|UG\|Ta#S61614799\|753 | 538184899 | 538184899 | - |
| 3B | Blast | gnl\|UG\|Ta#S61621725\|194 | 140590899 | 140590899 | - |
| 3B | Blast | gnl\|UG\|Ta#S61626159\|98 | 489312657 | 489312657 | + |
| 3B | Blast | gnl\|UG\|Ta#S61640080\|4891 | 62807490 | 62807490 | - |
| 3B | Blast | gnl\|UG\|Ta#S61643777\|236 | 459807590 | 459807590 | + |
| 3B | Blast | gnl\|UG\|Ta#S61643777\|512 | 459807866 | 459807866 | + |
| 3B | Blast | gnl\|UG\|Ta#S61643777\|761 | 459808115 | 459808115 | + |
| 3B | Blast | gnl\|UG\|Ta#S61656847\|104 | 696733749 | 696733749 | - |
| 3B | Blast | gnl\|UG\|Ta#S61656847\|175 | 696733678 | 696733678 | - |
| 3B | Blast | gnl\|UG\|Ta#S61656847\|195 | 696733658 | 696733658 | - |
| 3B | Blast | gnl\|UG\|Ta#S61656847\|198 | 696733655 | 696733655 | - |
| 3B | Blast | gnl\|UG\|Ta#S61656847\|229 | 696733624 | 696733624 | - |
| 3B | Blast | gnl\|UG\|Ta#S61666064\|462 | 13734002 | 13734002 | + |
| 3B | Blast | gnl\|UG\|Ta#S61767892\|1000 | 69306359 | 69306359 | + |
| 3B | Blast | gnl\|UG\|Ta#S61767892\|891 | 69306250 | 69306250 | + |
| 3B | Blast | gnl\|UG\|Ta#S61767892\|944 | 69306303 | 69306303 | + |
| 3B | Blast | gnl\|UG\|Ta#S61771682\|310 | 515359629 | 515359629 | - |
| 3B | Blast | gnl\|UG\|Ta#S61779527\|1400 | 520204029 | 520204029 | + |
| 3B | Blast | gnl\|UG\|Ta#S61779527\|532 | 520203161 | 520203161 | + |
| 3B | Blast | gnl\|UG\|Ta#S61781822\|615 | 496485417 | 496485417 | - |
| 3B | Blast | gnl\|UG\|Ta#S61798512\|397 | 72727242 | 72727242 | - |
| 3B | Blast | gnl\|UG\|Ta#S61799095\|1099 | 1460714 | 1460714 | + |
| 3B | Blast | gnl\|UG\|Ta#S61799095\|1129 | 1460744 | 1460744 | + |
| 3B | Blast | gnl\|UG\|Ta#S61799095\|1150 | 1460765 | 1460765 | + |
| 3B | Blast | gnl\|UG\|Ta#S61799095\|1216 | 1460831 | 1460831 | + |
| 3B | Blast | gnl\|UG\|Ta#S61799095\|1237 | 1460852 | 1460852 | + |
| 3B | Blast | gnl\|UG\|Ta#S61799095\|583 | 1460198 | 1460198 | + |
| 3B | Blast | gnl\|UG\|Ta#S61799095\|958 | 1460573 | 1460573 | + |
| 3B | Blast | gnl\|UG\|Ta#S61799095\|972 | 1460587 | 1460587 | + |
| 3B | Blast | gnl\|UG\|Ta#S61799487\|780 | 596535051 | 596535051 | - |
| 3B | Blast | gnl\|UG\|Ta#S61799487\|985 | 596534846 | 596534846 | - |
| 3B | Blast | gnl\|UG\|Ta#S61799798\|476 | 7800631 | 7800631 | + |
| 3B | Blast | gnl\|UG\|Ta#S61800025\|494 | 72730220 | 72730220 | + |
| 3B | Blast | gnl\|UG\|Ta#S61800524\|1208 | 1133877 | 1133877 | + |
| 3B | Blast | gnl\|UG\|Ta#S61800524\|1217 | 1133886 | 1133886 | + |
| 3B | Blast | gnl\|UG\|Ta#S61800524\|1218 | 1133887 | 1133887 | + |
| 3B | Blast | gnl\|UG\|Ta#S61809620\|1444 | 142564551 | 142564551 | + |
| 3B | Blast | gnl\|UG\|Ta#S61810371\|1433 | 615962625 | 615962625 | - |
| 3B | Blast | gnl\|UG\|Ta#S61810922\|706 | 745700546 | 745700546 | - |
| 3B | Blast | gnl\|UG\|Ta#S61811209\|432 | 501601720 | 501601720 | + |
| 3B | Blast | gnl\|UG\|Ta#S61811209\|741 | 501602029 | 501602029 | + |
| 3B | Blast | gnl\|UG\|Ta#S61811649\|1250 | 479611981 | 479611981 | + |
| 3B | Blast | gnl\|UG\|Ta#S61811649\|1491 | 479612222 | 479612222 | + |
| 3B | Blast | gnl\|UG\|Ta#S61811649\|1661 | 479612392 | 479612392 | + |
| 3B | Blast | gnl\|UG\|Ta#S61811649\|2427 | 479613158 | 479613158 | + |
| 3B | Blast | gnl\|UG\|Ta#S61811649\|436 | 479611167 | 479611167 | + |
| 3B | Blast | gnl\|UG\|Ta#S61811649\|491 | 479611222 | 479611222 | + |
| 3B | Blast | gnl\|UG\|Ta#S61811649\|614 | 479611345 | 479611345 | + |
| 3B | Blast | gnl\|UG\|Ta#S61811649\|735 | 479611466 | 479611466 | + |
| 3B | Blast | gnl\|UG\|Ta#S61811711\|350 | 478855901 | 478855901 | + |
| 3B | Blast | gnl\|UG\|Ta#S61812058\|2561 | 526229883 | 526229883 | - |
| 3B | Blast | gnl\|UG\|Ta#S61826769\|305 | 709850868 | 709850868 | - |
| 3B | Blast | gnl\|UG\|Ta#S61827847\|1607 | 748347717 | 748347717 | - |
| 3B | Blast | gnl\|UG\|Ta#S61827847\|315 | 748349009 | 748349009 | - |
| 3B | Blast | gnl\|UG\|Ta#S61827847\|668 | 748348656 | 748348656 | - |
| 3B | Blast | gnl\|UG\|Ta#S61831352\|2061 | 746237868 | 746237868 | - |
| 3B | Blast | gnl\|UG\|Ta#S61831352\|2077 | 746237852 | 746237852 | - |
| 3B | Blast | gnl\|UG\|Ta#S61831352\|2167 | 746237762 | 746237762 | - |
| 3B | Blast | gnl\|UG\|Ta#S61831352\|2264 | 746237665 | 746237665 | - |
| 3B | Blast | gnl\|UG\|Ta#S61831352\|2372 | 746237557 | 746237557 | - |
| 3B | Blast | gnl\|UG\|Ta#S61831352\|2411 | 746237518 | 746237518 | - |
| 3B | Blast | gnl\|UG\|Ta#S61831352\|2420 | 746237509 | 746237509 | - |
| 3B | Blast | gnl\|UG\|Ta#S61831352\|743 | 746239186 | 746239186 | - |
| 3B | Blast | gnl\|UG\|Ta#S65588812\|560 | 749288065 | 749288065 | + |
| 3B | Blast | gnl\|UG\|Ta#S65588812\|569 | 749288074 | 749288074 | + |
| 3B | Blast | gnl\|UG\|Ta#S65594683\|859 | 494145648 | 494145648 | - |
| 3B | Blast | gnl\|UG\|Ta#S65595343\|475 | 556001208 | 556001208 | - |
| 3B | Blast | gnl\|UG\|Ta#S65595343\|494 | 556001189 | 556001189 | - |
| 3B | Blast | gnl\|UG\|Ta#S65599844\|506 | 741118448 | 741118448 | - |
| 3B | Blast | gnl\|UG\|Ta#S65615594\|415 | 536556267 | 536556267 | + |
| 3B | Blast | gnl\|UG\|Ta#S65618960\|1480 | 559984261 | 559984261 | + |
| 3B | Blast | gnl\|UG\|Ta#S65634839\|244 | 517649865 | 517649865 | + |
| 3B | Blast | gnl\|UG\|Ta#S65714182\|341 | 61882867 | 61882867 | - |
| 3D | Blast | gnl\|UG\|Ta#S17892727\|497 | 6376643 | 6376643 | - |
| 3D | Blast | gnl\|UG\|Ta#S17892727\|636 | 6376504 | 6376504 | - |
| 3D | Blast | gnl\|UG\|Ta#S17984079\|316 | 869175 | 869175 | - |
| 3D | Blast | gnl\|UG\|Ta#S52543774\|324 | 118844683 | 118844683 | - |
| 3D | Blast | gnl\|UG\|Ta#S52543774\|999 | 118844008 | 118844008 | - |
| 3D | Blast | gnl\|UG\|Ta#S52545248\|931 | 51470934 | 51470934 | - |
| 3D | Blast | gnl\|UG\|Ta#S61733205\|373 | 5720785 | 5720785 | + |
| 3D | Blast | gnl\|UG\|Ta#S61733205\|379 | 5720791 | 5720791 | + |
| 3D | Blast | gnl\|UG\|Ta#S61733205\|433 | 5720845 | 5720845 | + |
| 3D | Blast | gnl\|UG\|Ta#S61799737\|1194 | 118100534 | 118100534 | + |
| 3D | Blast | gnl\|UG\|Ta#S61799737\|1296 | 118100636 | 118100636 | + |
| 3D | Blast | gnl\|UG\|Ta#S61811344\|639 | 5777454 | 5777454 | - |
| 3D | Blast | gnl\|UG\|Ta#S61812448\|1469 | 54813362 | 54813362 | + |
| 3D | Blast | gnl\|UG\|Ta#S61831321\|1477 | 118473290 | 118473290 | - |
| 3D | Blast | gnl\|UG\|Ta#S61832364\|1710 | 6758941 | 6758941 | - |
| 3D | Blast | gnl\|UG\|Ta#S61832364\|1728 | 6758923 | 6758923 | - |
| 3D | Blast | gnl\|UG\|Ta#S61832364\|1749 | 6758902 | 6758902 | - |
| 3D | Blast | gnl\|UG\|Ta#S61832534\|2896 | 118395341 | 118395341 | + |
| 3D | Blast | gnl\|UG\|Ta#S61832534\|2910 | 118395355 | 118395355 | + |
| 3D | Blast | gnl\|UG\|Ta#S65629245\|598 | 6093861 | 6093861 | - |
| 3D | Blast | gnl\|UG\|Ta#S65678442\|583 | 48340944 | 48340944 | + |
| 3D | Blast | gnl\|UG\|Ta#S65678442\|613 | 48340974 | 48340974 | + |
| 4A | Blast | gnl\|UG\|Ta#S18008490\|504 | 82791366 | 82791366 | + |
| 4A | Blast | gnl\|UG\|Ta#S52541863\|688 | 172818238 | 172818238 | - |
| 4A | Blast | gnl\|UG\|Ta#S52546542\|1322 | 172335285 | 172335285 | - |
| 4A | Blast | gnl\|UG\|Ta#S58858339\|735 | 128200871 | 128200871 | - |
| 4A | Blast | gnl\|UG\|Ta#S58898908\|192 | 169467517 | 169467517 | + |
| 4A | Blast | gnl\|UG\|Ta#S58898908\|204 | 169467529 | 169467529 | + |
| 4A | Blast | gnl\|UG\|Ta#S61562714\|256 | 127709003 | 127709003 | + |
| 4A | Blast | gnl\|UG\|Ta#S61562714\|502 | 127709249 | 127709249 | + |
| 4A | Blast | gnl\|UG\|Ta#S61565007\|714 | 127710341 | 127710341 | - |
| 4A | Blast | gnl\|UG\|Ta#S61565007\|865 | 127710190 | 127710190 | - |
| 4A | Blast | gnl\|UG\|Ta#S61605216\|1241 | 213585514 | 213585514 | + |
| 4A | Blast | gnl\|UG\|Ta#S61605216\|1246 | 213585519 | 213585519 | + |
| 4A | Blast | gnl\|UG\|Ta#S61605216\|1254 | 213585527 | 213585527 | + |
| 4A | Blast | gnl\|UG\|Ta#S61605216\|1292 | 213585565 | 213585565 | + |
| 4A | Blast | gnl\|UG\|Ta#S61605216\|942 | 213585215 | 213585215 | + |
| 4A | Blast | gnl\|UG\|Ta#S61639865\|456 | 176758130 | 176758130 | - |
| 4A | Blast | gnl\|UG\|Ta#S61770798\|132 | 172802838 | 172802838 | + |
| 4A | Blast | gnl\|UG\|Ta#S61781492\|1370 | 60061740 | 60061740 | - |
| 4A | Blast | gnl\|UG\|Ta#S61781492\|268 | 60062842 | 60062842 | - |
| 4A | Blast | gnl\|UG\|Ta#S61788065\|396 | 173006100 | 173006100 | - |
| 4A | Blast | gnl\|UG\|Ta#S61801919\|730 | 207936359 | 207936359 | - |
| 4A | Blast | gnl\|UG\|Ta#S61801919\|749 | 207936340 | 207936340 | - |
| 4A | Blast | gnl\|UG\|Ta#S61802087\|1247 | 169769650 | 169769650 | - |
| 4A | Blast | gnl\|UG\|Ta#S61802087\|1368 | 169769529 | 169769529 | - |
| 4A | Blast | gnl\|UG\|Ta#S61802087\|1407 | 169769490 | 169769490 | - |
| 4A | Blast | gnl\|UG\|Ta#S61802087\|1555 | 169769342 | 169769342 | - |
| 4A | Blast | gnl\|UG\|Ta#S61809159\|290 | 212245607 | 212245607 | - |
| 4A | Blast | gnl\|UG\|Ta#S61810883\|1382 | 55088415 | 55088415 | + |
| 4A | Blast | gnl\|UG\|Ta#S61830030\|456 | 137976064 | 137976064 | - |
| 4A | Blast | gnl\|UG\|Ta#S61830683\|706 | 95238581 | 95238581 | + |
| 4A | Blast | gnl\|UG\|Ta#S61832384\|1333 | 27742907 | 27742907 | + |
| 4A | Blast | gnl\|UG\|Ta#S61832497\|932 | 212484353 | 212484353 | + |
| 4A | Blast | gnl\|UG\|Ta#S65592786\|110 | 52723497 | 52723497 | + |
| 4A | Blast | gnl\|UG\|Ta#S65611314\|429 | 77227243 | 77227243 | - |
| 4B | Blast | gnl\|UG\|Ta#S26028447\|397 | 1630297 | 1630297 | - |
| 4B | Blast | gnl\|UG\|Ta#S26028447\|421 | 1630273 | 1630273 | - |
| 4B | Blast | gnl\|UG\|Ta#S50382051\|321 | 2313311 | 2313311 | - |
| 4B | Blast | gnl\|UG\|Ta#S50382051\|330 | 2313302 | 2313302 | - |
| 4B | Blast | gnl\|UG\|Ta#S50382051\|346 | 2313286 | 2313286 | - |
| 4B | Blast | gnl\|UG\|Ta#S52542257\|401 | 53006784 | 53006784 | - |
| 4B | Blast | gnl\|UG\|Ta#S52542619\|1746 | 3921473 | 3921473 | + |
| 4B | Blast | gnl\|UG\|Ta#S52543495\|142 | 312242347 | 312242347 | + |
| 4B | Blast | gnl\|UG\|Ta#S52546221\|508 | 232195613 | 232195613 | - |
| 4B | Blast | gnl\|UG\|Ta#S52546780\|528 | 7079971 | 7079971 | + |
| 4B | Blast | gnl\|UG\|Ta#S55450254\|726 | 46078752 | 46078752 | + |
| 4B | Blast | gnl\|UG\|Ta#S58841747\|587 | 46005852 | 46005852 | + |
| 4B | Blast | gnl\|UG\|Ta#S61606701\|336 | 271794816 | 271794816 | - |
| 4B | Blast | gnl\|UG\|Ta#S61620436\|250 | 315275902 | 315275902 | - |
| 4B | Blast | gnl\|UG\|Ta#S61624452\|424 | 316976476 | 316976476 | + |
| 4B | Blast | gnl\|UG\|Ta#S61641003\|577 | 313829381 | 313829381 | - |
| 4B | Blast | gnl\|UG\|Ta#S61686662\|244 | 97302284 | 97302284 | - |
| 4B | Blast | gnl\|UG\|Ta#S61690917\|616 | 97301912 | 97301912 | - |
| 4B | Blast | gnl\|UG\|Ta#S61708636\|376 | 180386111 | 180386111 | + |
| 4B | Blast | gnl\|UG\|Ta#S61764843\|515 | 48909380 | 48909380 | - |
| 4B | Blast | gnl\|UG\|Ta#S61811007\|2298 | 265386656 | 265386656 | - |
| 4B | Blast | gnl\|UG\|Ta#S61832536\|475 | 116889257 | 116889257 | - |
| 4B | Blast | gnl\|UG\|Ta#S61832536\|65 | 116889667 | 116889667 | - |
| 4B | Blast | gnl\|UG\|Ta#S65616217\|324 | 104093729 | 104093729 | + |
| 4B | Blast | gnl\|UG\|Ta#S65616217\|336 | 104093741 | 104093741 | + |
| 4B | Blast | gnl\|UG\|Ta#S65622746\|515 | 210737412 | 210737412 | - |
| 4B | Blast | gnl\|UG\|Ta#S65667218\|1983 | 44301583 | 44301583 | - |
| 4D | Blast | gnl\|UG\|Ta#S17988625\|500 | 118206719 | 118206719 | + |
| 4D | Blast | gnl\|UG\|Ta#S17988625\|503 | 118206722 | 118206722 | + |
| 4D | Blast | gnl\|UG\|Ta#S22369837\|449 | 11678946 | 11678946 | + |
| 4D | Blast | gnl\|UG\|Ta#S26025157\|144 | 13613288 | 13613288 | + |
| 4D | Blast | gnl\|UG\|Ta#S52543359\|1561 | 116571341 | 116571341 | - |
| 4D | Blast | gnl\|UG\|Ta#S61568261\|387 | 25920790 | 25920790 | - |
| 4D | Blast | gnl\|UG\|Ta#S61648141\|436 | 112309976 | 112309976 | + |
| 4D | Blast | gnl\|UG\|Ta#S61648141\|725 | 112310265 | 112310265 | + |
| 4D | Blast | gnl\|UG\|Ta#S61648141\|786 | 112310326 | 112310326 | + |
| 4D | Blast | gnl\|UG\|Ta#S61648258\|634 | 112310174 | 112310174 | + |
| 4D | Blast | gnl\|UG\|Ta#S61648258\|656 | 112310196 | 112310196 | + |
| 4D | Blast | gnl\|UG\|Ta#S61781770\|1528 | 7016408 | 7016408 | - |
| 4D | Blast | gnl\|UG\|Ta#S61808022\|1422 | 20190485 | 20190485 | - |
| 4D | Blast | gnl\|UG\|Ta#S61808927\|1163 | 74233739 | 74233739 | - |
| 4D | Blast | gnl\|UG\|Ta#S61809486\|1058 | 37497307 | 37497307 | - |
| 4D | Blast | gnl\|UG\|Ta#S61809711\|1683 | 50469748 | 50469748 | + |
| 4D | Blast | gnl\|UG\|Ta#S61830826\|411 | 106401312 | 106401312 | - |
| 4D | Blast | gnl\|UG\|Ta#S61831751\|1528 | 5989929 | 5989929 | + |
| 5A | Blast | gnl\|UG\|Ta#S12923506\|972 | 100476254 | 100476254 | + |
| 5A | Blast | gnl\|UG\|Ta#S13209056\|57 | 100475339 | 100475339 | + |
| 5A | Blast | gnl\|UG\|Ta#S22366154\|133 | 87252138 | 87252138 | + |
| 5A | Blast | gnl\|UG\|Ta#S22366154\|154 | 87252159 | 87252159 | + |
| 5A | Blast | gnl\|UG\|Ta#S43837843\|1535 | 112434453 | 112434453 | + |
| 5A | Blast | gnl\|UG\|Ta#S43837843\|1588 | 112434506 | 112434506 | + |
| 5A | Blast | gnl\|UG\|Ta#S43837843\|1811 | 112434729 | 112434729 | + |
| 5A | Blast | gnl\|UG\|Ta#S43837843\|2445 | 112435363 | 112435363 | + |
| 5A | Blast | gnl\|UG\|Ta#S43837843\|983 | 112433901 | 112433901 | + |
| 5A | Blast | gnl\|UG\|Ta#S52543318\|458 | 105106765 | 105106765 | + |
| 5A | Blast | gnl\|UG\|Ta#S52545940\|228 | 93826313 | 93826313 | - |
| 5A | Blast | gnl\|UG\|Ta#S52545940\|2417 | 93824124 | 93824124 | - |
| 5A | Blast | gnl\|UG\|Ta#S52545940\|2419 | 93824122 | 93824122 | - |
| 5A | Blast | gnl\|UG\|Ta#S52546362\|4634 | 78239420 | 78239420 | - |
| 5A | Blast | gnl\|UG\|Ta#S58889253\|429 | 100475885 | 100475885 | + |
| 5A | Blast | gnl\|UG\|Ta#S58889253\|474 | 100475930 | 100475930 | + |
| 5A | Blast | gnl\|UG\|Ta#S58900229\|1617 | 146368308 | 146368308 | - |
| 5A | Blast | gnl\|UG\|Ta#S58900229\|1660 | 146368265 | 146368265 | - |
| 5A | Blast | gnl\|UG\|Ta#S58900229\|1828 | 146368097 | 146368097 | - |
| 5A | Blast | gnl\|UG\|Ta#S61574864\|295 | 121103350 | 121103350 | - |
| 5A | Blast | gnl\|UG\|Ta#S61613163\|735 | 131746170 | 131746170 | - |
| 5A | Blast | gnl\|UG\|Ta#S61640184\|364 | 97083259 | 97083259 | + |
| 5A | Blast | gnl\|UG\|Ta#S61640184\|372 | 97083267 | 97083267 | + |
| 5A | Blast | gnl\|UG\|Ta#S61640184\|499 | 97083394 | 97083394 | + |
| 5A | Blast | gnl\|UG\|Ta#S61640184\|666 | 97083561 | 97083561 | + |
| 5A | Blast | gnl\|UG\|Ta#S61640184\|739 | 97083634 | 97083634 | + |
| 5A | Blast | gnl\|UG\|Ta#S61668254\|512 | 77641542 | 77641542 | - |
| 5A | Blast | gnl\|UG\|Ta#S61668254\|514 | 77641540 | 77641540 | - |
| 5A | Blast | gnl\|UG\|Ta#S61668254\|550 | 77641504 | 77641504 | - |
| 5A | Blast | gnl\|UG\|Ta#S61678564\|154 | 77641900 | 77641900 | - |
| 5A | Blast | gnl\|UG\|Ta#S61777625\|718 | 98740995 | 98740995 | - |
| 5A | Blast | gnl\|UG\|Ta#S61780199\|1150 | 77243603 | 77243603 | - |
| 5A | Blast | gnl\|UG\|Ta#S61780199\|754 | 77243999 | 77243999 | - |
| 5A | Blast | gnl\|UG\|Ta#S61791288\|1010 | 131595523 | 131595523 | + |
| 5A | Blast | gnl\|UG\|Ta#S61802475\|1431 | 75698010 | 75698010 | + |
| 5A | Blast | gnl\|UG\|Ta#S61808102\|365 | 98739517 | 98739517 | - |
| 5A | Blast | gnl\|UG\|Ta#S61812310\|548 | 96125942 | 96125942 | + |
| 5A | Blast | gnl\|UG\|Ta#S61830453\|828 | 145266133 | 145266133 | + |
| 5A | Blast | gnl\|UG\|Ta#S61832444\|3085 | 57813864 | 57813864 | + |
| 5A | Blast | gnl\|UG\|Ta#S61832444\|3087 | 57813866 | 57813866 | + |
| 5A | Blast | gnl\|UG\|Ta#S61832444\|3149 | 57813928 | 57813928 | + |
| 5A | Blast | gnl\|UG\|Ta#S61832444\|3152 | 57813931 | 57813931 | + |
| 5A | Blast | gnl\|UG\|Ta#S65593968\|104 | 145537207 | 145537207 | - |
| 5A | Blast | gnl\|UG\|Ta#S65593968\|105 | 145537206 | 145537206 | - |
| 5A | Blast | gnl\|UG\|Ta#S65593968\|110 | 145537201 | 145537201 | - |
| 5A | Blast | gnl\|UG\|Ta#S65602700\|612 | 57414880 | 57414880 | - |
| 5A | Blast | gnl\|UG\|Ta#S65626621\|1132 | 142861930 | 142861930 | + |
| 5A | Blast | gnl\|UG\|Ta#S65626621\|1136 | 142861934 | 142861934 | + |
| 5A | Blast | gnl\|UG\|Ta#S65661905\|139 | 123471411 | 123471411 | + |
| 5A | Blast | gnl\|UG\|Ta#S65661905\|143 | 123471415 | 123471415 | + |
| 5B | Blast | gnl\|UG\|Ta#S16208839\|161 | 194582480 | 194582480 | - |
| 5B | Blast | gnl\|UG\|Ta#S17878740\|165 | 187514166 | 187514166 | + |
| 5B | Blast | gnl\|UG\|Ta#S17985740\|220 | 203239712 | 203239712 | + |
| 5B | Blast | gnl\|UG\|Ta#S17985740\|224 | 203239716 | 203239716 | + |
| 5B | Blast | gnl\|UG\|Ta#S17985740\|257 | 203239749 | 203239749 | + |
| 5B | Blast | gnl\|UG\|Ta#S17985740\|263 | 203239755 | 203239755 | + |
| 5B | Blast | gnl\|UG\|Ta#S17985740\|272 | 203239764 | 203239764 | + |
| 5B | Blast | gnl\|UG\|Ta#S17985740\|281 | 203239773 | 203239773 | + |
| 5B | Blast | gnl\|UG\|Ta#S17985740\|315 | 203239807 | 203239807 | + |
| 5B | Blast | gnl\|UG\|Ta#S17985740\|320 | 203239812 | 203239812 | + |
| 5B | Blast | gnl\|UG\|Ta#S17985740\|392 | 203239884 | 203239884 | + |
| 5B | Blast | gnl\|UG\|Ta#S17985740\|464 | 203239956 | 203239956 | + |
| 5B | Blast | gnl\|UG\|Ta#S17985740\|470 | 203239962 | 203239962 | + |
| 5B | Blast | gnl\|UG\|Ta#S17985740\|488 | 203239980 | 203239980 | + |
| 5B | Blast | gnl\|UG\|Ta#S17985740\|494 | 203239986 | 203239986 | + |
| 5B | Blast | gnl\|UG\|Ta#S17985740\|500 | 203239992 | 203239992 | + |
| 5B | Blast | gnl\|UG\|Ta#S17985740\|536 | 203240028 | 203240028 | + |
| 5B | Blast | gnl\|UG\|Ta#S17985740\|542 | 203240034 | 203240034 | + |
| 5B | Blast | gnl\|UG\|Ta#S17985740\|548 | 203240040 | 203240040 | + |
| 5B | Blast | gnl\|UG\|Ta#S17985740\|554 | 203240046 | 203240046 | + |
| 5B | Blast | gnl\|UG\|Ta#S17985740\|787 | 203240279 | 203240279 | + |
| 5B | Blast | gnl\|UG\|Ta#S18011458\|375 | 231601143 | 231601143 | + |
| 5B | Blast | gnl\|UG\|Ta#S22383722\|609 | 63087309 | 63087309 | + |
| 5B | Blast | gnl\|UG\|Ta#S22385781\|349 | 158028527 | 158028527 | + |
| 5B | Blast | gnl\|UG\|Ta#S22612277\|1200 | 203239239 | 203239239 | - |
| 5B | Blast | gnl\|UG\|Ta#S22612277\|1241 | 203239198 | 203239198 | - |
| 5B | Blast | gnl\|UG\|Ta#S22612277\|1263 | 203239176 | 203239176 | - |
| 5B | Blast | gnl\|UG\|Ta#S22612277\|1296 | 203239143 | 203239143 | - |
| 5B | Blast | gnl\|UG\|Ta#S26028272\|70 | 272171726 | 272171726 | - |
| 5B | Blast | gnl\|UG\|Ta#S32652379\|146 | 7457255 | 7457255 | - |
| 5B | Blast | gnl\|UG\|Ta#S32652920\|113 | 204009899 | 204009899 | + |
| 5B | Blast | gnl\|UG\|Ta#S32652920\|72 | 204009858 | 204009858 | + |
| 5B | Blast | gnl\|UG\|Ta#S37763503\|10 | 221656483 | 221656483 | + |
| 5B | Blast | gnl\|UG\|Ta#S52541131\|1186 | 123294297 | 123294297 | + |
| 5B | Blast | gnl\|UG\|Ta#S52541488\|1181 | 260481460 | 260481460 | - |
| 5B | Blast | gnl\|UG\|Ta#S52542520\|1938 | 120731509 | 120731509 | - |
| 5B | Blast | gnl\|UG\|Ta#S52543321\|1198 | 55140643 | 55140643 | - |
| 5B | Blast | gnl\|UG\|Ta#S52543321\|1221 | 55140620 | 55140620 | - |
| 5B | Blast | gnl\|UG\|Ta#S52543321\|1296 | 55140545 | 55140545 | - |
| 5B | Blast | gnl\|UG\|Ta#S52543321\|1899 | 55139942 | 55139942 | - |
| 5B | Blast | gnl\|UG\|Ta#S52543321\|2284 | 55139557 | 55139557 | - |
| 5B | Blast | gnl\|UG\|Ta#S52543321\|2303 | 55139538 | 55139538 | - |
| 5B | Blast | gnl\|UG\|Ta#S52543321\|2966 | 55138875 | 55138875 | - |
| 5B | Blast | gnl\|UG\|Ta#S52543321\|3091 | 55138750 | 55138750 | - |
| 5B | Blast | gnl\|UG\|Ta#S52543321\|3155 | 55138686 | 55138686 | - |
| 5B | Blast | gnl\|UG\|Ta#S52543321\|3276 | 55138565 | 55138565 | - |
| 5B | Blast | gnl\|UG\|Ta#S52543321\|3367 | 55138474 | 55138474 | - |
| 5B | Blast | gnl\|UG\|Ta#S52543321\|349 | 55141492 | 55141492 | - |
| 5B | Blast | gnl\|UG\|Ta#S52543321\|3661 | 55138180 | 55138180 | - |
| 5B | Blast | gnl\|UG\|Ta#S52543321\|3731 | 55138110 | 55138110 | - |
| 5B | Blast | gnl\|UG\|Ta#S52543321\|3781 | 55138060 | 55138060 | - |
| 5B | Blast | gnl\|UG\|Ta#S52543321\|3806 | 55138035 | 55138035 | - |
| 5B | Blast | gnl\|UG\|Ta#S52543321\|3965 | 55137876 | 55137876 | - |
| 5B | Blast | gnl\|UG\|Ta#S52543321\|4263 | 55137578 | 55137578 | - |
| 5B | Blast | gnl\|UG\|Ta#S52543321\|4360 | 55137481 | 55137481 | - |
| 5B | Blast | gnl\|UG\|Ta#S52543321\|4691 | 55137150 | 55137150 | - |
| 5B | Blast | gnl\|UG\|Ta#S52543321\|4739 | 55137102 | 55137102 | - |
| 5B | Blast | gnl\|UG\|Ta#S52544177\|2966 | 206669231 | 206669231 | - |
| 5B | Blast | gnl\|UG\|Ta#S52544521\|1396 | 203553013 | 203553013 | - |
| 5B | Blast | gnl\|UG\|Ta#S52544521\|1801 | 203552608 | 203552608 | - |
| 5B | Blast | gnl\|UG\|Ta#S52544521\|475 | 203553934 | 203553934 | - |
| 5B | Blast | gnl\|UG\|Ta#S52544521\|637 | 203553772 | 203553772 | - |
| 5B | Blast | gnl\|UG\|Ta#S52544911\|2702 | 158309328 | 158309328 | - |
| 5B | Blast | gnl\|UG\|Ta#S52545958\|180 | 99751251 | 99751251 | - |
| 5B | Blast | gnl\|UG\|Ta#S52545958\|311 | 99751120 | 99751120 | - |
| 5B | Blast | gnl\|UG\|Ta#S52546262\|23 | 184645457 | 184645457 | + |
| 5B | Blast | gnl\|UG\|Ta#S52546885\|2388 | 208031035 | 208031035 | - |
| 5B | Blast | gnl\|UG\|Ta#S52546885\|2404 | 208031019 | 208031019 | - |
| 5B | Blast | gnl\|UG\|Ta#S58895377\|924 | 2718320 | 2718320 | - |
| 5B | Blast | gnl\|UG\|Ta#S58901456\|153 | 267695899 | 267695899 | - |
| 5B | Blast | gnl\|UG\|Ta#S58901456\|158 | 267695894 | 267695894 | - |
| 5B | Blast | gnl\|UG\|Ta#S58902006\|707 | 173295947 | 173295947 | + |
| 5B | Blast | gnl\|UG\|Ta#S61516468\|1158 | 106621329 | 106621329 | - |
| 5B | Blast | gnl\|UG\|Ta#S61523988\|349 | 91122732 | 91122732 | + |
| 5B | Blast | gnl\|UG\|Ta#S61523988\|352 | 91122735 | 91122735 | + |
| 5B | Blast | gnl\|UG\|Ta#S61523988\|356 | 91122739 | 91122739 | + |
| 5B | Blast | gnl\|UG\|Ta#S61523988\|360 | 91122743 | 91122743 | + |
| 5B | Blast | gnl\|UG\|Ta#S61559658\|1021 | 93593095 | 93593095 | + |
| 5B | Blast | gnl\|UG\|Ta#S61574500\|1088 | 267630054 | 267630054 | + |
| 5B | Blast | gnl\|UG\|Ta#S61606146\|129 | 223821290 | 223821290 | - |
| 5B | Blast | gnl\|UG\|Ta#S61615691\|327 | 111346975 | 111346975 | + |
| 5B | Blast | gnl\|UG\|Ta#S61615691\|338 | 111346986 | 111346986 | + |
| 5B | Blast | gnl\|UG\|Ta#S61619919\|369 | 187480905 | 187480905 | - |
| 5B | Blast | gnl\|UG\|Ta#S61626670\|411 | 258425084 | 258425084 | - |
| 5B | Blast | gnl\|UG\|Ta#S61626670\|491 | 258425004 | 258425004 | - |
| 5B | Blast | gnl\|UG\|Ta#S61626670\|509 | 258424986 | 258424986 | - |
| 5B | Blast | gnl\|UG\|Ta#S61626670\|539 | 258424956 | 258424956 | - |
| 5B | Blast | gnl\|UG\|Ta#S61640166\|1341 | 146820115 | 146820115 | + |
| 5B | Blast | gnl\|UG\|Ta#S61640166\|529 | 146819303 | 146819303 | + |
| 5B | Blast | gnl\|UG\|Ta#S61644963\|349 | 218715229 | 218715229 | + |
| 5B | Blast | gnl\|UG\|Ta#S61686233\|130 | 200722490 | 200722490 | - |
| 5B | Blast | gnl\|UG\|Ta#S61686233\|142 | 200722478 | 200722478 | - |
| 5B | Blast | gnl\|UG\|Ta#S61686233\|151 | 200722469 | 200722469 | - |
| 5B | Blast | gnl\|UG\|Ta#S61686233\|382 | 200722238 | 200722238 | - |
| 5B | Blast | gnl\|UG\|Ta#S61708094\|225 | 209884729 | 209884729 | - |
| 5B | Blast | gnl\|UG\|Ta#S61736392\|366 | 257678496 | 257678496 | + |
| 5B | Blast | gnl\|UG\|Ta#S61736392\|378 | 257678508 | 257678508 | + |
| 5B | Blast | gnl\|UG\|Ta#S61736392\|426 | 257678556 | 257678556 | + |
| 5B | Blast | gnl\|UG\|Ta#S61736392\|434 | 257678564 | 257678564 | + |
| 5B | Blast | gnl\|UG\|Ta#S61737110\|158 | 258863521 | 258863521 | - |
| 5B | Blast | gnl\|UG\|Ta#S61780368\|1112 | 214889778 | 214889778 | + |
| 5B | Blast | gnl\|UG\|Ta#S61781056\|1336 | 200832836 | 200832836 | - |
| 5B | Blast | gnl\|UG\|Ta#S61781056\|1388 | 200832784 | 200832784 | - |
| 5B | Blast | gnl\|UG\|Ta#S61781056\|1420 | 200832752 | 200832752 | - |
| 5B | Blast | gnl\|UG\|Ta#S61781056\|1429 | 200832743 | 200832743 | - |
| 5B | Blast | gnl\|UG\|Ta#S61781056\|1449 | 200832723 | 200832723 | - |
| 5B | Blast | gnl\|UG\|Ta#S61781056\|1452 | 200832720 | 200832720 | - |
| 5B | Blast | gnl\|UG\|Ta#S61781056\|1467 | 200832705 | 200832705 | - |
| 5B | Blast | gnl\|UG\|Ta#S61781056\|1483 | 200832689 | 200832689 | - |
| 5B | Blast | gnl\|UG\|Ta#S61781056\|1493 | 200832679 | 200832679 | - |
| 5B | Blast | gnl\|UG\|Ta#S61781056\|1503 | 200832669 | 200832669 | - |
| 5B | Blast | gnl\|UG\|Ta#S61781056\|1525 | 200832647 | 200832647 | - |
| 5B | Blast | gnl\|UG\|Ta#S61781638\|1121 | 198995047 | 198995047 | - |
| 5B | Blast | gnl\|UG\|Ta#S61781638\|829 | 198995339 | 198995339 | - |
| 5B | Blast | gnl\|UG\|Ta#S61782457\|364 | 121971974 | 121971974 | + |
| 5B | Blast | gnl\|UG\|Ta#S61782457\|486 | 121972096 | 121972096 | + |
| 5B | Blast | gnl\|UG\|Ta#S61782457\|562 | 121972172 | 121972172 | + |
| 5B | Blast | gnl\|UG\|Ta#S61788465\|284 | 197469892 | 197469892 | + |
| 5B | Blast | gnl\|UG\|Ta#S61799906\|242 | 254045791 | 254045791 | - |
| 5B | Blast | gnl\|UG\|Ta#S61799906\|251 | 254045782 | 254045782 | - |
| 5B | Blast | gnl\|UG\|Ta#S61799906\|482 | 254045551 | 254045551 | - |
| 5B | Blast | gnl\|UG\|Ta#S61800926\|1265 | 192690629 | 192690629 | - |
| 5B | Blast | gnl\|UG\|Ta#S61800926\|614 | 192691280 | 192691280 | - |
| 5B | Blast | gnl\|UG\|Ta#S61800926\|623 | 192691271 | 192691271 | - |
| 5B | Blast | gnl\|UG\|Ta#S61800926\|752 | 192691142 | 192691142 | - |
| 5B | Blast | gnl\|UG\|Ta#S61800926\|821 | 192691073 | 192691073 | - |
| 5B | Blast | gnl\|UG\|Ta#S61801792\|1492 | 217642972 | 217642972 | - |
| 5B | Blast | gnl\|UG\|Ta#S61803349\|308 | 209881612 | 209881612 | - |
| 5B | Blast | gnl\|UG\|Ta#S61803349\|364 | 209881556 | 209881556 | - |
| 5B | Blast | gnl\|UG\|Ta#S61808053\|951 | 205571154 | 205571154 | + |
| 5B | Blast | gnl\|UG\|Ta#S61808571\|1324 | 257681931 | 257681931 | + |
| 5B | Blast | gnl\|UG\|Ta#S61808827\|1331 | 95252160 | 95252160 | + |
| 5B | Blast | gnl\|UG\|Ta#S61810015\|193 | 178675800 | 178675800 | - |
| 5B | Blast | gnl\|UG\|Ta#S61811466\|1038 | 204558635 | 204558635 | - |
| 5B | Blast | gnl\|UG\|Ta#S61811466\|1057 | 204558616 | 204558616 | - |
| 5B | Blast | gnl\|UG\|Ta#S61811805\|1899 | 87758271 | 87758271 | - |
| 5B | Blast | gnl\|UG\|Ta#S61811805\|2328 | 87757842 | 87757842 | - |
| 5B | Blast | gnl\|UG\|Ta#S61811805\|313 | 87759857 | 87759857 | - |
| 5B | Blast | gnl\|UG\|Ta#S61812294\|1817 | 259558216 | 259558216 | + |
| 5B | Blast | gnl\|UG\|Ta#S61812294\|1819 | 259558218 | 259558218 | + |
| 5B | Blast | gnl\|UG\|Ta#S61812294\|1861 | 259558260 | 259558260 | + |
| 5B | Blast | gnl\|UG\|Ta#S61812294\|1898 | 259558297 | 259558297 | + |
| 5B | Blast | gnl\|UG\|Ta#S61812294\|1910 | 259558309 | 259558309 | + |
| 5B | Blast | gnl\|UG\|Ta#S61812294\|1919 | 259558318 | 259558318 | + |
| 5B | Blast | gnl\|UG\|Ta#S61812294\|1942 | 259558341 | 259558341 | + |
| 5B | Blast | gnl\|UG\|Ta#S61812294\|1943 | 259558342 | 259558342 | + |
| 5B | Blast | gnl\|UG\|Ta#S61812294\|1965 | 259558364 | 259558364 | + |
| 5B | Blast | gnl\|UG\|Ta#S61812294\|1985 | 259558384 | 259558384 | + |
| 5B | Blast | gnl\|UG\|Ta#S61812294\|1992 | 259558391 | 259558391 | + |
| 5B | Blast | gnl\|UG\|Ta#S61812294\|2037 | 259558436 | 259558436 | + |
| 5B | Blast | gnl\|UG\|Ta#S61812294\|2076 | 259558475 | 259558475 | + |
| 5B | Blast | gnl\|UG\|Ta#S61812294\|2091 | 259558490 | 259558490 | + |
| 5B | Blast | gnl\|UG\|Ta#S61812294\|2172 | 259558571 | 259558571 | + |
| 5B | Blast | gnl\|UG\|Ta#S61812294\|2205 | 259558604 | 259558604 | + |
| 5B | Blast | gnl\|UG\|Ta#S61812294\|2277 | 259558676 | 259558676 | + |
| 5B | Blast | gnl\|UG\|Ta#S61812294\|2325 | 259558724 | 259558724 | + |
| 5B | Blast | gnl\|UG\|Ta#S61812294\|2383 | 259558782 | 259558782 | + |
| 5B | Blast | gnl\|UG\|Ta#S61812294\|2415 | 259558814 | 259558814 | + |
| 5B | Blast | gnl\|UG\|Ta#S61812294\|2425 | 259558824 | 259558824 | + |
| 5B | Blast | gnl\|UG\|Ta#S61812294\|2466 | 259558865 | 259558865 | + |
| 5B | Blast | gnl\|UG\|Ta#S61812294\|2472 | 259558871 | 259558871 | + |
| 5B | Blast | gnl\|UG\|Ta#S61812294\|2793 | 259559192 | 259559192 | + |
| 5B | Blast | gnl\|UG\|Ta#S61812294\|2810 | 259559209 | 259559209 | + |
| 5B | Blast | gnl\|UG\|Ta#S61812294\|2820 | 259559219 | 259559219 | + |
| 5B | Blast | gnl\|UG\|Ta#S61812294\|2838 | 259559237 | 259559237 | + |
| 5B | Blast | gnl\|UG\|Ta#S61826926\|325 | 177437328 | 177437328 | - |
| 5B | Blast | gnl\|UG\|Ta#S61826926\|331 | 177437322 | 177437322 | - |
| 5B | Blast | gnl\|UG\|Ta#S61828078\|533 | 17172918 | 17172918 | - |
| 5B | Blast | gnl\|UG\|Ta#S61828396\|1065 | 258890685 | 258890685 | - |
| 5B | Blast | gnl\|UG\|Ta#S61828396\|1206 | 258890544 | 258890544 | - |
| 5B | Blast | gnl\|UG\|Ta#S61828801\|702 | 222073464 | 222073464 | - |
| 5B | Blast | gnl\|UG\|Ta#S61829198\|1478 | 213112056 | 213112056 | + |
| 5B | Blast | gnl\|UG\|Ta#S61829605\|1353 | 155104475 | 155104475 | + |
| 5B | Blast | gnl\|UG\|Ta#S61830280\|1478 | 47816829 | 47816829 | - |
| 5B | Blast | gnl\|UG\|Ta#S61830280\|1973 | 47816334 | 47816334 | - |
| 5B | Blast | gnl\|UG\|Ta#S61830716\|1073 | 64038272 | 64038272 | - |
| 5B | Blast | gnl\|UG\|Ta#S61830716\|1250 | 64038095 | 64038095 | - |
| 5B | Blast | gnl\|UG\|Ta#S61830716\|275 | 64039070 | 64039070 | - |
| 5B | Blast | gnl\|UG\|Ta#S61830716\|382 | 64038963 | 64038963 | - |
| 5B | Blast | gnl\|UG\|Ta#S61831515\|2009 | 7204316 | 7204316 | - |
| 5B | Blast | gnl\|UG\|Ta#S61831515\|2019 | 7204306 | 7204306 | - |
| 5B | Blast | gnl\|UG\|Ta#S61831622\|532 | 66880877 | 66880877 | + |
| 5B | Blast | gnl\|UG\|Ta#S61832033\|260 | 80530199 | 80530199 | + |
| 5B | Blast | gnl\|UG\|Ta#S61832033\|2656 | 80532595 | 80532595 | + |
| 5B | Blast | gnl\|UG\|Ta#S65593595\|50 | 183803763 | 183803763 | - |
| 5B | Blast | gnl\|UG\|Ta#S65594113\|1381 | 4723723 | 4723723 | - |
| 5B | Blast | gnl\|UG\|Ta#S65596618\|1124 | 182540454 | 182540454 | + |
| 5B | Blast | gnl\|UG\|Ta#S65606779\|1157 | 217874329 | 217874329 | + |
| 5B | Blast | gnl\|UG\|Ta#S65606779\|304 | 217873476 | 217873476 | + |
| 5B | Blast | gnl\|UG\|Ta#S65688304\|1258 | 201806615 | 201806615 | - |
| 5B | Blast | gnl\|UG\|Ta#S65688304\|1402 | 201806471 | 201806471 | - |
| 5B | Blast | gnl\|UG\|Ta#S65688304\|1522 | 201806351 | 201806351 | - |
| 5B | Blast | gnl\|UG\|Ta#S65688304\|1561 | 201806312 | 201806312 | - |
| 5B | Blast | gnl\|UG\|Ta#S65715070\|585 | 222072769 | 222072769 | - |
| 5B | Blast | gnl\|UG\|Ta#S65715070\|610 | 222072744 | 222072744 | - |
| 5B | Blast | gnl\|UG\|Ta#S65719718\|191 | 246937904 | 246937904 | - |
| 5B | Blast | gnl\|UG\|Ta#S65720200\|623 | 215283275 | 215283275 | - |
| 5B | Blast | gnl\|UG\|Ta#S65721855\|259 | 215283639 | 215283639 | - |
| 5D | Blast | gnl\|UG\|Ta#S12918089\|151 | 105680878 | 105680878 | + |
| 5D | Blast | gnl\|UG\|Ta#S17988074\|695 | 108195240 | 108195240 | - |
| 5D | Blast | gnl\|UG\|Ta#S17988074\|719 | 108195216 | 108195216 | - |
| 5D | Blast | gnl\|UG\|Ta#S52543059\|2889 | 117914514 | 117914514 | - |
| 5D | Blast | gnl\|UG\|Ta#S52544098\|1454 | 151353805 | 151353805 | + |
| 5D | Blast | gnl\|UG\|Ta#S52544098\|719 | 151353070 | 151353070 | + |
| 5D | Blast | gnl\|UG\|Ta#S52544606\|1285 | 106008947 | 106008947 | - |
| 5D | Blast | gnl\|UG\|Ta#S52544642\|1134 | 121426573 | 121426573 | + |
| 5D | Blast | gnl\|UG\|Ta#S52544642\|1239 | 121426678 | 121426678 | + |
| 5D | Blast | gnl\|UG\|Ta#S52544642\|1246 | 121426685 | 121426685 | + |
| 5D | Blast | gnl\|UG\|Ta#S52544642\|1263 | 121426702 | 121426702 | + |
| 5D | Blast | gnl\|UG\|Ta#S52544642\|1287 | 121426726 | 121426726 | + |
| 5D | Blast | gnl\|UG\|Ta#S52544642\|1335 | 121426774 | 121426774 | + |
| 5D | Blast | gnl\|UG\|Ta#S52544642\|1341 | 121426780 | 121426780 | + |
| 5D | Blast | gnl\|UG\|Ta#S52544642\|1372 | 121426811 | 121426811 | + |
| 5D | Blast | gnl\|UG\|Ta#S52544642\|1384 | 121426823 | 121426823 | + |
| 5D | Blast | gnl\|UG\|Ta#S52544642\|1387 | 121426826 | 121426826 | + |
| 5D | Blast | gnl\|UG\|Ta#S52544642\|1430 | 121426869 | 121426869 | + |
| 5D | Blast | gnl\|UG\|Ta#S52544642\|1471 | 121426910 | 121426910 | + |
| 5D | Blast | gnl\|UG\|Ta#S52544642\|1499 | 121426938 | 121426938 | + |
| 5D | Blast | gnl\|UG\|Ta#S52544642\|193 | 121425632 | 121425632 | + |
| 5D | Blast | gnl\|UG\|Ta#S52544642\|229 | 121425668 | 121425668 | + |
| 5D | Blast | gnl\|UG\|Ta#S52544642\|473 | 121425912 | 121425912 | + |
| 5D | Blast | gnl\|UG\|Ta#S52544642\|879 | 121426318 | 121426318 | + |
| 5D | Blast | gnl\|UG\|Ta#S52544642\|999 | 121426438 | 121426438 | + |
| 5D | Blast | gnl\|UG\|Ta#S52545323\|756 | 149347330 | 149347330 | - |
| 5D | Blast | gnl\|UG\|Ta#S52545323\|778 | 149347308 | 149347308 | - |
| 5D | Blast | gnl\|UG\|Ta#S52545323\|845 | 149347241 | 149347241 | - |
| 5D | Blast | gnl\|UG\|Ta#S52545801\|1794 | 135783036 | 135783036 | + |
| 5D | Blast | gnl\|UG\|Ta#S52546340\|1507 | 111575356 | 111575356 | - |
| 5D | Blast | gnl\|UG\|Ta#S52547082\|918 | 136837188 | 136837188 | - |
| 5D | Blast | gnl\|UG\|Ta#S61541173\|552 | 142073658 | 142073658 | - |
| 5D | Blast | gnl\|UG\|Ta#S61558124\|662 | 109191598 | 109191598 | - |
| 5D | Blast | gnl\|UG\|Ta#S61558124\|717 | 109191543 | 109191543 | - |
| 5D | Blast | gnl\|UG\|Ta#S61646833\|362 | 106153707 | 106153707 | - |
| 5D | Blast | gnl\|UG\|Ta#S61646981\|396 | 19291044 | 19291044 | + |
| 5D | Blast | gnl\|UG\|Ta#S61650443\|330 | 137777679 | 137777679 | - |
| 5D | Blast | gnl\|UG\|Ta#S61735589\|374 | 156761116 | 156761116 | + |
| 5D | Blast | gnl\|UG\|Ta#S61735589\|608 | 156761350 | 156761350 | + |
| 5D | Blast | gnl\|UG\|Ta#S61754731\|400 | 154443150 | 154443150 | - |
| 5D | Blast | gnl\|UG\|Ta#S61772146\|669 | 116626894 | 116626894 | - |
| 5D | Blast | gnl\|UG\|Ta#S61791066\|711 | 109564224 | 109564224 | - |
| 5D | Blast | gnl\|UG\|Ta#S61798764\|483 | 138723531 | 138723531 | - |
| 5D | Blast | gnl\|UG\|Ta#S61799676\|798 | 128880533 | 128880533 | + |
| 5D | Blast | gnl\|UG\|Ta#S61799676\|901 | 128880636 | 128880636 | + |
| 5D | Blast | gnl\|UG\|Ta#S61808114\|801 | 130691604 | 130691604 | + |
| 5D | Blast | gnl\|UG\|Ta#S61808114\|805 | 130691608 | 130691608 | + |
| 5D | Blast | gnl\|UG\|Ta#S61808114\|811 | 130691614 | 130691614 | + |
| 5D | Blast | gnl\|UG\|Ta#S61808114\|825 | 130691628 | 130691628 | + |
| 5D | Blast | gnl\|UG\|Ta#S61808114\|848 | 130691651 | 130691651 | + |
| 5D | Blast | gnl\|UG\|Ta#S61808114\|904 | 130691707 | 130691707 | + |
| 5D | Blast | gnl\|UG\|Ta#S61809678\|205 | 130536813 | 130536813 | + |
| 5D | Blast | gnl\|UG\|Ta#S61811916\|2774 | 112797768 | 112797768 | - |
| 5D | Blast | gnl\|UG\|Ta#S61812390\|3513 | 157870107 | 157870107 | + |
| 5D | Blast | gnl\|UG\|Ta#S61829487\|148 | 106996196 | 106996196 | + |
| 5D | Blast | gnl\|UG\|Ta#S61831568\|1477 | 60475184 | 60475184 | - |
| 5D | Blast | gnl\|UG\|Ta#S61831928\|2058 | 159361642 | 159361642 | + |
| 5D | Blast | gnl\|UG\|Ta#S61832070\|728 | 101863577 | 101863577 | + |
| 5D | Blast | gnl\|UG\|Ta#S65594281\|911 | 132161855 | 132161855 | - |
| 5D | Blast | gnl\|UG\|Ta#S65606143\|238 | 111785042 | 111785042 | - |
| 5D | Blast | gnl\|UG\|Ta#S65606143\|251 | 111785029 | 111785029 | - |
| 5D | Blast | gnl\|UG\|Ta#S65606143\|254 | 111785026 | 111785026 | - |
| 5D | Blast | gnl\|UG\|Ta#S65610471\|514 | 156792821 | 156792821 | + |
| 5D | Blast | gnl\|UG\|Ta#S65620334\|1445 | 152511865 | 152511865 | + |
| 5D | Blast | gnl\|UG\|Ta#S65702526\|24 | 120495522 | 120495522 | + |
| 6A | Blast | gnl\|UG\|Ta#S17890903\|426 | 194587448 | 194587448 | - |
| 6A | Blast | gnl\|UG\|Ta#S18007638\|223 | 193781472 | 193781472 | - |
| 6A | Blast | gnl\|UG\|Ta#S18007638\|243 | 193781452 | 193781452 | - |
| 6A | Blast | gnl\|UG\|Ta#S18007638\|262 | 193781433 | 193781433 | - |
| 6A | Blast | gnl\|UG\|Ta#S18007638\|276 | 193781419 | 193781419 | - |
| 6A | Blast | gnl\|UG\|Ta#S18007638\|432 | 193781263 | 193781263 | - |
| 6A | Blast | gnl\|UG\|Ta#S18007638\|564 | 193781131 | 193781131 | - |
| 6A | Blast | gnl\|UG\|Ta#S18007638\|585 | 193781110 | 193781110 | - |
| 6A | Blast | gnl\|UG\|Ta#S18007638\|676 | 193781019 | 193781019 | - |
| 6A | Blast | gnl\|UG\|Ta#S18007638\|702 | 193780993 | 193780993 | - |
| 6A | Blast | gnl\|UG\|Ta#S18007638\|706 | 193780989 | 193780989 | - |
| 6A | Blast | gnl\|UG\|Ta#S18008200\|530 | 193781165 | 193781165 | - |
| 6A | Blast | gnl\|UG\|Ta#S22378890\|130 | 170635689 | 170635689 | - |
| 6A | Blast | gnl\|UG\|Ta#S22391491\|171 | 10368158 | 10368158 | + |
| 6A | Blast | gnl\|UG\|Ta#S37748721\|155 | 201769676 | 201769676 | - |
| 6A | Blast | gnl\|UG\|Ta#S37748721\|169 | 201769662 | 201769662 | - |
| 6A | Blast | gnl\|UG\|Ta#S37748721\|482 | 201769349 | 201769349 | - |
| 6A | Blast | gnl\|UG\|Ta#S37748721\|488 | 201769343 | 201769343 | - |
| 6A | Blast | gnl\|UG\|Ta#S37748721\|502 | 201769329 | 201769329 | - |
| 6A | Blast | gnl\|UG\|Ta#S37748721\|505 | 201769326 | 201769326 | - |
| 6A | Blast | gnl\|UG\|Ta#S37748721\|639 | 201769192 | 201769192 | - |
| 6A | Blast | gnl\|UG\|Ta#S37748721\|681 | 201769150 | 201769150 | - |
| 6A | Blast | gnl\|UG\|Ta#S37748721\|690 | 201769141 | 201769141 | - |
| 6A | Blast | gnl\|UG\|Ta#S37748721\|703 | 201769128 | 201769128 | - |
| 6A | Blast | gnl\|UG\|Ta#S37748721\|716 | 201769115 | 201769115 | - |
| 6A | Blast | gnl\|UG\|Ta#S37748721\|719 | 201769112 | 201769112 | - |
| 6A | Blast | gnl\|UG\|Ta#S37748721\|720 | 201769111 | 201769111 | - |
| 6A | Blast | gnl\|UG\|Ta#S52544162\|1093 | 206741386 | 206741386 | - |
| 6A | Blast | gnl\|UG\|Ta#S52546904\|10 | 30094247 | 30094247 | + |
| 6A | Blast | gnl\|UG\|Ta#S52546904\|49 | 30094286 | 30094286 | + |
| 6A | Blast | gnl\|UG\|Ta#S52546904\|51 | 30094288 | 30094288 | + |
| 6A | Blast | gnl\|UG\|Ta#S52546904\|56 | 30094293 | 30094293 | + |
| 6A | Blast | gnl\|UG\|Ta#S61564558\|171 | 145815957 | 145815957 | - |
| 6A | Blast | gnl\|UG\|Ta#S61606082\|680 | 2966069 | 2966069 | - |
| 6A | Blast | gnl\|UG\|Ta#S61798409\|284 | 12220810 | 12220810 | + |
| 6A | Blast | gnl\|UG\|Ta#S61801084\|1134 | 201605406 | 201605406 | - |
| 6A | Blast | gnl\|UG\|Ta#S61809799\|1048 | 54647523 | 54647523 | + |
| 6A | Blast | gnl\|UG\|Ta#S61809799\|1226 | 54647701 | 54647701 | + |
| 6A | Blast | gnl\|UG\|Ta#S61809799\|271 | 54646746 | 54646746 | + |
| 6A | Blast | gnl\|UG\|Ta#S61809799\|302 | 54646777 | 54646777 | + |
| 6A | Blast | gnl\|UG\|Ta#S61809799\|568 | 54647043 | 54647043 | + |
| 6A | Blast | gnl\|UG\|Ta#S61809799\|688 | 54647163 | 54647163 | + |
| 6A | Blast | gnl\|UG\|Ta#S61809799\|959 | 54647434 | 54647434 | + |
| 6A | Blast | gnl\|UG\|Ta#S61832405\|1799 | 13751909 | 13751909 | - |
| 6B | Blast | gnl\|UG\|Ta#S17986052\|361 | 178835588 | 178835588 | - |
| 6B | Blast | gnl\|UG\|Ta#S52546627\|1400 | 37300370 | 37300370 | + |
| 6B | Blast | gnl\|UG\|Ta#S58899419\|443 | 200438689 | 200438689 | - |
| 6B | Blast | gnl\|UG\|Ta#S58911197\|778 | 180467483 | 180467483 | - |
| 6B | Blast | gnl\|UG\|Ta#S61528550\|481 | 177543163 | 177543163 | + |
| 6B | Blast | gnl\|UG\|Ta#S61580653\|1018 | 35395073 | 35395073 | - |
| 6B | Blast | gnl\|UG\|Ta#S61580653\|412 | 35395679 | 35395679 | - |
| 6B | Blast | gnl\|UG\|Ta#S61580653\|422 | 35395669 | 35395669 | - |
| 6B | Blast | gnl\|UG\|Ta#S61580653\|567 | 35395524 | 35395524 | - |
| 6B | Blast | gnl\|UG\|Ta#S61580653\|612 | 35395479 | 35395479 | - |
| 6B | Blast | gnl\|UG\|Ta#S61580653\|795 | 35395296 | 35395296 | - |
| 6B | Blast | gnl\|UG\|Ta#S61580653\|853 | 35395238 | 35395238 | - |
| 6B | Blast | gnl\|UG\|Ta#S61580653\|965 | 35395126 | 35395126 | - |
| 6B | Blast | gnl\|UG\|Ta#S61604968\|355 | 24826408 | 24826408 | - |
| 6B | Blast | gnl\|UG\|Ta#S61604968\|359 | 24826404 | 24826404 | - |
| 6B | Blast | gnl\|UG\|Ta#S61604968\|368 | 24826395 | 24826395 | - |
| 6B | Blast | gnl\|UG\|Ta#S61645712\|551 | 139820681 | 139820681 | + |
| 6B | Blast | gnl\|UG\|Ta#S61666611\|146 | 200452970 | 200452970 | - |
| 6B | Blast | gnl\|UG\|Ta#S61666611\|301 | 200452815 | 200452815 | - |
| 6B | Blast | gnl\|UG\|Ta#S61798828\|1045 | 18449339 | 18449339 | + |
| 6B | Blast | gnl\|UG\|Ta#S61798828\|142 | 18448436 | 18448436 | + |
| 6B | Blast | gnl\|UG\|Ta#S61798828\|267 | 18448561 | 18448561 | + |
| 6B | Blast | gnl\|UG\|Ta#S61798828\|403 | 18448697 | 18448697 | + |
| 6B | Blast | gnl\|UG\|Ta#S61798828\|409 | 18448703 | 18448703 | + |
| 6B | Blast | gnl\|UG\|Ta#S61798828\|418 | 18448712 | 18448712 | + |
| 6B | Blast | gnl\|UG\|Ta#S61798828\|727 | 18449021 | 18449021 | + |
| 6B | Blast | gnl\|UG\|Ta#S61798828\|847 | 18449141 | 18449141 | + |
| 6B | Blast | gnl\|UG\|Ta#S61798828\|96 | 18448390 | 18448390 | + |
| 6B | Blast | gnl\|UG\|Ta#S61807789\|613 | 100557174 | 100557174 | + |
| 6B | Blast | gnl\|UG\|Ta#S61828254\|1384 | 14695443 | 14695443 | + |
| 6B | Blast | gnl\|UG\|Ta#S61829555\|346 | 4594957 | 4594957 | - |
| 6B | Blast | gnl\|UG\|Ta#S61829555\|364 | 4594939 | 4594939 | - |
| 6B | Blast | gnl\|UG\|Ta#S61829555\|692 | 4594611 | 4594611 | - |
| 6B | Blast | gnl\|UG\|Ta#S61831113\|677 | 33894186 | 33894186 | + |
| 6B | Blast | gnl\|UG\|Ta#S61831578\|1193 | 29940583 | 29940583 | - |
| 6B | Blast | gnl\|UG\|Ta#S61831803\|2491 | 47491362 | 47491362 | + |
| 6B | Blast | gnl\|UG\|Ta#S61831803\|2511 | 47491382 | 47491382 | + |
| 6B | Blast | gnl\|UG\|Ta#S61831803\|2521 | 47491392 | 47491392 | + |
| 6B | Blast | gnl\|UG\|Ta#S61831803\|2534 | 47491405 | 47491405 | + |
| 6B | Blast | gnl\|UG\|Ta#S61832491\|2331 | 6259635 | 6259635 | - |
| 6B | Blast | gnl\|UG\|Ta#S65594812\|1093 | 12992226 | 12992226 | + |
| 6B | Blast | gnl\|UG\|Ta#S65594812\|1095 | 12992228 | 12992228 | + |
| 6B | Blast | gnl\|UG\|Ta#S65594812\|1331 | 12992464 | 12992464 | + |
| 6B | Blast | gnl\|UG\|Ta#S65594812\|1339 | 12992472 | 12992472 | + |
| 6B | Blast | gnl\|UG\|Ta#S65594812\|1371 | 12992504 | 12992504 | + |
| 6B | Blast | gnl\|UG\|Ta#S65594812\|1373 | 12992506 | 12992506 | + |
| 6B | Blast | gnl\|UG\|Ta#S65594812\|1404 | 12992537 | 12992537 | + |
| 6B | Blast | gnl\|UG\|Ta#S65594812\|1414 | 12992547 | 12992547 | + |
| 6B | Blast | gnl\|UG\|Ta#S65594812\|1420 | 12992553 | 12992553 | + |
| 6B | Blast | gnl\|UG\|Ta#S65594812\|1422 | 12992555 | 12992555 | + |
| 6B | Blast | gnl\|UG\|Ta#S65594812\|1474 | 12992607 | 12992607 | + |
| 6B | Blast | gnl\|UG\|Ta#S65594812\|1500 | 12992633 | 12992633 | + |
| 6B | Blast | gnl\|UG\|Ta#S65594812\|1503 | 12992636 | 12992636 | + |
| 6D | Blast | gnl\|UG\|Ta#S17987833\|209 | 65004951 | 65004951 | - |
| 6D | Blast | gnl\|UG\|Ta#S17987833\|419 | 65004741 | 65004741 | - |
| 6D | Blast | gnl\|UG\|Ta#S17987833\|524 | 65004636 | 65004636 | - |
| 6D | Blast | gnl\|UG\|Ta#S17987833\|619 | 65004541 | 65004541 | - |
| 6D | Blast | gnl\|UG\|Ta#S17987833\|871 | 65004289 | 65004289 | - |
| 6D | Blast | gnl\|UG\|Ta#S61575490\|588 | 156946759 | 156946759 | + |
| 6D | Blast | gnl\|UG\|Ta#S61583807\|684 | 162390181 | 162390181 | - |
| 6D | Blast | gnl\|UG\|Ta#S61597330\|362 | 159879506 | 159879506 | + |
| 6D | Blast | gnl\|UG\|Ta#S61597330\|369 | 159879513 | 159879513 | + |
| 6D | Blast | gnl\|UG\|Ta#S61597330\|470 | 159879614 | 159879614 | + |
| 6D | Blast | gnl\|UG\|Ta#S61597330\|497 | 159879641 | 159879641 | + |
| 6D | Blast | gnl\|UG\|Ta#S61597330\|542 | 159879686 | 159879686 | + |
| 6D | Blast | gnl\|UG\|Ta#S61597330\|572 | 159879716 | 159879716 | + |
| 6D | Blast | gnl\|UG\|Ta#S61597330\|641 | 159879785 | 159879785 | + |
| 6D | Blast | gnl\|UG\|Ta#S61597330\|843 | 159879987 | 159879987 | + |
| 6D | Blast | gnl\|UG\|Ta#S61597330\|959 | 159880103 | 159880103 | + |
| 6D | Blast | gnl\|UG\|Ta#S61620317\|773 | 162671784 | 162671784 | + |
| 6D | Blast | gnl\|UG\|Ta#S61624126\|522 | 173333162 | 173333162 | + |
| 6D | Blast | gnl\|UG\|Ta#S61701502\|80 | 168073237 | 168073237 | + |
| 6D | Blast | gnl\|UG\|Ta#S61780642\|157 | 161674857 | 161674857 | + |
| 6D | Blast | gnl\|UG\|Ta#S61807680\|882 | 3864861 | 3864861 | + |
| 6D | Blast | gnl\|UG\|Ta#S61807680\|891 | 3864870 | 3864870 | + |
| 6D | Blast | gnl\|UG\|Ta#S61807680\|912 | 3864891 | 3864891 | + |
| 6D | Blast | gnl\|UG\|Ta#S61809749\|1407 | 175664313 | 175664313 | - |
| 6D | Blast | gnl\|UG\|Ta#S61809981\|1172 | 11473532 | 11473532 | + |
| 6D | Blast | gnl\|UG\|Ta#S61812501\|1159 | 7528832 | 7528832 | + |
| 6D | Blast | gnl\|UG\|Ta#S61829177\|444 | 160972725 | 160972725 | + |
| 6D | Blast | gnl\|UG\|Ta#S61829177\|510 | 160972791 | 160972791 | + |
| 6D | Blast | gnl\|UG\|Ta#S61829177\|876 | 160973157 | 160973157 | + |
| 7A | Blast | gnl\|UG\|Ta#S19224533\|77 | 101352781 | 101352781 | + |
| 7A | Blast | gnl\|UG\|Ta#S22367905\|435 | 136746538 | 136746538 | + |
| 7A | Blast | gnl\|UG\|Ta#S22389506\|590 | 128717762 | 128717762 | - |
| 7A | Blast | gnl\|UG\|Ta#S26027323\|675 | 45815556 | 45815556 | - |
| 7A | Blast | gnl\|UG\|Ta#S36127280\|2198 | 20939895 | 20939895 | - |
| 7A | Blast | gnl\|UG\|Ta#S37902031\|360 | 136071120 | 136071120 | - |
| 7A | Blast | gnl\|UG\|Ta#S37928342\|174 | 93893813 | 93893813 | + |
| 7A | Blast | gnl\|UG\|Ta#S37928342\|290 | 93893929 | 93893929 | + |
| 7A | Blast | gnl\|UG\|Ta#S52543087\|1049 | 7214503 | 7214503 | - |
| 7A | Blast | gnl\|UG\|Ta#S52546679\|2375 | 178995242 | 178995242 | + |
| 7A | Blast | gnl\|UG\|Ta#S52546679\|2500 | 178995367 | 178995367 | + |
| 7A | Blast | gnl\|UG\|Ta#S52546679\|2524 | 178995391 | 178995391 | + |
| 7A | Blast | gnl\|UG\|Ta#S58907191\|3149 | 160573626 | 160573626 | - |
| 7A | Blast | gnl\|UG\|Ta#S58907191\|3620 | 160573155 | 160573155 | - |
| 7A | Blast | gnl\|UG\|Ta#S58907191\|4022 | 160572753 | 160572753 | - |
| 7A | Blast | gnl\|UG\|Ta#S58907191\|4428 | 160572347 | 160572347 | - |
| 7A | Blast | gnl\|UG\|Ta#S58907191\|855 | 160575920 | 160575920 | - |
| 7A | Blast | gnl\|UG\|Ta#S61520328\|579 | 77343750 | 77343750 | - |
| 7A | Blast | gnl\|UG\|Ta#S61560974\|585 | 159407790 | 159407790 | + |
| 7A | Blast | gnl\|UG\|Ta#S61769938\|892 | 97852894 | 97852894 | - |
| 7A | Blast | gnl\|UG\|Ta#S61799161\|732 | 161401410 | 161401410 | - |
| 7A | Blast | gnl\|UG\|Ta#S61809326\|1717 | 97502374 | 97502374 | - |
| 7A | Blast | gnl\|UG\|Ta#S61809326\|1764 | 97502327 | 97502327 | - |
| 7A | Blast | gnl\|UG\|Ta#S61811309\|1809 | 12242948 | 12242948 | + |
| 7A | Blast | gnl\|UG\|Ta#S61811309\|2210 | 12243349 | 12243349 | + |
| 7A | Blast | gnl\|UG\|Ta#S61811669\|1001 | 78287402 | 78287402 | + |
| 7A | Blast | gnl\|UG\|Ta#S61811669\|1440 | 78287841 | 78287841 | + |
| 7A | Blast | gnl\|UG\|Ta#S61811669\|1457 | 78287858 | 78287858 | + |
| 7A | Blast | gnl\|UG\|Ta#S61811669\|2225 | 78288626 | 78288626 | + |
| 7A | Blast | gnl\|UG\|Ta#S61811669\|2440 | 78288841 | 78288841 | + |
| 7A | Blast | gnl\|UG\|Ta#S61811725\|2458 | 1304995 | 1304995 | - |
| 7A | Blast | gnl\|UG\|Ta#S61827587\|174 | 158069883 | 158069883 | - |
| 7A | Blast | gnl\|UG\|Ta#S61829096\|1250 | 158604783 | 158604783 | - |
| 7A | Blast | gnl\|UG\|Ta#S61830105\|1598 | 105268556 | 105268556 | + |
| 7A | Blast | gnl\|UG\|Ta#S61830785\|1058 | 103780821 | 103780821 | + |
| 7A | Blast | gnl\|UG\|Ta#S61830785\|572 | 103780335 | 103780335 | + |
| 7A | Blast | gnl\|UG\|Ta#S61831323\|1512 | 162316957 | 162316957 | - |
| 7A | Blast | gnl\|UG\|Ta#S61841121\|593 | 98697471 | 98697471 | + |
| 7A | Blast | gnl\|UG\|Ta#S65611405\|335 | 39972597 | 39972597 | + |
| 7A | Blast | gnl\|UG\|Ta#S65620759\|196 | 139485726 | 139485726 | - |
| 7A | Blast | gnl\|UG\|Ta#S65631079\|962 | 168488704 | 168488704 | - |
| 7B | Blast | gnl\|UG\|Ta#S18006083\|352 | 175302807 | 175302807 | + |
| 7B | Blast | gnl\|UG\|Ta#S61602705\|802 | 215873062 | 215873062 | + |
| 7B | Blast | gnl\|UG\|Ta#S61602705\|930 | 215873190 | 215873190 | + |
| 7B | Blast | gnl\|UG\|Ta#S61610515\|774 | 170975136 | 170975136 | + |
| 7B | Blast | gnl\|UG\|Ta#S61648627\|252 | 219419572 | 219419572 | - |
| 7B | Blast | gnl\|UG\|Ta#S61648627\|267 | 219419557 | 219419557 | - |
| 7B | Blast | gnl\|UG\|Ta#S61648627\|342 | 219419482 | 219419482 | - |
| 7B | Blast | gnl\|UG\|Ta#S61648627\|393 | 219419431 | 219419431 | - |
| 7B | Blast | gnl\|UG\|Ta#S61648627\|423 | 219419401 | 219419401 | - |
| 7B | Blast | gnl\|UG\|Ta#S61648627\|426 | 219419398 | 219419398 | - |
| 7B | Blast | gnl\|UG\|Ta#S61648627\|476 | 219419348 | 219419348 | - |
| 7B | Blast | gnl\|UG\|Ta#S61648627\|479 | 219419345 | 219419345 | - |
| 7B | Blast | gnl\|UG\|Ta#S61648627\|480 | 219419344 | 219419344 | - |
| 7B | Blast | gnl\|UG\|Ta#S61648627\|535 | 219419289 | 219419289 | - |
| 7B | Blast | gnl\|UG\|Ta#S61648627\|537 | 219419287 | 219419287 | - |
| 7B | Blast | gnl\|UG\|Ta#S61648627\|547 | 219419277 | 219419277 | - |
| 7B | Blast | gnl\|UG\|Ta#S61650486\|215 | 176030765 | 176030765 | - |
| 7B | Blast | gnl\|UG\|Ta#S61650486\|499 | 176030481 | 176030481 | - |
| 7B | Blast | gnl\|UG\|Ta#S61782347\|1572 | 26045356 | 26045356 | - |
| 7B | Blast | gnl\|UG\|Ta#S61810913\|240 | 146025020 | 146025020 | - |
| 7B | Blast | gnl\|UG\|Ta#S65614450\|187 | 165042021 | 165042021 | - |
| 7B | Blast | gnl\|UG\|Ta#S65614450\|228 | 165041980 | 165041980 | - |
| 7B | Blast | gnl\|UG\|Ta#S65614450\|272 | 165041936 | 165041936 | - |
| 7B | Blast | gnl\|UG\|Ta#S65614450\|312 | 165041896 | 165041896 | - |
| 7B | Blast | gnl\|UG\|Ta#S65614450\|393 | 165041815 | 165041815 | - |
| 7B | Blast | gnl\|UG\|Ta#S65614450\|401 | 165041807 | 165041807 | - |
| 7D | Blast | gnl\|UG\|Ta#S16058462\|1513 | 185716778 | 185716778 | + |
| 7D | Blast | gnl\|UG\|Ta#S32593336\|594 | 100248152 | 100248152 | + |
| 7D | Blast | gnl\|UG\|Ta#S32719796\|121 | 201149955 | 201149955 | - |
| 7D | Blast | gnl\|UG\|Ta#S32719796\|301 | 201149775 | 201149775 | - |
| 7D | Blast | gnl\|UG\|Ta#S37920838\|332 | 20027854 | 20027854 | + |
| 7D | Blast | gnl\|UG\|Ta#S52546422\|1417 | 115559961 | 115559961 | + |
| 7D | Blast | gnl\|UG\|Ta#S52546422\|205 | 115558749 | 115558749 | + |
| 7D | Blast | gnl\|UG\|Ta#S52546422\|888 | 115559432 | 115559432 | + |
| 7D | Blast | gnl\|UG\|Ta#S58868328\|193 | 128346399 | 128346399 | + |
| 7D | Blast | gnl\|UG\|Ta#S58885223\|166 | 13450289 | 13450289 | - |
| 7D | Blast | gnl\|UG\|Ta#S58885223\|173 | 13450282 | 13450282 | - |
| 7D | Blast | gnl\|UG\|Ta#S58892376\|398 | 165359904 | 165359904 | - |
| 7D | Blast | gnl\|UG\|Ta#S58906682\|123 | 80801355 | 80801355 | + |
| 7D | Blast | gnl\|UG\|Ta#S58910751\|1821 | 1273344 | 1273344 | + |
| 7D | Blast | gnl\|UG\|Ta#S58910751\|1841 | 1273364 | 1273364 | + |
| 7D | Blast | gnl\|UG\|Ta#S58910751\|1870 | 1273393 | 1273393 | + |
| 7D | Blast | gnl\|UG\|Ta#S58910751\|1916 | 1273439 | 1273439 | + |
| 7D | Blast | gnl\|UG\|Ta#S58910751\|1917 | 1273440 | 1273440 | + |
| 7D | Blast | gnl\|UG\|Ta#S58910751\|1929 | 1273452 | 1273452 | + |
| 7D | Blast | gnl\|UG\|Ta#S58910751\|1950 | 1273473 | 1273473 | + |
| 7D | Blast | gnl\|UG\|Ta#S58910751\|1959 | 1273482 | 1273482 | + |
| 7D | Blast | gnl\|UG\|Ta#S58910751\|1963 | 1273486 | 1273486 | + |
| 7D | Blast | gnl\|UG\|Ta#S58910751\|1984 | 1273507 | 1273507 | + |
| 7D | Blast | gnl\|UG\|Ta#S58910751\|1988 | 1273511 | 1273511 | + |
| 7D | Blast | gnl\|UG\|Ta#S58910751\|2007 | 1273530 | 1273530 | + |
| 7D | Blast | gnl\|UG\|Ta#S61559278\|477 | 74414186 | 74414186 | - |
| 7D | Blast | gnl\|UG\|Ta#S61559278\|47 | 74414616 | 74414616 | - |
| 7D | Blast | gnl\|UG\|Ta#S61610092\|838 | 138121704 | 138121704 | + |
| 7D | Blast | gnl\|UG\|Ta#S61618501\|397 | 214821775 | 214821775 | + |
| 7D | Blast | gnl\|UG\|Ta#S61756902\|460 | 208636735 | 208636735 | + |
| 7D | Blast | gnl\|UG\|Ta#S61810383\|715 | 69694688 | 69694688 | - |
| 7D | Blast | gnl\|UG\|Ta#S61810383\|755 | 69694648 | 69694648 | - |
| 7D | Blast | gnl\|UG\|Ta#S61811206\|1676 | 18613145 | 18613145 | + |
| 7D | Blast | gnl\|UG\|Ta#S61811206\|1736 | 18613205 | 18613205 | + |
| 7D | Blast | gnl\|UG\|Ta#S61811206\|1745 | 18613214 | 18613214 | + |
| 7D | Blast | gnl\|UG\|Ta#S61811206\|1961 | 18613430 | 18613430 | + |
| 7D | Blast | gnl\|UG\|Ta#S65618237\|330 | 1857915 | 1857915 | - |
| 7D | Blast | gnl\|UG\|Ta#S65642589\|698 | 1268537 | 1268537 | - |
