# Supplementary material for: SNP Discovery Using BSR-Seq Approach for Spot Blotch Resistance in Wheat (Triticum aestivum L.), an Essential Crop for Food Security
Source: Front Genet. 2022 Apr 5;13:859676. doi: 10.3389/fgene.2022.859676 (PMC9016274; doi:10.3389/fgene.2022.859676)
Supplement: Supplementary file 3 [file Presentation1.PPTX]

## Slide 1
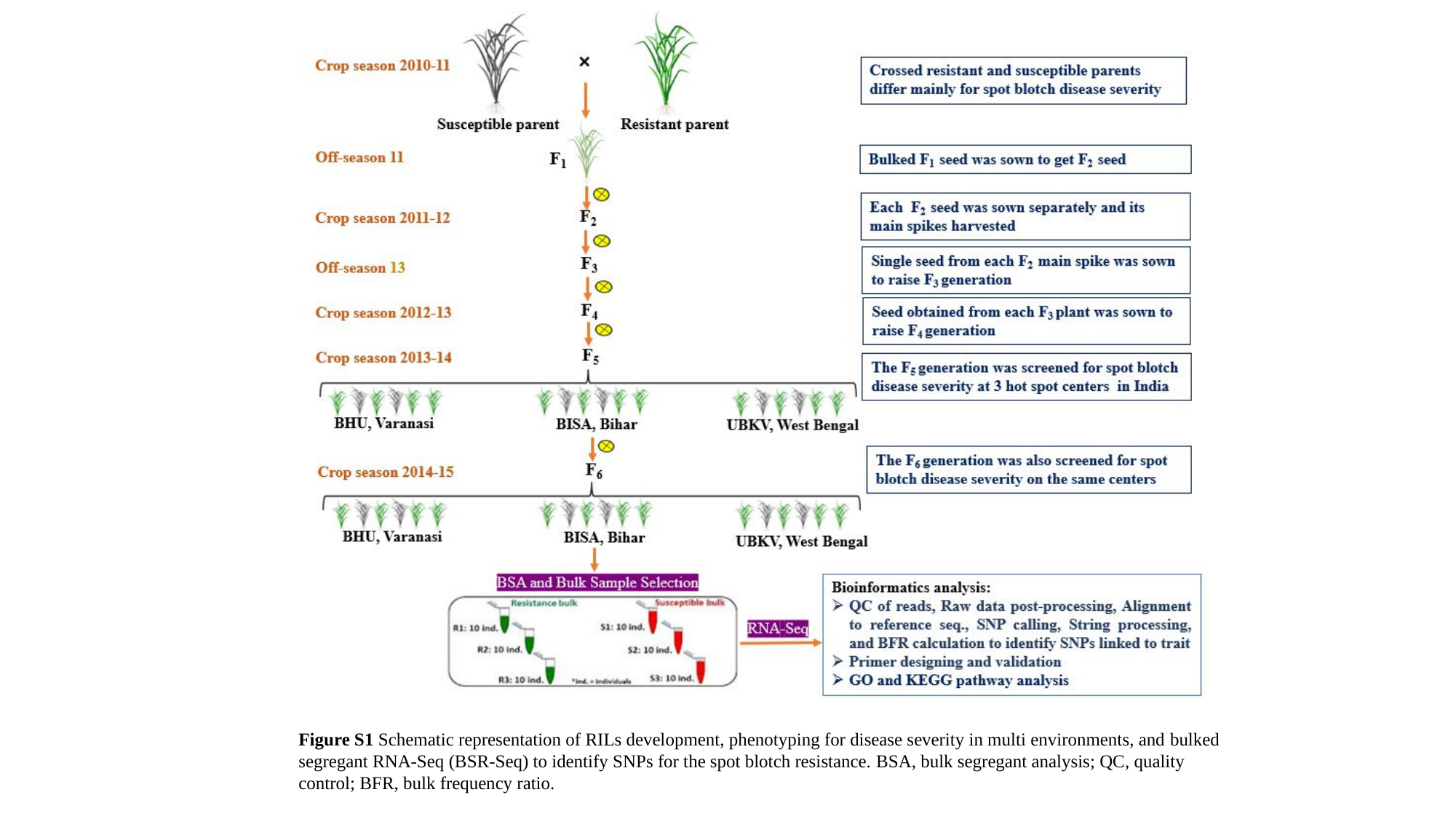

Figure S1 Schematic representation of RILs development, phenotyping for disease severity in multi environments, and bulked segregant RNA-Seq (BSR-Seq) to identify SNPs for the spot blotch resistance. BSA, bulk segregant analysis; QC, quality control; BFR, bulk frequency ratio.

## Slide 2
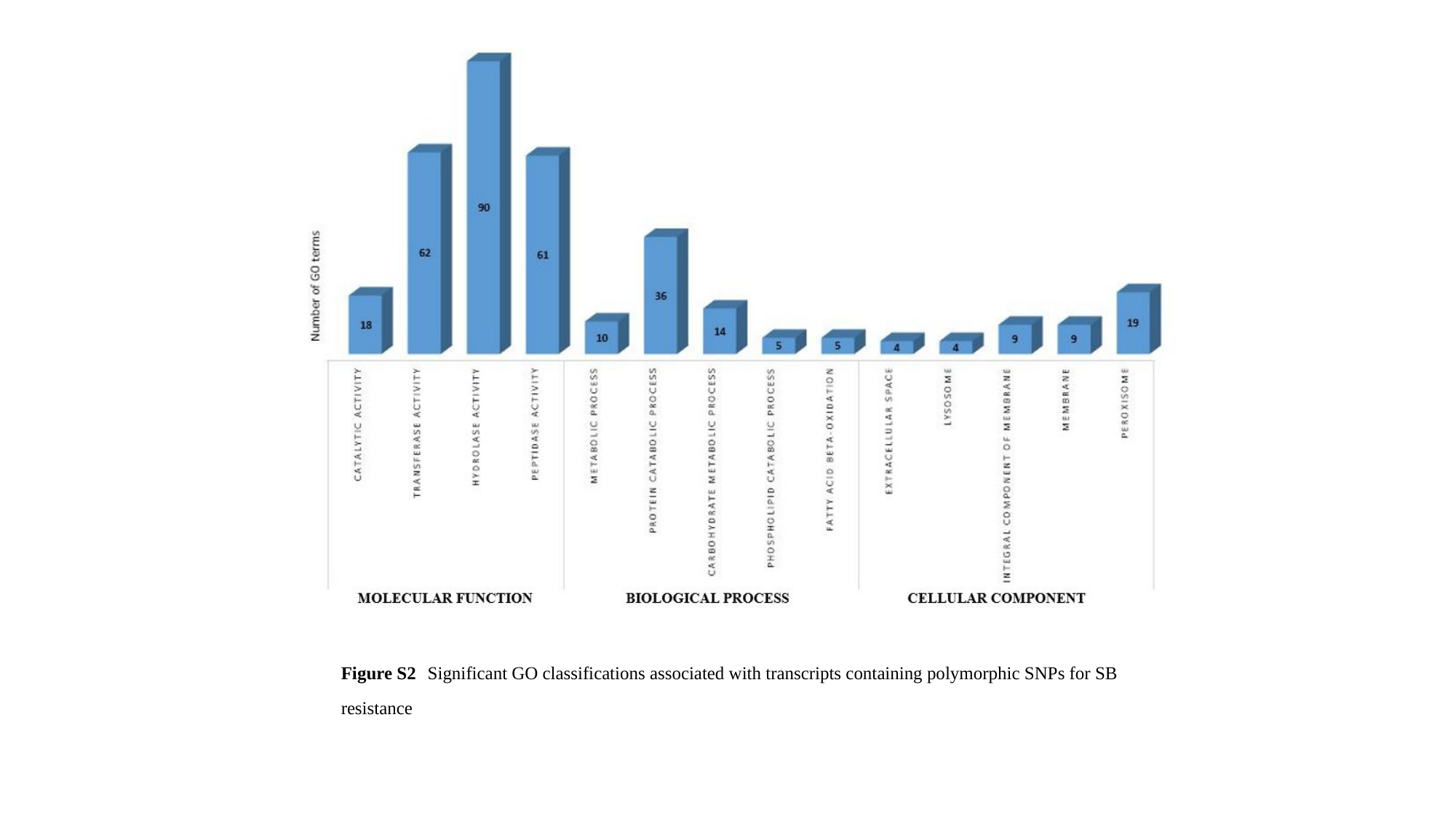

Figure S2 Significant GO classifications associated with transcripts containing polymorphic SNPs for SB resistance

## Slide 3
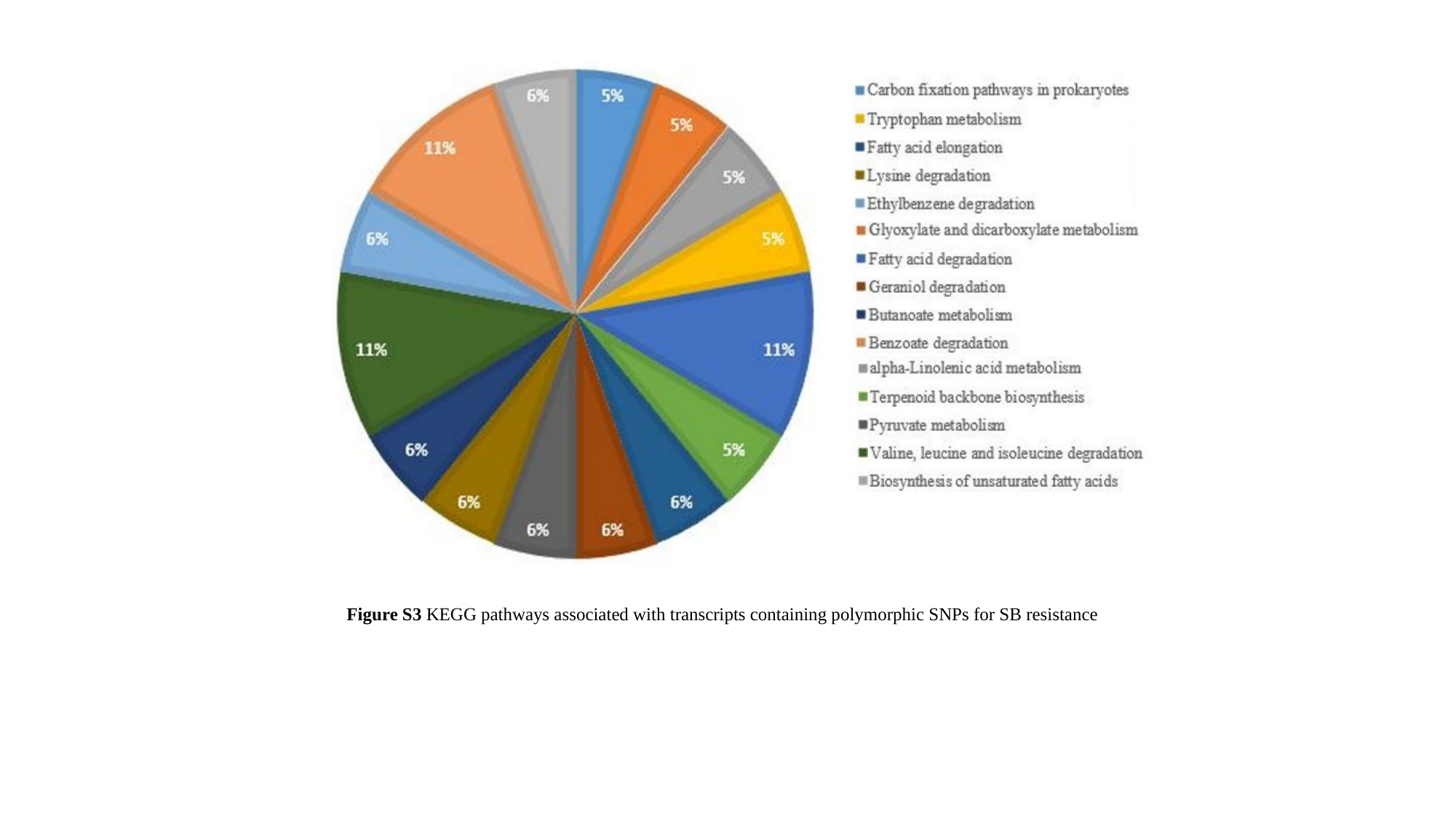

Figure S3 KEGG pathways associated with transcripts containing polymorphic SNPs for SB resistance
