# Supplementary material for: SNP Discovery Using BSR-Seq Approach for Spot Blotch Resistance in Wheat (Triticum aestivum L.), an Essential Crop for Food Security
Source: Front Genet. 2022 Apr 5;13:859676. doi: 10.3389/fgene.2022.859676 (PMC9016274; doi:10.3389/fgene.2022.859676)
Supplement: Supplementary file 4 [file Table2.DOCX]

| **Table S2** Primer information of 3B and 5B chromosome, used for the development of SNPs based assay for SB resistance. | | | | | | |
| --- | --- | --- | --- | --- | --- | --- |
| Locus name | Type | Orientation | Primer sequence | Allele | Temp. | Product size (bp) |
|  |  |  | 3B primer-1 |  |  |  |
| Ta_S61799095_1237 | Outer primer | Forward | CTCATTGCTCTGGACGGCATAAGCAT |  | 70.06 | 312 |
|  | Outer primer | Reverse | TGCACCACCATCAACAAAGTCTGCTC |  | 70.39 |  |
|  | Inner primer | Forward | ACCCTCGCCTTGCCGTTTCG | G | 70.75 | 154 |
|  | Inner primer | Reverse | GCATGTCCAATGCCCCCAAATACTCT | A | 70.42 | 203 |
| 3B primer-2 | | | | | | |
| Ta_S61799095_972 | Outer primer | Forward | CCATTCTTCTTAAAAGCCGGTCC |  | 64.16 | 178 |
|  | Outer primer | Reverse | AGAGCAATGAGCATGGAATAGCAG |  | 63.98 |  |
|  | Inner primer | Forward | GCAAGACTCTCATCTTTGTCAACCAC | C | 64.58 | 124 |
|  | Inner primer | Reverse | GTAGAGGCAGACCGCCAGGTA | T | 63.82 | 100 |
| 5B primer-1 | | | | | | |
| Ta_S61830716_1262_3 | Outer primer | Forward | CCTTCGGTCCTCCACAGCCT |  | 66.11 | 197 |
|  | Outer primer | Reverse | GGGACGCCAATCACAATCAGTACAT |  | 67.15 |  |
|  | Inner primer | Forward | GCAGCTTTGTCAATCGTCCTTCTAAA | A | 65.76 | 142 |
|  | Inner primer | Reverse | ACGGCTAAGATCGCTGACCCC | G | 66.88 | 101 |
| 5B primer-2 | | | | | | |
| Ta_S17985740_315 | Outer primer | Forward | CTAGCGATACACCGAAACCAAGATCG |  | 67.65 | 209 |
|  | Outer primer | Reverse | TACTGGATCATCAAGAACTCATGGGG |  | 66.36 |  |
|  | Inner primer | Forward | TACTCCTCCTTCGAGGCGTTCG | G | 67.02 | 146 |
|  | Inner primer | Reverse | GTCTCCACGGTGTCCGCAAT | A | 65.68 | 104 |
